# Geography of Genetic Structure in Barley Wild Relative *Hordeum vulgare* subsp. *spontaneum* in Jordan

**DOI:** 10.1371/journal.pone.0160745

**Published:** 2016-08-11

**Authors:** Imke Thormann, Patrick Reeves, Ann Reilley, Johannes M. M. Engels, Ulrike Lohwasser, Andreas Börner, Klaus Pillen, Christopher M. Richards

**Affiliations:** 1 Bioversity International, Rome, Italy; 2 National Center for Genetic Resources Preservation, United States Department of Agriculture-Agricultural Research Service, Fort Collins, Colorado, United States of America; 3 Genebank Department, Leibniz Institute of Plant Genetics and Crop Plant Research, Gatersleben, Germany; 4 Plant Breeding, Institute for Agricultural and Nutritional Science, Martin-Luther-University Halle-Wittenberg, Halle, Germany; GERMANY

## Abstract

Informed collecting, conservation, monitoring and utilization of genetic diversity requires knowledge of the distribution and structure of the variation occurring in a species. *Hordeum vulgare* subsp. *spontaneum* (K. Koch) Thell., a primary wild relative of barley, is an important source of genetic diversity for barley improvement and co-occurs with the domesticate within the center of origin. We studied the current distribution of genetic diversity and population structure in *H*. *vulgare* subsp. *spontaneum* in Jordan and investigated whether it is correlated with either spatial or climatic variation inferred from publically available climate layers commonly used in conservation and ecogeographical studies. The genetic structure of 32 populations collected in 2012 was analyzed with 37 SSRs. Three distinct genetic clusters were identified. Populations were characterized by admixture and high allelic richness, and genetic diversity was concentrated in the northern part of the study area. Genetic structure, spatial location and climate were not correlated. This may point out a limitation in using large scale climatic data layers to predict genetic diversity, especially as it is applied to regional genetic resources collections in *H*. *vulgare* subsp. *spontaneum*.

## Introduction

Crop wild relatives (CWR) are vital for food security because they provide novel alleles for crop improvement and adaptation [1–3]. Their diversity is threatened by global and climate change [4,5], and more knowledge about the geographical distribution of their genetic variation, and the processes that shape it, is required to more effectively collect, conserve, monitor and use this variation. Genetic data are still lacking for many CWR. Ecogeographical information, which combines environmental and spatial data, is increasingly used as a proxy for genetic diversity to improve collecting, conservation, monitoring and use of CWR [6–11]. This approach assumes that ecogeographical diversity among collecting sites is correlated with genetic diversity because the distribution of genetic variation in wild plant species is affected by environment (via natural selection) and geographical separation (via isolation by distance). It follows that conserving populations sampled from the widest possible range of ecogeographical conditions is expected to maximize the genetic diversity conserved [12].

Ecogeographical data has been used to identify areas and populations for *in situ* conservation [7,10,13], to assemble core collections [14] and to identify germplasm potentially useful for crop improvement [15,16]. Habitat suitability modelling, also known as species distribution modelling or niche modelling, has been used to identify gaps in existing collections and to prioritize areas for collecting [8,17–19]. Habitat suitability modelling predicts the potential geographical distribution of a species using the known distribution and environmental data, which often come in the form of climatic, edaphic, geophysical and/or land use variables.

Maxted *et al*. [19] have cautioned that the expected correlation between genetic and ecogeographical diversity may not hold for all species and habitats. CWR are often found in ruderal areas and agricultural landscapes where natural, adaptive responses to climate might be altered through anthropogenic influences [20–23]. Of these, the breakdown of isolation by distance due to elevated gene flow may be particularly important.

Barley is the fourth most important cereal crop worldwide in terms of production, yield and area harvested, and is one of the crops in which CWR use in breeding programs is particularly prominent [24]. *Hordeum vulgare* subsp. *spontaneum* (K. Koch) Thell. (hereafter *Spontaneum*) is the progenitor of barley and represents an important genetic resource in barley breeding for traits such as powdery mildew, leaf scald or leaf rust resistance [25–31], yield [32], drought and temperature tolerance [33,34] and agronomic traits such as malting quality [35,36]. Recently, a multi-parental nested association mapping population, using 24 *Spontaneum* donor accessions to induce genetic variation, was set up and tested to study regulation of flowering time in barley [37]. The Fertile Crescent has been considered the primary center of origin and domestication of barley [38,39]. Other studies suggest additional domestication events in areas east of the Fertile Crescent [40], Tibet [41], Ethiopia and the Western Mediterranean [42,43].

Efforts have been made since the 1970s to characterize wild barley germplasm across its distribution range using morphological characters, isozymes and molecular markers [44–55]. The highest genetic variation lies within the Fertile Crescent, and there specifically in Jordan and Israel [55,56]. A few studies have compared diversity in *Spontaneum* between Jordan and neighboring countries. Baek *et al*. [57] found that the number of alleles as well as the percentage of country specific alleles is significantly higher in Jordan than in Israel. Analysis of SNP diversity indicated Jordan and southern Syria as a likely site of domestication [54].

Past studies have investigated the correlation between genetic diversity and environment in *Spontaneum*. They have documented an association between genetic diversity, at single loci, and geography, acrosstemperature or rainfall gradients [44,49,52,58–61]. Genetic differentiation has also been shown to occur, in sympatry, between opposing slopes in the Evolution Canyon in Israel. This has been attributed to adaptation to different microclimates [62,63]. In Jordan, Jaradat [64] characterized kernel protein content and genetic diversity at four esterase loci in 12 wild populations. Ribosomal DNA (rDNA) polymorphism was used to study accessions from 27 collecting sites [65]. The distribution of alleles was found to be correlated with ecogeographical factors such as rainfall, temperature, and geographical location. Baek *et al*. [57] used 18 SSRs to study genetic diversity in accessions from 16 collecting sites and reported associations between ecogeographical variables and allele frequencies at individual loci. Hübner *et al*. [66] studied *Spontaneum* in Israel and attempted to correlate genetic population structure—as opposed to polymorphism or allele frequencies at individual loci—with climate variables. No studies of the correlation between environment and population structure of *Spontaneum* in Jordan have yet been published.

In this study, we sampled *Spontaneum* populations across their range in Jordan and analyzed this collection with a set of 37 SSRs. Our aim was to describe the patterns of genetic diversity and population structure of Jordanian *Spontaneum* and to determine the degree to which the genetic structure estimated with our markers is correlated with spatial and climatic variables derived from global data sources commonly used in conservation and ecogeographical studies [18,67–69].

## Material and Methods

### Plant material and germination

**Fig 1:**
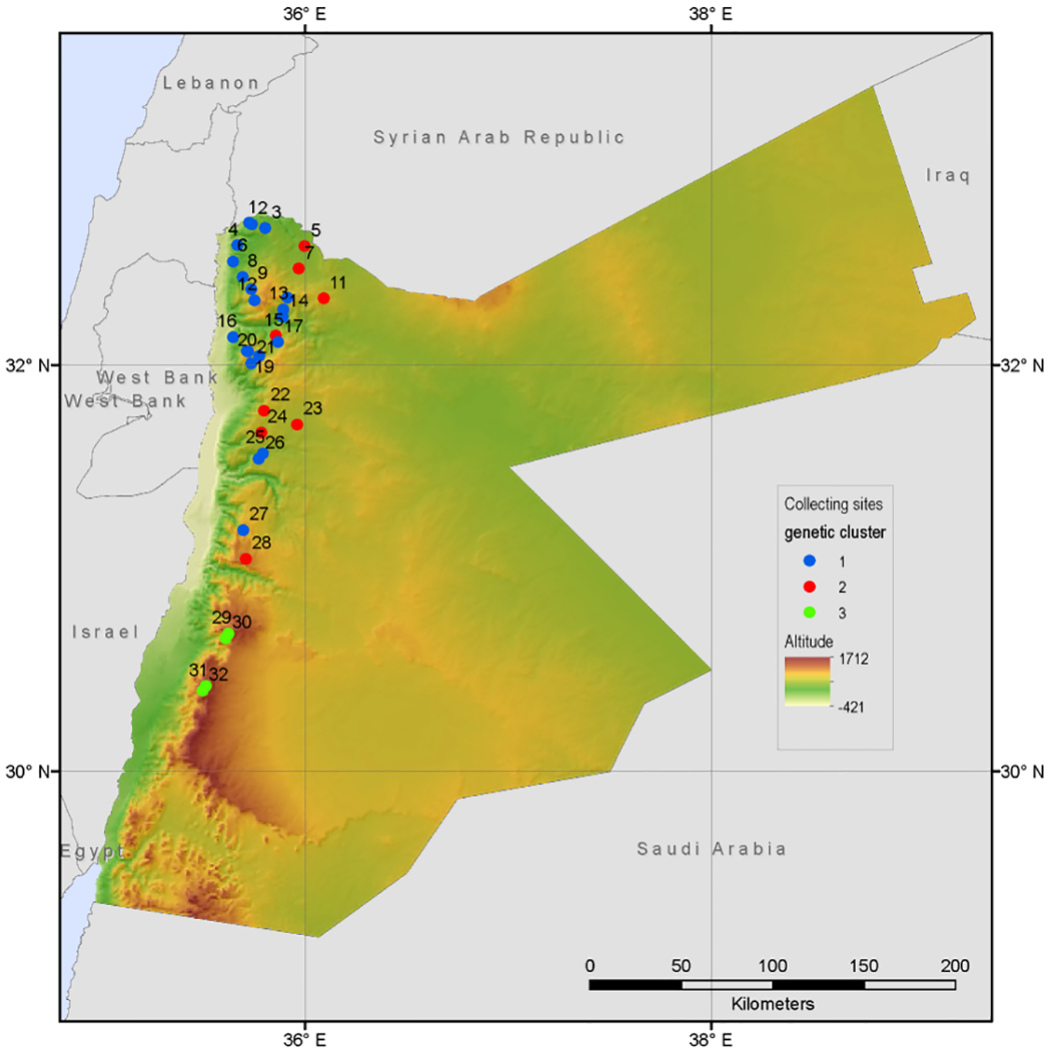
Collecting sites in Jordan.

Single spikes of 12–15 individuals were collected from each of 42 *Spontaneum* populations during a barley collecting mission carried out in 2012, which covered the entire distribution of *Spontaneum* in Jordan. The collecting had been formalized in a letter of agreement between the Jordanian National Center for Agricultural Research and Extension (NCARE) and Bioversity International, which encompassed the permit to collect *Spontaneum* from all visited sites. The collecting was carried out with the continuous participation of NCARE staff and no rare or threatened species was collected. Seeds from each spike were germinated to produce leaf tissue for DNA extraction. Up to eight seeds per spike were rolled into germination paper and placed in an incubator at 25°C for germination. 50–100 mg of 3–5 day old leaf tissue was harvested from one germinated seed per spike. 32 populations (Table 1) (where leaf tissue was available from at least 11 individuals) were used for the study. This resulted in a total of 373 genotypes, with 8–13 individuals per population (S1 Table). The spatial distribution of populations is shown in Fig 1.

**Table 1:** Collecting site description.

Collecting site number	Latitude	Longitude	Elevation (m)	Number of individuals used in study
1	32.70233	35.72325	94	12
2	32.69611	35.737528	119	13
3	32.67656	35.804833	467	11
4	32.59239	35.666944	87	11
5	32.58686	35.998194	475	12
6	32.51183	35.645444	119	12
7	32.47747	35.969694	608	12
8	32.43733	35.691806	484	12
9	32.37594	35.7365	785	12
10	32.33386	35.91375	957	11
11	32.32931	36.092667	866	12
12	32.32144	35.750139	770	12
13	32.27547	35.891333	564	11
14	32.23847	35.889472	379	13
15	32.14508	35.856639	561	12
16	32.13858	35.646806	141	12
17	32.11461	35.86625	629	14
18	32.06989	35.715139	1044	11
19	32.06694	35.720583	1058	12
20	32.04581	35.775167	911	12
21	32.01156	35.733778	589	11
22	31.77919	35.798833	805	9
23	31.70953	35.960611	709	13
24	31.67036	35.785889	745	12
25	31.56639	35.791417	646	11
26	31.54019	35.773639	677	11
27	31.18831	35.696583	773	12
28	31.04872	35.708861	1222	12
29	30.68108	35.622222	1566	12
30	30.65542	35.610194	1257	12
31	30.42178	35.512	1580	8
32	30.39875	35.496861	1680	11

### Ecogeographical and climate data of collecting sites

Geographical coordinates, altitude, slope, and aspect of the collecting sites were recorded with a GPS Garmin Emap device (datum: WGS84) and habitat type was recorded. Climate data was obtained from the WorldClim database version 1.4 (http://www.worldclim.org), a global and freely available source for climate data layers generated through interpolation of average monthly climate data from weather stations [70]. Layers for current climate conditions (1950–2000) for the 19 bioclimatic variables (Bioclim; see Table 2) were downloaded. Values for the 19 variables were extracted for each collecting site using DIVA-GIS. Collecting sites included ruderal habitats, barley field margins as well as nature reserves, covered an altitudinal range from 87 to 1680 m, a latitudinal range from 30.39875–32.70233 decimal degrees, a longitudinal range from 35.49686111–36.09266667 decimal degrees, an annual precipitation range from 229–491 mm and a mean annual temperature range from 12.5–21.5°C. Collecting site information is provided in S2 Table.

**Table 2:** Coding of bioclimatic variables according to WorldClim at http://www.worldclim.org/bioclim.

Code	Description
Bioclim 1	Annual Mean Temperature
Bioclim 2	Mean Diurnal Range (Mean of monthly (max temp—min temp))
Bioclim 3	Isothermality (Bioclim2/ Bioclim7) (* 100)
Bioclim 4	Temperature Seasonality (standard deviation *100)
Bioclim 5	Max Temperature of Warmest Month
Bioclim 6	Min Temperature of Coldest Month
Bioclim 7	Temperature Annual Range (Bioclim5- Bioclim6)
Bioclim 8	Mean Temperature of Wettest Quarter
Bioclim 9	Mean Temperature of Driest Quarter
Bioclim 10	Mean Temperature of Warmest Quarter
Bioclim 11	Mean Temperature of Coldest Quarter
Bioclim 12	Annual Precipitation
Bioclim 13	Precipitation of Wettest Month
Bioclim 14	Precipitation of Driest Month
Bioclim 15	Precipitation Seasonality (Coefficient of Variation)
Bioclim 16	Precipitation of Wettest Quarter
Bioclim 17	Precipitation of Driest Quarter
Bioclim 18	Precipitation of Warmest Quarter
Bioclim 19	Precipitation of Coldest Quarter

### DNA extraction and genotyping

DNA was purified from 3–5 day old leaf tissue with the Qiagen DNeasy^®^ 96 Plant Kit. Thirty-seven EST-derived SSR primers were used for genotyping [71–73] (Table 3). Loci were distributed across all 7 barley chromosomes. PCR was carried out in 5-μl reactions consisting of 2–10 ng genomic DNA, 1x Qiagen Multiplex PCR Master Mix, 225 nM of each primer pair. All fragments were amplified using MJ Research (Waltham, Massachusetts) PTC200 thermocyclers and the following PCR profile: an initial denaturing step of 15 min at 95°C followed by 40 cycles with denaturation at 94°C for 60 s, annealing at 60°C for 30 s and extension at 72°C for 15 s. After 40 cycles, a final extension step was performed at 72°C for 10 min. Amplification products were resolved by capillary electrophoresis on the ABI 3130xl Genetic Analyzer. Fragment sizes were calculated using GeneScan 400HD (ROX) internal size standards and scored with GeneMapper software (v. 5.0) (Life Technologies, Thermo Fisher Scientific Inc.).

**Table 3:** Characteristics of SSR markers.

Marker ID	Location	Ind no.	Allele no.	PIC	Allelic richness
GBM1002	1H	367	12	0.736	5.44
GBM1013	1H	373	7	0.383	3.194
GBM1029	1H	373	6	0.412	3.038
GBM1334	1H	372	5	0.538	3.317
GBM1461	1H	373	20	0.914	9.082
GBM1035	2H	373	6	0.66	4.264
GBM1036	2H	372	5	0.497	3.289
GBM1047	2H	364	7	0.664	4.269
GBM1208	2H	367	7	0.563	3.693
GBM1218	2H	369	4	0.591	3.593
GBM1459	2H	373	6	0.578	3.931
GBM1043	3H	372	5	0.495	3.735
GBM1110	3H	373	11	0.761	5.362
GBM1280	3H	372	5	0.661	4.038
GBM1405	3H	372	8	0.792	5.795
GBM1413	3H	372	5	0.517	3.171
GBM1003	4H	372	9	0.713	5.164
GBM1015	4H	373	19	0.834	7.016
GBM1020	4H	371	7	0.663	3.96
GBM1323	4H	371	7	0.642	4.535
GBM1026	5H	372	4	0.412	2.379
GBM1054	5H	368	7	0.682	4.822
GBM1064	5H	373	5	0.531	3.263
GBM1176	5H	373	7	0.58	3.892
GBM1363	5H	373	5	0.387	2.817
GBM1008	6H	366	9	0.75	5
GBM1021	6H	367	15	0.87	7.503
GBM1063	6H	373	10	0.746	5.26
GBM1075	6H	370	5	0.34	2.792
GBM1212	6H	372	4	0.376	2.208
GBM1404	6H	373	3	0.359	2.453
GBM1033	7H	373	6	0.687	4.268
GBM1060	7H	370	4	0.528	3.008
GBM1326	7H	373	10	0.816	5.926
GBM1419	7H	373	8	0.7	4.675
GBM1464	7H	365	22	0.888	8.074
GBM1516	7H	373	6	0.593	4.033
**Mean**		371	7.9	0.6	4.4

The following values are presented for each marker: chromosome location (Location), number of individuals scored (Ind no.), number of alleles (Allele no.), polymorphism information content (PIC), allelic richness.

### Genetic diversity

Summary statistics of the marker data such as number of alleles, sample adjusted allelic richness, and observed heterozygosity were calculated with GDA [74] and FSTAT version 2.93.2 [75]. The number of multi-locus genotypes was determined with GeneticStudio (http://dyerlab.bio.vcu.edu). Polymorphism information content (PIC) per locus was calculated with PICcalc [76].

### Population differentiation among sites

F_ST_ was used to measure differentiation between populations and was calculated with FSTAT. Inter-individual distances were calculated using a simple matching coefficient with DARwin software version 5.0.158 [77] and used to build a neighbor-joining tree. Because *Spontaneum* is a highly selfing species, the program InStruct [78] was used to infer population structure. InStruct is an extension of the approach used in STRUCTURE [79] and can specifically account for self-pollination and inbreeding. InStruct was run in mode v = 3 (infer population structure and individual selfing rates) for K = 1–10. For each K, 5 chains were run, with 200,000 MCMC iterations, a burn-in of 100,000 and a thinning interval of 10 steps. Results from independent chains were summarized using CLUMPP [80] and graphical representations of cluster assignments were rendered with DISTRUCT [81]. The *ad hoc* measure of change in likelihood between successive K values, ΔK [82] was calculated to identify the appropriate number of clusters. As recommended by Gao *et al*. [78], clustering results were compared with results obtained using STRUCTURE v. 2.3.3 [79] and Structure Harvester [83]. STRUCTURE was run with 5 independent runs for each value of K from 1 to 8, with a burn in period of 10^5^ followed by 10^5^ iterations.

### Description of environmental variation in Jordan

We used a procedure developed by Newman and Rissler [84] to delineate distinct environments within the study area. A habitat suitability model was generated for *Spontaneum* with MaxEnt version 3.3.3k [85]. Occurrence data in Jordan was downloaded from Genesys (https://www.genesys-pgr.org). Occurrences showing a geographical coordinate quality rank > 70 [86] were included. The 19 Bioclim layers for current climate conditions (1950–2000), at a resolution of 2.5 arc-minutes, were used. Ten thousand sites were sampled pseudo-randomly from the study area, in proportion to their suitability, as estimated in the habitat suitability model. The environmental data associated with each site was then extracted from all Bioclim layers. Following normalization of each environmental variable, the data set was subjected to k-means clustering such that each pseudo-randomly selected site was assigned to one of k classes. By coloring each site according to habitat, regions within the study area that had similar mean environmental conditions could be visualized.

### Association between genetic diversity, geography and environment

Correlations between allelic richness, InStruct clustering results and environmental data were tested using JMP 5.1 (SAS Institute, Cary, NC, USA). Means were compared using the Tukey-Kramer HSD test. Pearson product-moment and Spearman's Rho rank correlation coefficients were calculated. Isolation by distance (IBD) was estimated using R (http://www.r-project.org/). Geographic distances were calculated as straight-line distances with the GeographicDistanceMatrixGenerator version 1.2.3 [87] and log transformed. Genetic distances were calculated as F_ST_/(1- F_ST_) [88] and as population-wise allelic differences using the FPTEST [89]. Two-tailed Mantel tests were carried out with 10^5^ permutations. To test isolation by environment (IBE), environmental distances between sites were estimated. A principal coordinate analysis (PCO) was performed using data from all Bioclim variables and altitude. Environmental distance was then approximated as the simple Euclidean distance between points on the first principal coordinate axis, which accounted for 49% of the environmental variation across sampling sites. The multivariate measure of environmental distance represented a conservative approach aiming to avoid overfitting, as many of the Bioclim parameters covaried significantly. Two-tailed Mantel tests were carried out to estimate IBE. As environmental and geographical distances were significantly correlated, IBD and IBE were also tested using a partial Mantel test. In addition, correlation of the distance matrix calculated with FPTEST to individual Bioclim variables was examined using appropriate Holm-Bonferroni correction [90] to avoid type I error inherent in multiple comparisons.

## Results

### Genetic diversity

A total of 291 alleles were identified. Alleles per locus ranged from 3 to 22, with an average of 7.9. The mean number of alleles per locus averaged across sites was 2.8. PIC varied from 0.34 to 0.914 with a mean of 0.62. Allelic richness per locus varied from 2.2–9.1. All populations showed low observed heterozygosity (H_o_) ranging between 0–0.025. *Spontaneum* is a highly self-pollinating species and previous studies on *Spontaneum* reported similar levels of heterozygosity [66,91]. A total of 370 multi-locus genotypes were identified. Only three populations (5, 15, 18) showed a single multi-locus genotype twice. Allelic richness per population ranged from 1.4 to 3.3 with a mean of 2.63.

### Population differentiation among sites

**Fig 2:**
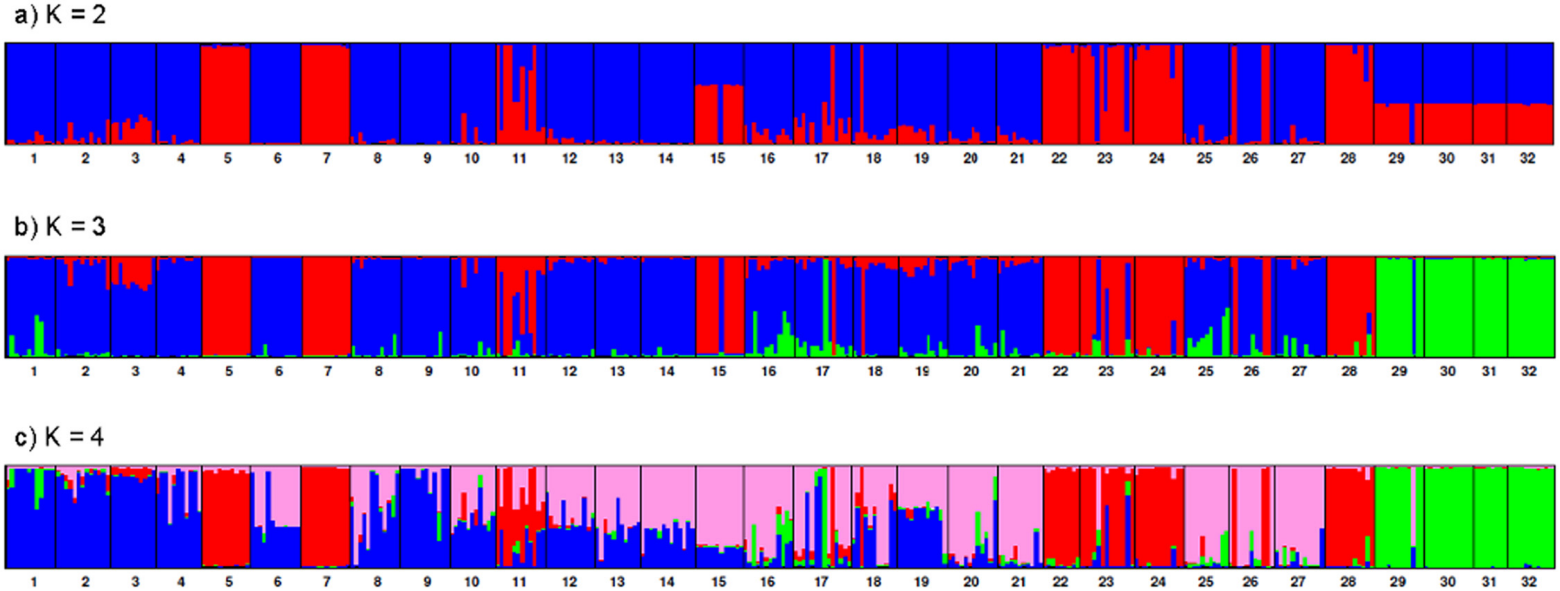
Assignment of individuals to genetic clusters identified by InStruct, for K = 2 to K = 4. Populations are sorted from left to right by decreasing latitude. Clusters are depicted in the following colours: cluster 1 = blue; cluster 2 = red; cluster 3 = green; cluster 4 = pink.

**Fig 3:**
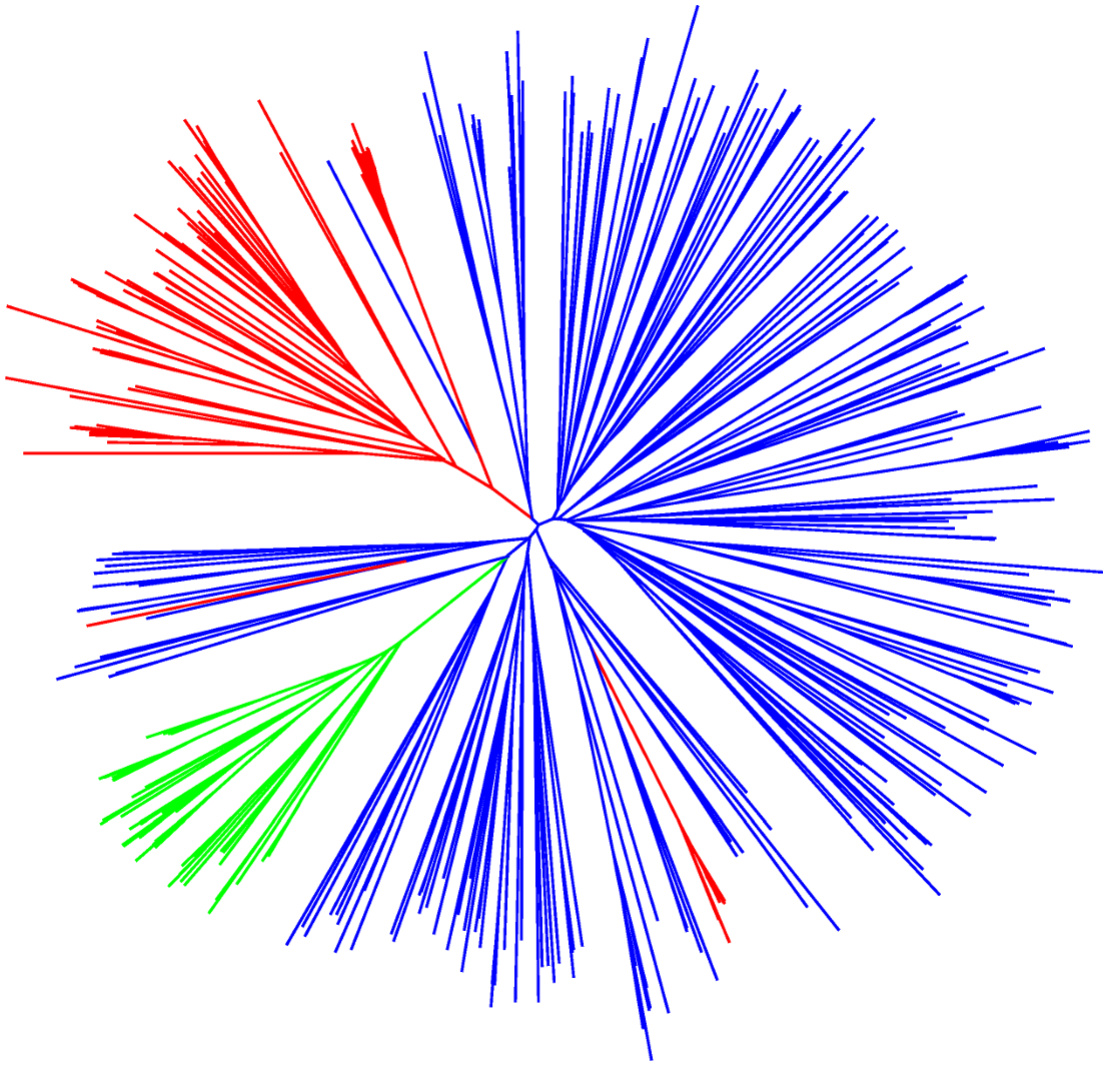
Neighbor-joining tree showing inter-individual genetic distances. Genetic clusters are depicted in the following colours: cluster 1 = blue; cluster 2 = red; cluster 3 = green.

Differentiation among populations measured as F_ST_ was 0.33, i.e. 33% of variation was distributed between populations and 67% within populations, similar to previous studies [57,59,92,93]. The ΔK method [82] applied to InStruct and STRUCTURE results suggested subdivision into three clusters. Fig 2 shows the individual assignment coefficients for K = 2 to K = 4. Partitioning into three genetic clusters produced one group of populations predominantly located in the northwestern part of the collecting area and a second cluster which showed a longitudinal extension from the northeast southwards. A small third cluster was geographically separated in the southern part of the collecting area. The geographical distribution of the three clusters is shown in Fig 1. The InStruct assignment was compatible with the neighbor-joining tree based on inter-individual genetic distances (Fig 3). Assignment coefficients (q) varied across the study area. The average assignment coefficient of individuals to cluster 1 was significantly lower (q = 0.902; p = <0.0001) than those of cluster 2 (q = 0.966) and cluster 3 (q = 0.99). The assignment coefficient was inversely correlated with latitude (Pearson coefficient: r = -0.17; p = 0.001; Spearman’s rank coefficient: r = -0.204; p<0.0001) and positively correlated with altitude (Pearson coefficient: r = 0.166; p = 0.0013; Spearman’s rank coefficient: r = 0.235; p<0.0001) indicating that the level of admixture was higher in the north. While there were 10 populations whose respective individuals were all strongly assigned to the same genetic cluster (q ≥ 0.8), the remaining populations contained some individuals either strongly assigned to a different cluster (physical admixture), and/or some genetically admixed individuals (0.49 < q < 0.8) (Table 4). Eight populations were physically admixed, with 1–6 individuals assigned to a different cluster. 18 populations contained 1–9 genetically admixed individuals (four of these populations were also physically admixed). In populations assigned to cluster 3, only the population in site 29 showed physical admixture (one individual assigned to cluster 1), no genetic admixture was identified in any of the four populations of cluster 3. All other physical admixture stems from individuals either assigned to cluster 1 but growing within a site assigned to cluster 2 or vice versa. 88% of the 43 genetically admixed individuals belong to populations assigned to cluster 1, the remaining to cluster 2.

**Table 4:** Genetic and physical admixture.

	Physical admixture		Genetic admixture	
Cluster	No. of populations	No. of individuals	No. of populations	No. of individuals
1	4	11	14	38
2	3	5	4	5
3	1	1	0	0

### Association between genetic diversity, geography and environment

**Fig 4:**
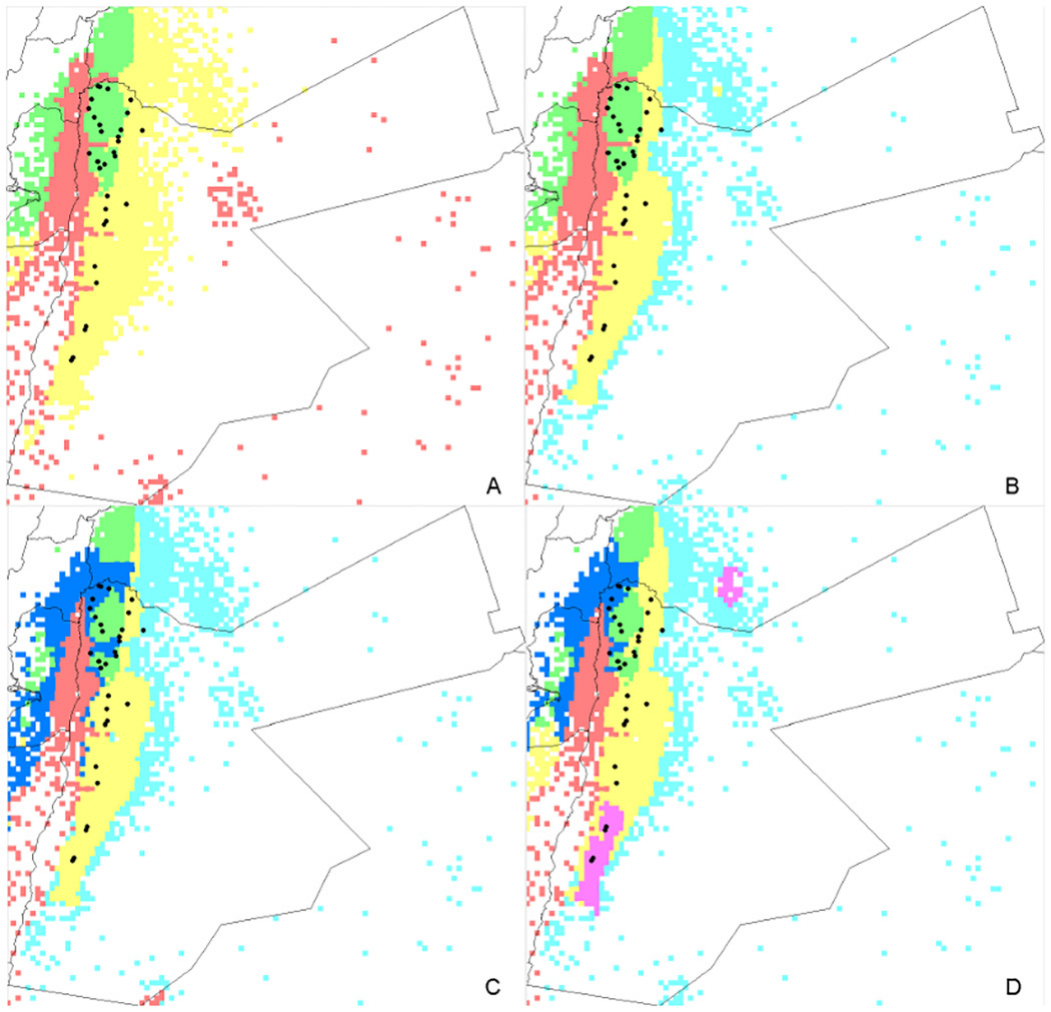
Habitat types in Jordan identified through k-means clustering. Black dots represent the *Spontaneum* collecting sites.

K-means clustering was used to delineate different environments that might be inhabited by *Spontaneum* in Jordan. Regions of the study area with distinct environmental conditions are depicted in Fig 4. They are predominantly arranged as north-south stripes corresponding to the three main topographical regions described by Al-Eisawi [94] (rift valley along the western border; mountain range extending from the north in Irbid to the south in Ras An-Naqab, and the eastern desert). Although the sampling scheme also followed a north-south transect, populations were sampled from the majority of the distinct environments identified (Fig 4). The geographical distribution of the genetic clusters did not match the geographical distribution of these environmental partitions.

When comparing populations collected from nature reserves and those collected from ruderal habitats, roadsides or field margins, no significant differences in genetic diversity measures were found. No physical admixture was detected in populations collected in reserves, while they do show genetic admixture. Plants collected in reserves were significantly smaller (37.0 cm) than those collected from ruderal areas or field margins (72.6 cm; p = 0.0047). Also population size observed in reserves was significantly smaller (p = 0.0112, Tukey-Kramer HSD test; based on observed size of all wild populations sampled during the 2012 barley collecting mission). The average habitat suitability, according to the habitat suitability model, was significantly lower in reserves than in the other sites (p = 0.0289).

Average values of geographical, geophysical and Bioclim variables, allelic richness, and selfing rates are compared among clusters in Table 5. Average longitude and Bioclim 6 were the only variables that were significantly different between all three clusters. Cluster 3 collecting sites were significantly different for several variables including: higher elevation, lower latitude, lower values for temperature-related Bioclim variables 1, 5, 8, 9, 10, 11 (these Bioclim variables are highly correlated, r>0.8) and lower selfing rates. Cluster 1 showed significantly higher allelic richness and higher values for Bioclim 13. No significant differences were found for habitat type, aspect, soil type and Bioclim 2, 3, 7, 15, 16 and 19. Bioclim 14, 17 and 18 were zero at all sites. Allelic richness of loci and of populations was weakly correlated with latitude (loci: Spearman’s rank coefficient r = 0.079, p = 0.0065; Pearson’s coefficient r = 0.147, p<0.0001; populations: Pearson’s coefficient r = 0.36, p = 0.0432).

**Table 5:** Comparison of average values for geographical, geophysical and Bioclim variables, allelic richness and selfing rate at collecting sites among genetic clusters.

Variable	Cluster 1			Cluster 2			Cluster 3		
	Level	Mean	p	Level	Mean	p	Level	Mean	p
Allelic richness	A	2.91		B	2.25	<0.0001	B	2.0	<0.0001
Selfing rate	A	0.831	0.0294	A	0.833	0.0220	B	0.0813	
Altitude (m)	B	564	<0.0001	B	749	0.0005	A	1521	
Latitude	A	32.195	<0.0001	A	31.968	<0.0001	B	30.539	
Longitude	A	35.76	0.0015 (1–3)	B	35.9	0.0038 (2–1)	C	35.56	<0.0001 (3–2)
Aspect	A	233.99	ns	A	185.07	ns	A	199.04	ns
Slope	A	3.54	0.0030	B	0.99		A	4.81	0.0024
Bioclim 1	A	18.03	<0.0001	A	16.67	0.0122	B	13.78	
Bioclim 2	A	11.68	ns	A	12.21	ns	A	11.60	ns
Bioclim 3	A	42.94	ns	A	44.10	ns	A	42.82	ns
Bioclim 4	A	625.73	0.0151	AB	620.55		B	598.47	
Bioclim 5	A	32.07	0.0005	A	30.94	0.0249	B	27.96	
Bioclim 6	A	4.9	<0.0001 (1–3)	B	3.25	0.03 (2–1)	C	0.86	0.0332 (3–2)
Bioclim 7	A	27.17	ns	A	27.69	ns	A	27.1	ns
Bioclim 8	A	10.1	0.0002	A	8.68	0.0287	B	6.06	
Bioclim 9	A	25.0	<0.0001	A	23.59	0.0145	B	20.65	
Bioclim 10	A	25.04	<0.0001	A	23.63	0.0130	B	20.65	
Bioclim 11	A	10.1	0.0002	A	8.68	0.0287	B	6.06	
Bioclim 12	A	388.0	0.0448 (1–3)	AB	323.88		B	289.75	
Bioclim 13	A	92.1		B	73.75	0.0314	B	67.0	0.0238
Bioclim 15	A	113.44	ns	A	113.02	ns	A	115.12	ns
Bioclim 16	A	250.1	ns	A	206.88	ns	A	187.0	ns
Bioclim 19	A	250.1	ns	A	206.88	ns	A	187.0	ns

Bioclim 1- Bioclim 19 = Bioclimatic variables as per definition on http://www.worldclim.org/bioclim (see Table 2); ns = non-significant; levels marked with different letters indicate significant difference among cluster averages (p<0.05) based on the Tukey HSD test. Numbers in brackets after p values indicate the two clusters being compared.

Genetic and geographical distance were significantly correlated (F_ST_ based distance: r = 0.3, p = 0.0003; FPTEST based distance: r = 0.2, p = 0.02), suggesting isolation by distance, when analyzed over all 32 populations, while the Mantel tests for isolation by environment were not significant. Environmental and geographic distance were strongly correlated (r = 0.4, p = 0.0001), indicating possible confounding effects. These were accounted for using a partial Mantel test which confirmed significant IBD among all studied populations (r = 0.25, p = 0.004), but did not find significant IBE (S1 Resource). Several climate variables were found to be different in cluster 3 compared to cluster 1 and 2 (Table 5). Cluster 3 was furthermore geographically separated from clusters 1 and 2, which are themselves partly overlapping. The correlation analyses were therefore repeated for populations belonging to clusters 1 and 2 only. No significant IBD or IBE was found between cluster 1 and 2, and neither were environmental and geographic distances significantly correlated. No significant correlations existed between single Bioclim variables and distance matrix calculated with FPTEST (S1 Resource).

## Discussion

The present study examined the current geography of genetic structure and its correlation with landscape scale climatic and spatial variation in *Spontaneum* populations in Jordan. Correlation analyses showed large scale IBD across the study area but did not reveal a correspondence between climate and genetic structure. Analysis of population structure suggested that the 32 *Spontaneum* populations could be divided into three major, genetically differentiated clusters (Fig 1). Genetic diversity was concentrated in the northern part of the study area, across a range of environments, where populations are characterized by physical and genetic admixture, and high allelic richness. Allelic richness and admixture decrease towards the south; the southernmost populations are not admixed, exhibit low allelic richness and contain physically smaller plants.

### Genetic structure is not correlated with climatic variation inferred from global layers

Three genetic clusters were distributed along a longitudinal gradient in the North (clusters 1 and 2), with a distinct cluster (cluster 3) in the South. The study area was characterized by a longitudinal distribution of distinct habitat types as shown in Fig 4, of which the central mountain range was the most variable. At the large scale across the entire study area, where geographical and environmental distances were strongly correlated, significant IBD implied that physical distance was important for genetic differentiation among populations, but environmental variation was found to have no effect. Results were different at a slightly smaller scale, across the central and northern part of the study area, where clusters 1 and 2 spread across an environmentally heterogeneous landscape. Here, geographical and environmental distances were both uncorrelated with genetic distance either measured by F_ST_ or by population-wise allelic differences.

*Spontaneum* prefers disturbed, human-made or influenced habitats [20,22], sympatric with its domesticate [95–98]. These habitats favor anthropogenic movement of material—inclusion and transport with cultivated barley seed lots or hitchhiking on livestock fur or human clothing—which interferes with natural diffusion and selection processes. This may alter the expected distribution of genetic diversity across the landscape and lead to weak or nonexistent correlations between ecogeographical and genetic diversity as found in our study. Natural dispersal and selection processes may not have been the principle force shaping genetic structure in some regions of Jordan.

*Spontaneum* is a highly self-pollinating species. In self-pollinating species much genetic diversity is distributed among populations rather than within populations, population to population variation is greater than in out-crossing species and the genetic structure is more variable [99]. Given their low gene flow and very localized gene transfer, genetic structure has been found at local scale [63,100,101]. This local variation is unlikely to be detected by globally available layers commonly used to represent landscape scale spatial and climatic variation.

Global climate data such as the Bioclim layers provided by WorldClim climatic data are used in a range of studies and applications [11,19,67–69,85,86,102], and the inherent assumption is that they are robust proxies for genetic data, which is often not available. Our results suggest that there may be some limitations on this assumption. Our study did not find a correlation between climate, as represented by commonly used global, interpolated data layers, and genetic structure for *Spontaneum*. Thus global climatic data would not be especially useful for predicting existing genetic diversity in Jordan. A ruderal habitat preference and high self-pollination might explain why the general expectation of tight correlation between genetic and ecogeographical diversity does not hold. If collecting and conservation actions are designed without previous knowledge of genetic structure, it will be important to consider species biology and habitat preferences when using ecogeographical diversity to predict genetic diversity.

### Sampling and monitoring genetic diversity within *Spontaneum* populations

All *Spontaneum* populations sampled here, irrespective of cluster assignment, contained many unique multi-locus genotypes. Only three populations showed a single multi-locus genotype twice, and no multi-locus genotype was repeated among populations. Allelic richness, which is a good metric to assess and monitor genetic diversity [103], increased significantly towards the northern part of the study area. Here, populations were also characterized by admixture. More than half of the populations in clusters 1 and 2 showed considerable genetic admixture as well as physical admixture, a characteristic that was also found by Hübner *et al*. [66] in Israel. Hübner *et al*. [93] observed a fairly high rate of gene flow in *Spontaneum* attributed to sporadic outcrossing events [104] and gene flow through seed dispersal. These mechanisms likely contribute to physical and genetic admixture in Jordan as well.

Due to the reduced level of diversity expected within populations of highly selfing species, germplasm collections are often limited to a few samples per population. The heterogeneity found within populations in this study cautions against such sampling strategies. Modeling studies have shown that collections of highly selfing species need substantially more samples than are commonly recommended to capture existing diversity [105]. The distribution of genetic structure we have described for *Spontaneum* in Jordan prescribes further collecting and monitoring in the northern part of the country, in particular the area occupied by cluster 1.

### *Ex situ* and *in situ* conservation of *Spontaneum*

Natural populations of *Spontaneum* have been reported to harbour large neutral genetic diversity, and also show considerable diversity in disease resistance and quantitative traits of agronomic importance [45,106–108]. Despite evidence of high genetic, adaptive and quantitative diversity in Jordanian *Spontaneum* populations, the number of *ex situ* barley accessions from Jordan in global collections is lower than those from neighboring countries. Although in general the number of *Spontaneum* accessions in *ex situ* collections seems relatively high compared with other CWR samples in genebanks, they are derived from a limited number of populations [109]. Maxted and Kell [24] suggest that, although *Spontaneum* is widespread and locally common [110], individual populations might contain important adaptive traits, thus populations should be actively conserved throughout the geographical range. Vincent *et al*. [111] identified Jordan as one of the countries where wild *Hordeum* should be conserved and suggested the establishment of a network of several reserves in the Israel/Jordan region to more effectively conserve the genetic diversity of wild *Hordeum*. These assessments describe the obvious need to promote *in situ* conservation of *Spontaneum* in Jordan and to enlarge *ex situ* collections. Our description of the distribution of genetic diversity across the Jordanian landscape provides a tool to evaluate the propriety of existing *in situ* conservation activities and supports the application of proper sampling techniques for future *ex situ* acquisitions.

## Supporting Information

S1 Resource Correlation analyses between genetic, geographic and environmental data. (PDF)

S1 Table Microsatellite data for 373 *Spontaneum* individuals. (PDF)

S2 Table Ecogeographical and genetic data for 32 *Spontaneum* populations collected in Jordan in 2012. (PDF)
