# Supplementary material for: Geography of Genetic Structure in Barley Wild Relative Hordeum vulgare subsp. spontaneum in Jordan
Source: PLoS One. 2016 Aug 11;11(8):e0160745. doi: 10.1371/journal.pone.0160745 (PMC4981475; doi:10.1371/journal.pone.0160745)
Supplement: S1 Resource — (PDF) [file pone.0160745.s001.pdf]

S1 Resource. Correlation analyses between genetic, geographic and environmental data
Geography of genetic structure in barley wild relative *Hordeum vulgare* subsp. *spontaneum* in Jordan
PloS ONE
Thormann I. et al.

Two-tailed Mantel test results for isolation by distance and by environment

Type of distances between populations	Description
GenDist(Fst/(1-Fst))	Genetic distances based on Fst/(1-Fst)
GenDist(FPTest)	Genetic distances based on allele counts calculated with FPTEST [89]
GenDist(AllelRich)	Genetic distance based on allelic richness
LnGeoDist	Logarithm of geographic distances in km
EnvDist	Environmental distances based on Bioclim variables PCoA

All 32 populations

r/p	LnGeoDist	EnvDist	GenDist (FPTest)
GenDist(Fst/(1-Fst))	0.29486/ 0.00042	0.16197/ 0.17396	0.39238/ 0.00038
LnGeoDist	--	0.40156/ 0.00001	0.19402/ 0.02009
EnvDist		--	0.07315/ 0.45288

Cluster 1 and 2 (28 populations)

r/p	LnGeoDist	EnvDist	GenDist (FPTest)
GenDist(Fst/(1-Fst))	0.06014/ 0.55819	-0.07475/ 0.56571	0.55219/ 0.00047
LnGeoDist	--	0.07321/ 0.34933	-0.03102/ 0.73936
EnvDist		--	-0.1322601/ 0.21046

Partial Mantel test results between environmental and geographic distances
EnvDist/LnGeoDist

All 32 populations
r=0.375
p=0.0001

Cluster 1 and 2 (28 populations)
r=0.078
p=0.316

Pairwise distances among populations

GenDist (FPTest))	GenDist (Fst/(1-Fst))	LnGeoDist	EnvDist
15	0.112896	0.412110	0.255073
5	0.305781	2.099244	1.013590
12	0.185880	2.590017	0.180762
53	0.646681	3.360375	0.933676
10	0.337385	3.110399	0.132297
32	0.519436	3.528417	1.208091
21	0.162732	3.389462	1.262319
2	0.292040	3.593194	1.570917
7	0.198974	3.801091	1.744536
1	0.314015	3.990834	1.339027
4	0.191638	3.749033	1.671406
14	0.217124	3.913422	1.215445
6	0.237751	3.987872	0.745869
34	0.598911	4.147727	1.058673
13	0.279402	4.145830	0.037163
20	0.250887	4.201553	1.157584
0	0.219599	4.254335	1.896805
4	0.229597	4.258870	1.946168
25	0.209620	4.293878	1.646949
18	0.239109	4.342506	1.439009
13	0.342464	4.634826	1.568469
10	0.321274	4.725262	1.607242
11	0.383812	4.745193	1.513266
2	0.314361	4.841190	1.095632
8	0.242681	4.863372	1.270136
6	0.265652	5.127292	1.357421
8	0.353595	5.215370	1.798254
12	0.403876	5.417033	2.572677
36	0.486062	5.429828	2.104698
38	0.480534	5.539929	2.587051
29	0.421809	5.550359	2.640546
20	0.218476	1.897620	0.758517
3	0.128407	2.588516	0.074312
68	0.436611	3.306520	0.678603
25	0.237423	3.102791	0.122776
47	0.332082	3.486151	0.953018
6	0.100795	3.371425	1.007246
13	0.243710	3.573469	1.315844
8	0.138353	3.774828	1.489463
14	0.193114	3.964805	1.083954
11	0.143596	3.730981	1.416333
1	0.155763	3.891820	0.960372
9	0.179381	3.968592	0.490796
49	0.440156	4.132764	0.803600
2	0.196123	4.137564	0.217910
5	0.192890	4.187379	0.902511
15	0.132527	4.244774	1.641732
11	0.177412	4.249352	1.691095
10	0.128600	4.283311	1.391876
3	0.164703	4.333493	1.183936
28	0.218747	4.627225	1.313396
5	0.251873	4.716890	1.352169
26	0.242390	4.738652	1.258193
13	0.202609	4.835170	0.840560
7	0.145636	4.857640	1.015063
9	0.158919	5.123309	1.102347
23	0.215894	5.211724	1.543181
27	0.328531	5.414232	2.317604
51	0.331773	5.427106	1.849625
53	0.405709	5.537649	2.331978

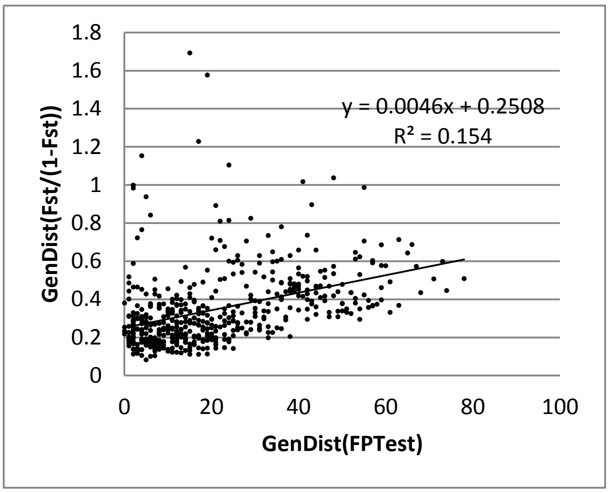

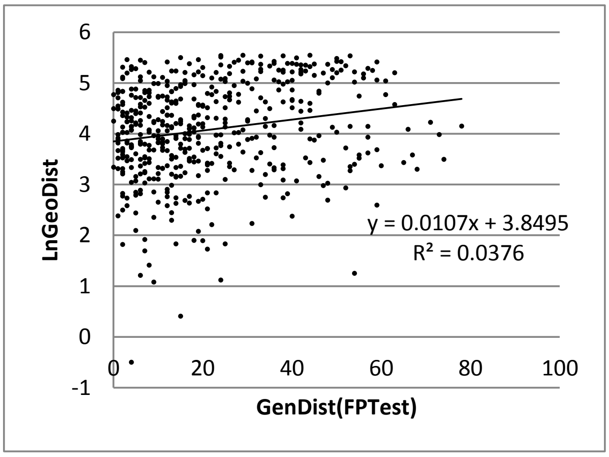

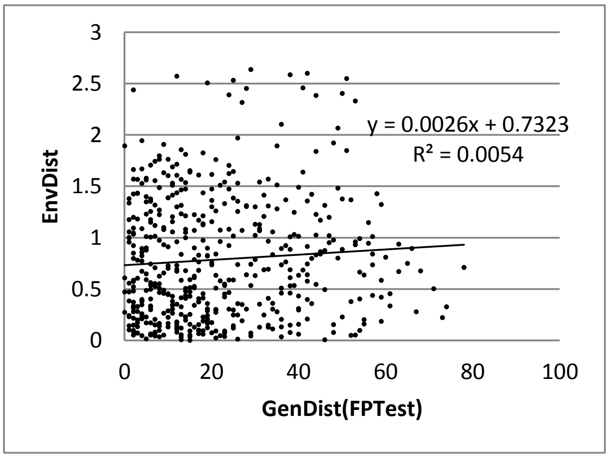

44	0.320847	5.548142	2.385473
17	0.265777	2.770086	0.832829
48	1.038345	3.030134	0.079914
5	0.428217	3.163786	0.881293
27	0.541901	3.296577	0.194501
26	0.273486	3.355502	0.248729
7	0.450734	3.528417	0.557327
12	0.283818	3.676301	0.730946
6	0.363018	3.853546	0.325437
9	0.309518	3.685373	0.657816
19	0.317500	3.815071	0.201855
11	0.335960	3.900153	0.267721
29	0.825128	4.083621	0.045083
18	0.329936	4.122284	0.976427
25	0.334935	4.140273	0.143994
5	0.319254	4.220390	0.883215
9	0.377168	4.224203	0.932578
30	0.311096	4.252345	0.633359
23	0.350055	4.308515	0.425419
8	0.374385	4.604170	0.554879
15	0.429661	4.688040	0.593652
6	0.377842	4.718677	0.499676
7	0.428582	4.816970	0.082042
13	0.330247	4.840479	0.256546
11	0.401033	5.111928	0.343831
3	0.368040	5.200925	0.784664
7	0.507065	5.406319	1.559087
31	0.630672	5.419428	1.091108
33	0.735757	5.531530	1.573461
24	0.601235	5.542204	1.626956
65	0.643872	3.436243	0.752915
22	0.307241	2.218116	0.048465
44	0.435322	3.439135	1.027329
9	0.144465	2.857619	1.081558
10	0.259935	3.217275	1.390155
5	0.170613	3.609566	1.563775
11	0.250307	3.903184	1.158265
8	0.174545	3.439135	1.490645
2	0.178449	3.716008	1.034683
6	0.188295	3.797734	0.565108
46	0.516330	3.968214	0.877911
1	0.226202	3.922963	0.143599
8	0.223466	4.032292	0.976822
12	0.214385	4.066288	1.716043
8	0.229077	4.072610	1.765407
13	0.147978	4.122122	1.466188
6	0.167273	4.173772	1.258248
25	0.289066	4.514917	1.387708
2	0.291628	4.625953	1.426481
23	0.326650	4.637153	1.332505
10	0.224075	4.743366	0.914871
4	0.192055	4.766949	1.089374
6	0.178389	5.051969	1.176659
20	0.294951	5.146855	1.617493
24	0.367198	5.360400	2.391915
48	0.419779	5.373842	1.923937
50	0.478920	5.489268	2.406289
41	0.416551	5.500156	2.459785
43	0.897667	3.530470	0.801379
21	0.892651	2.523326	0.274415
74	0.446256	3.503453	0.328643
55	0.987396	3.526066	0.637240
60	0.576942	3.376221	0.810860

54	0.624508	3.401531	0.405350
57	0.592793	3.627802	0.737730
67	0.573300	3.586016	0.281768
59	0.686999	3.691626	0.187807
19	1.578266	3.930845	0.124997
66	0.688521	4.091841	0.896514
73	0.600007	3.989354	0.223907
53	0.611370	4.149622	0.963128
57	0.603556	4.151040	1.012492
78	0.508872	4.155440	0.713273
71	0.508261	4.229895	0.505333
40	0.565401	4.520157	0.634793
63	0.714679	4.582209	0.673566
42	0.737728	4.644102	0.579590
55	0.706803	4.747190	0.161956
61	0.492578	4.774238	0.336459
59	0.578542	5.064302	0.423744
45	0.547143	5.155601	0.864578
41	1.019039	5.371196	1.639001
17	1.228899	5.385091	1.171022
15	1.693544	5.502849	1.653374
24	1.106139	5.514154	1.706870
22	0.710059	3.423611	1.075794
31	0.238382	2.237513	1.130022
12	0.422865	2.855320	1.438620
17	0.290189	3.467609	1.612239
11	0.347185	3.843316	1.206730
14	0.282773	3.151453	1.539109
24	0.277171	3.555919	1.083148
16	0.302573	3.640477	0.613572
24	0.815106	3.815512	0.926376
23	0.288378	3.726898	0.095134
30	0.353987	3.888959	1.025287
10	0.350407	3.904595	1.764508
14	0.376412	3.912623	1.813871
35	0.250851	3.975936	1.514652
28	0.281074	4.030872	1.306712
3	0.464973	4.416790	1.436172
20	0.410427	4.544677	1.474945
1	0.518709	4.549657	1.380969
12	0.374230	4.664759	0.963336
18	0.284852	4.689879	1.137839
16	0.303671	4.993218	1.225124
2	0.465430	5.093627	1.665957
2	0.589135	5.317188	2.440380
26	0.630886	5.331172	1.972401
28	0.706833	5.451081	2.454754
19	0.553426	5.462348	2.508249
53	0.380936	3.276390	0.054228
34	0.650085	3.204777	0.362826
39	0.463307	2.823163	0.536445
33	0.257404	3.002708	0.130936
36	0.413163	3.294725	0.463316
46	0.392890	3.163786	0.007354
38	0.448510	3.319626	0.462222
2	0.984609	3.650658	0.149418
45	0.410207	3.880326	1.170928
52	0.326468	3.726898	0.050507
32	0.339956	3.937886	0.688714
36	0.489225	3.938860	0.738077
57	0.369104	3.940027	0.438858
50	0.379393	4.032824	0.230918
19	0.184741	4.374246	0.360379

42	0.399826	4.448399	0.399151
21	0.199197	4.516339	0.305176
34	0.545019	4.632883	0.112458
40	0.344098	4.663156	0.062045
38	0.442370	4.982373	0.149330
24	0.178248	5.081094	0.590164
20	0.721700	5.311579	1.364586
4	0.766605	5.326322	0.896608
6	0.843201	5.450738	1.378960
3	0.723290	5.462645	1.432455
19	0.247097	2.081938	0.308598
14	0.132012	3.170945	0.482217
20	0.188256	3.677819	0.076708
17	0.145924	2.640485	0.409087
7	0.137526	3.258481	0.046875
15	0.165035	3.364188	0.516450
55	0.424193	3.584629	0.203646
8	0.185812	3.512441	1.225157
1	0.181138	3.676301	0.104735
21	0.176008	3.712596	0.634485
17	0.196388	3.721347	0.683849
4	0.106520	3.790759	0.384630
3	0.123626	3.861992	0.176690
34	0.226033	4.303524	0.306150
11	0.289834	4.441356	0.344923
32	0.298496	4.452485	0.250947
19	0.189926	4.578929	0.166687
13	0.119474	4.606869	0.007816
15	0.147115	4.934762	0.095101
29	0.241526	5.040776	0.535935
33	0.336841	5.276174	1.310358
57	0.365294	5.290839	0.842379
59	0.398236	5.416189	1.324731
50	0.340403	5.427985	1.378227
5	0.283643	2.851284	0.173620
1	0.402200	3.523120	0.231890
2	0.253633	1.824549	0.100490
12	0.256339	2.910174	0.355472
4	0.310094	3.044999	0.825047
36	0.781455	3.335058	0.512244
11	0.327731	3.322875	1.533754
18	0.255527	3.451574	0.413333
2	0.273236	3.530177	0.325888
2	0.330444	3.538928	0.375252
23	0.262907	3.609024	0.076032
16	0.273788	3.702782	0.131907
15	0.461844	4.200055	0.002447
8	0.418567	4.345622	0.036325
13	0.494187	4.365389	0.057650
0	0.381795	4.502805	0.475284
6	0.302747	4.533674	0.300781
4	0.347576	4.884770	0.213496
10	0.455711	4.995657	0.227338
14	0.569293	5.241641	1.001760
38	0.630108	5.256974	0.533782
40	0.659008	5.387152	1.016134
31	0.590548	5.399384	1.069630
6	0.273285	2.823757	0.405510
3	0.143472	2.737609	0.073130
7	0.128475	1.921325	0.529092
1	0.168959	2.385086	0.998667
41	0.521407	3.076851	0.685863
6	0.202593	3.503453	1.707374

13	0.219317	3.211247	0.586952
7	0.163981	3.550766	0.152268
3	0.245133	3.550766	0.201632
18	0.141708	3.544432	0.097587
11	0.197275	3.680847	0.305527
20	0.265644	4.138202	0.176067
3	0.301046	4.243339	0.137294
18	0.327299	4.315353	0.231270
5	0.221061	4.456786	0.648904
1	0.189445	4.492449	0.474401
1	0.203112	4.861129	0.387116
15	0.274972	4.972379	0.053718
19	0.353499	5.226015	0.828141
43	0.392345	5.242012	0.360162
45	0.471075	5.376435	0.842514
36	0.405709	5.389209	0.896010
3	0.243203	3.472898	0.332380
13	0.218522	2.989211	0.123582
5	0.272786	3.074081	0.593157
35	0.601805	3.409166	0.280354
12	0.233221	3.851211	1.301864
19	0.207765	3.466673	0.181443
1	0.258212	3.824502	0.557778
3	0.326455	3.820346	0.607141
24	0.191129	3.772301	0.307922
17	0.209762	3.890595	0.099983
14	0.189685	4.207971	0.229443
9	0.239833	4.250065	0.268215
12	0.223838	4.367674	0.174240
1	0.310370	4.495020	0.243394
7	0.177674	4.531201	0.068891
5	0.272740	4.886054	0.018394
9	0.174118	4.991249	0.459228
13	0.399016	5.240847	1.233650
37	0.441302	5.256870	0.765672
39	0.441559	5.390897	1.248024
30	0.392767	5.403758	1.301519
10	0.138760	2.656055	0.455962
2	0.139301	2.775086	0.925537
38	0.509158	3.093313	0.612734
9	0.210907	3.116178	1.634244
16	0.177529	3.238286	0.513823
4	0.175552	3.339322	0.225398
0	0.254147	3.348851	0.274762
21	0.143660	3.426540	0.024457
14	0.174889	3.541829	0.232397
17	0.264700	4.103304	0.102937
6	0.267445	4.262116	0.064164
15	0.288979	4.284414	0.158140
2	0.262225	4.432482	0.575774
4	0.183660	4.465908	0.401271
2	0.220148	4.838185	0.313986
12	0.274439	4.953924	0.126848
16	0.387235	5.209541	0.901271
40	0.469927	5.225424	0.433292
42	0.497210	5.359789	0.915644
33	0.441176	5.372404	0.969140
8	0.150516	1.415853	0.469575
48	0.534804	2.700018	0.156772
1	0.185388	3.318540	1.178282
6	0.159957	2.893700	0.057861
14	0.176708	3.341801	0.681360
10	0.242039	3.340739	0.730724

11	0.123060	3.325396	0.431504
4	0.136189	3.494080	0.223565
27	0.249824	4.024101	0.353025
4	0.294854	4.148517	0.391798
25	0.313309	4.220830	0.297822
12	0.194561	4.375757	0.119812
6	0.156183	4.414010	0.054691
8	0.167516	4.807458	0.141976
22	0.255046	4.924714	0.582810
26	0.364066	5.189172	1.357232
50	0.377307	5.205709	0.889254
52	0.435817	5.344628	1.371606
43	0.376024	5.357812	1.425102
40	0.533265	2.384165	0.312804
7	0.178615	3.235536	0.708707
14	0.193049	2.636196	0.411715
6	0.196629	3.216473	1.150935
2	0.285418	3.213260	1.200299
19	0.144833	3.178054	0.901080
12	0.175295	3.374853	0.693140
19	0.275664	3.948162	0.822600
4	0.291680	4.081935	0.861373
17	0.353072	4.158727	0.767397
4	0.272006	4.322675	0.349763
2	0.177309	4.363099	0.524266
0	0.233806	4.773393	0.611551
14	0.306055	4.894401	1.052385
18	0.365124	5.165985	1.826808
42	0.449662	5.182963	1.358829
44	0.464931	5.324911	1.841181
35	0.428772	5.338355	1.894677
47	0.469372	2.985177	1.021510
54	0.531132	1.255616	0.098911
34	0.501365	2.756840	0.838132
38	0.453078	2.740840	0.887495
59	0.462380	2.599722	0.588276
52	0.435394	2.936513	0.380336
21	0.661518	3.715765	0.509797
44	0.659555	3.901366	0.548569
23	0.678268	3.975373	0.454594
36	0.610152	4.169916	0.036960
42	0.430007	4.216415	0.211463
40	0.418952	4.678235	0.298748
26	0.606002	4.810964	0.739581
22	0.810309	5.102850	1.514004
2	1.000827	5.120804	1.046025
4	1.154016	5.271049	1.528378
5	0.938699	5.285181	1.581873
7	0.190930	3.037833	1.120421
13	0.241958	2.302585	1.859642
9	0.250441	2.358965	1.909006
12	0.160344	2.766948	1.609786
5	0.176269	2.794228	1.401847
26	0.278723	3.749739	1.531307
3	0.298013	4.029273	1.570080
24	0.305227	3.984344	1.476104
11	0.212438	4.176692	1.058470
5	0.159889	4.214791	1.232973
7	0.185055	4.662401	1.320258
21	0.258266	4.799667	1.761092
25	0.286795	5.089200	2.535514
49	0.309110	5.106794	2.067536
51	0.350044	5.255097	2.549888

42	0.296099	5.268889	2.603384
20	0.206125	2.714695	0.739221
16	0.208205	2.689886	0.788584
5	0.160551	2.443216	0.489365
2	0.167898	2.830858	0.281426
33	0.227472	3.634423	0.410886
10	0.248812	3.827989	0.449658
31	0.248276	3.912623	0.355683
18	0.242274	4.118061	0.061951
12	0.172930	4.167285	0.112552
14	0.218981	4.647846	0.199837
28	0.224867	4.784069	0.640671
32	0.307604	5.083018	1.415093
56	0.358369	5.101390	0.947115
58	0.374742	5.254574	1.429467
49	0.338992	5.268992	1.482962
4	0.241648	-0.494296	0.049364
25	0.141956	1.835776	0.249856
18	0.178877	1.906575	0.457795
13	0.211998	3.505858	0.328335
10	0.261578	3.836006	0.289563
11	0.244083	3.806218	0.383538
2	0.223346	4.034418	0.801172
8	0.167065	4.081259	0.626669
6	0.175945	4.586497	0.539384
8	0.215511	4.733387	0.098550
12	0.369476	5.042457	0.675872
36	0.399315	5.061138	0.207894
38	0.484401	5.217541	0.690246
29	0.402712	5.232124	0.743742
21	0.195619	1.733424	0.299219
14	0.212413	1.838961	0.507159
17	0.340202	3.492560	0.377699
6	0.371022	3.824284	0.338926
15	0.409539	3.797285	0.432902
2	0.285542	4.027492	0.850536
4	0.217815	4.074992	0.676033
2	0.206220	4.583333	0.588748
12	0.313781	4.730480	0.147914
16	0.395869	5.040582	0.626509
40	0.453149	5.059298	0.158530
42	0.520901	5.216076	0.640882
33	0.377326	5.230681	0.694378
7	0.103926	1.697449	0.207940
38	0.205680	3.393165	0.078480
15	0.233387	3.721831	0.039707
36	0.246959	3.733136	0.133683
23	0.146580	3.977623	0.551317
17	0.112003	4.030339	0.376814
19	0.113833	4.561741	0.289529
33	0.198506	4.711061	0.151305
37	0.308091	5.027886	0.925728
61	0.331458	5.047096	0.457749
63	0.369531	5.206805	0.940102
54	0.295929	5.221706	0.993597
31	0.264419	3.280535	0.129460
8	0.273697	3.685875	0.168233
29	0.279604	3.645450	0.074257
16	0.162589	3.909018	0.343377
10	0.128403	3.962906	0.168874
12	0.122270	4.518631	0.081589
26	0.241081	4.674790	0.359245
30	0.330049	5.000518	1.133668

54	0.350490	5.020057	0.665689
56	0.394634	5.183075	1.148041
47	0.309634	5.198221	1.201537
23	0.190310	2.843164	0.038773
2	0.113699	2.499795	0.055203
15	0.336798	3.165475	0.472837
21	0.192383	3.285038	0.298334
19	0.278038	4.197052	0.211049
5	0.082353	4.403788	0.229785
1	0.444484	4.815350	1.004208
23	0.508990	4.839293	0.536229
25	0.595166	5.034091	1.018581
16	0.478652	5.052033	1.072077
21	0.235141	2.839663	0.093976
8	0.359640	3.118392	0.511610
2	0.238278	3.253084	0.337107
4	0.308703	4.146463	0.249822
18	0.188063	4.348341	0.191012
22	0.504972	4.778535	0.965435
46	0.554269	4.804021	0.497456
48	0.573664	5.007965	0.979808
39	0.481325	5.026968	1.033304
13	0.372421	2.450143	0.417634
19	0.221445	2.676903	0.243131
17	0.325242	3.995077	0.155846
3	0.116508	4.242621	0.284988
1	0.486551	4.711510	1.059411
25	0.533270	4.738126	0.591432
27	0.584362	4.951734	1.073784
18	0.491081	4.971271	1.127280
6	0.150537	1.214913	0.174503
4	0.135969	3.762130	0.261788
10	0.315652	4.063198	0.702622
14	0.349290	4.603769	1.477044
38	0.357355	4.633466	1.009066
40	0.480083	4.868918	1.491418
31	0.389410	4.890274	1.544914
2	0.137379	3.685122	0.087285
16	0.202898	4.008423	0.528119
20	0.296454	4.571820	1.302541
44	0.361646	4.602366	0.834563
46	0.418243	4.844030	1.316915
37	0.292513	4.865841	1.370411
14	0.256394	2.745988	0.440834
18	0.368012	4.041471	1.215256
42	0.349384	4.092510	0.747278
44	0.469160	4.467516	1.229630
35	0.350753	4.499143	1.283126
4	0.453522	3.731699	0.774423
28	0.469239	3.801985	0.306444
30	0.542572	4.280686	0.788796
21	0.444054	4.319353	0.842292
24	0.190020	1.124930	0.467979
26	0.252803	3.425565	0.014374
17	0.209234	3.516013	0.067869
2	0.269832	3.319987	0.482352
7	0.269192	3.420019	0.535848
9	0.279103	1.081805	0.053496

Correlations between environmental, genetic, geographical and climatic distances with Holm-Bonferroni correction

The climate distances are based on the 19 Bioclim variables, each represented by a distance matrix
Values are sortet by decreasing p valule

x	y	r	p	Holm-Bonferroni p value for significance at alpha=0.05	significant?
EnvDist	Bio1Dist	0.91554	0.00001	0.0006250	*
EnvDist	Bio5Dist	0.91258	0.00001	0.0006329	*
EnvDist	Bio8Dist	0.90237	0.00001	0.0006410	*
EnvDist	Bio9Dist	0.90434	0.00001	0.0006494	*
EnvDist	Bio10Dist	0.90402	0.00001	0.0006579	*
EnvDist	Bio11Dist	0.90237	0.00001	0.0006667	*
LnGeoDist	Bio1Dist	0.42727	0.00001	0.0006757	*
LnGeoDist	Bio4Dist	0.50071	0.00001	0.0006849	*
LnGeoDist	Bio9Dist	0.44859	0.00001	0.0006944	*
LnGeoDist	Bio10Dist	0.44985	0.00001	0.0007042	*
LnGeoDist	Bio8Dist	0.39303	0.00003	0.0007143	*
LnGeoDist	Bio11Dist	0.39303	0.00004	0.0007246	*
LnGeoDist	Bio6Dist	0.28241	0.00032	0.0007353	*
LnGeoDist	Bio15Dist	0.26897	0.00049	0.0007463	*
LnGeoDist	Bio5Dist	0.31444	0.00058	0.0007576	*
GenDist (FPTest)	Bio6Dist	0.19363	0.03659	0.0007692	ns
GenDist (FPTest)	Bio3Dist	-0.19444	0.04131	0.0007813	ns
GenDist (AllelRich)	Bio3Dist	-0.19179	0.04505	0.0007937	ns
GenDist (AllelRich)	Bio6Dist	0.18201	0.04702	0.0008065	ns
GenDist (Fst/(1-Fst))	Bio6Dist	0.20963	0.04831	0.0008197	ns
GenDist (Fst/(1-Fst))	Bio15Dist	0.17141	0.06293	0.0008333	ns
GenDist (FPTest)	Bio15Dist	0.13674	0.06780	0.0008475	ns
GenDist (AllelRich)	Bio15Dist	0.13526	0.06876	0.0008621	ns
GenDist (FPTest)	Bio2Dist	-0.16190	0.07915	0.0008772	ns
LnGeoDist	Bio12Dist	0.10440	0.09069	0.0008929	ns
GenDist (AllelRich)	Bio2Dist	-0.15171	0.10209	0.0009091	ns
LnGeoDist	Bio13Dist	0.09945	0.11376	0.0009259	ns
LnGeoDist	Bio16Dist	0.09880	0.11624	0.0009434	ns
LnGeoDist	Bio19Dist	0.09880	0.11735	0.0009615	ns
GenDist (Fst/(1-Fst))	Bio9Dist	0.17803	0.12612	0.0009804	ns
GenDist (Fst/(1-Fst))	Bio10Dist	0.17891	0.12907	0.0010000	ns
GenDist (Fst/(1-Fst))	Bio1Dist	0.16534	0.16711	0.0010204	ns
EnvDist	Bio15Dist	0.10513	0.17761	0.0010417	ns
GenDist (Fst/(1-Fst))	Bio3Dist	-0.14931	0.22781	0.0010638	ns
GenDist (FPTest)	Bio10Dist	0.11428	0.22862	0.0010870	ns
GenDist (FPTest)	Bio9Dist	0.11230	0.23204	0.0011111	ns
GenDist (AllelRich)	Bio10Dist	0.11179	0.24000	0.0011364	ns
GenDist (Fst/(1-Fst))	Bio11Dist	0.13711	0.24281	0.0011628	ns
GenDist (Fst/(1-Fst))	Bio8Dist	0.13711	0.24468	0.0011905	ns
GenDist (AllelRich)	Bio9Dist	0.10971	0.24667	0.0012195	ns
GenDist (Fst/(1-Fst))	Bio19Dist	-0.10270	0.24740	0.0012500	ns
GenDist (Fst/(1-Fst))	Bio16Dist	-0.10270	0.25023	0.0012821	ns
GenDist (Fst/(1-Fst))	Bio5Dist	0.13763	0.25946	0.0013158	ns
GenDist (FPTest)	Bio1Dist	0.10814	0.26062	0.0013514	ns
GenDist (Fst/(1-Fst))	Bio12Dist	-0.09493	0.27508	0.0013889	ns
GenDist (AllelRich)	Bio1Dist	0.10539	0.27532	0.0014286	ns
EnvDist	Bio3Dist	-0.10070	0.31761	0.0014706	ns
GenDist (FPTest)	Bio7Dist	-0.10692	0.31861	0.0015152	ns
EnvDist	Bio16Dist	-0.07132	0.33043	0.0015625	ns
EnvDist	Bio19Dist	-0.07132	0.33385	0.0016129	ns
GenDist (AllelRich)	Bio7Dist	-0.10308	0.33999	0.0016667	ns
GenDist (Fst/(1-Fst))	Bio2Dist	-0.11548	0.34446	0.0017241	ns
GenDist (Fst/(1-Fst))	Bio13Dist	-0.08028	0.37541	0.0017857	ns

GenDist (FPTest)	Bio8Dist	0.08259	0.38303	0.0018519	ns
GenDist (FPTest)	Bio11Dist	0.08259	0.38559	0.0019231	ns
GenDist (AllelRich)	Bio8Dist	0.08016	0.40074	0.0020000	ns
GenDist (AllelRich)	Bio11Dist	0.08016	0.40277	0.0020833	ns
EnvDist	Bio13Dist	-0.05929	0.42456	0.0021739	ns
EnvDist	Bio12Dist	-0.05586	0.43929	0.0022727	ns
EnvDist	Bio6Dist	0.05842	0.50089	0.0023810	ns
GenDist (FPTest)	Bio5Dist	0.05956	0.54797	0.0025000	ns
LnGeoDist	Bio2Dist	-0.04885	0.55098	0.0026316	ns
LnGeoDist	Bio7Dist	-0.05474	0.55142	0.0027778	ns
GenDist (AllelRich)	Bio5Dist	0.05742	0.56637	0.0029412	ns
GenDist (Fst/(1-Fst))	Bio4Dist	0.05244	0.61209	0.0031250	ns
EnvDist	Bio2Dist	-0.05129	0.61222	0.0033333	ns
GenDist (Fst/(1-Fst))	Bio7Dist	-0.06408	0.64888	0.0035714	ns
GenDist (AllelRich)	Bio13Dist	0.03404	0.65284	0.0038462	ns
EnvDist	Bio7Dist	-0.04993	0.65702	0.0041667	ns
GenDist (FPTest)	Bio13Dist	0.02844	0.70770	0.0045455	ns
GenDist (FPTest)	Bio4Dist	-0.02236	0.77635	0.0050000	ns
GenDist (AllelRich)	Bio4Dist	-0.02171	0.78775	0.0055556	ns
LnGeoDist	Bio3Dist	0.01256	0.88496	0.0062500	ns
EnvDist	Bio4Dist	0.01149	0.88712	0.0071429	ns
GenDist (FPTest)	Bio12Dist	-0.00395	0.95744	0.0083333	ns
GenDist (AllelRich)	Bio16Dist	0.00375	0.96022	0.0100000	ns
GenDist (AllelRich)	Bio19Dist	0.00375	0.96077	0.0125000	ns
GenDist (FPTest)	Bio16Dist	-0.00221	0.97680	0.0166667	ns
GenDist (FPTest)	Bio19Dist	-0.00221	0.97701	0.0250000	ns
GenDist (AllelRich)	Bio12Dist	0.00107	0.98856	0.0500000	ns
