# Supplementary material for: Geography of Genetic Structure in Barley Wild Relative Hordeum vulgare subsp. spontaneum in Jordan
Source: PLoS One. 2016 Aug 11;11(8):e0160745. doi: 10.1371/journal.pone.0160745 (PMC4981475; doi:10.1371/journal.pone.0160745)
Supplement: S1 Table — (PDF) [file pone.0160745.s002.pdf]

S1 Table. Microsatellite data for 373 *Spontaneum* individuals

Geography of genetic structure in barley wild relative *Hordeum vulgare* subsp. *spontaneum* in Jordan

PloS ONE

Thormann I. et al.

Individual	GBM1404	GBM1404	GBM1363	GBM1363	GBM1461	GBM1461	GBM1033	GBM1033
1-2	268	268	117	117	220	220	273	273
1-3	264	264	117	117	216	216	277	277
1-4	268	268	117	117	210	210	273	273
1-6	264	264	117	117	216	216	277	277
1-7	264	264	117	117	220	220	273	273
1-8	264	264	117	117	216	216	277	277
1-9	264	264	114	114	204	204	277	277
1-10	264	264	117	117	204	204	273	273
1-11	264	264	117	117	220	220	273	273
1-12	268	268	117	117	214	214	273	273
1-13	264	264	117	117	210	210	273	273
4-1	268	268	117	117	202	202	273	273
4-3	268	268	117	117	202	202	273	273
4-4	268	268	117	117	202	202	277	277
4-5	268	268	117	117	202	202	277	277
4-6	268	268	117	117	202	202	273	273
4-7	268	268	117	117	202	202	273	273
4-8	268	268	117	117	202	202	277	277
4-9	268	268	114	114	210	210	277	277
4-10	268	268	114	114	210	210	277	277
4-11	268	268	114	114	210	210	277	277
4-12	268	268	114	114	210	210	277	277
4-13	268	268	114	114	210	210	277	277
7-1	268	268	117	117	212	212	273	273
7-2	268	268	117	117	214	214	273	273
7-3	268	268	117	117	212	212	273	273
7-4	268	268	117	117	212	212	273	273
7-5	268	268	117	117	228	228	277	277
7-6	268	268	117	117	228	228	277	277
7-8	268	268	117	117	214	214	273	273
7-9	264	264	117	117	214	214	273	273
7-10	268	268	117	117	214	214	273	273
7-11	268	268	117	117	214	214	273	273
7-12	268	268	117	117	212	212	273	273
9-1	264	264	117	117	212	212	271	271
9-2	264	264	117	117	206	206	273	273
9-3	264	264	117	117	200	200	271	271
9-4	268	268	114	114	212	212	273	273
9-5	264	264	117	117	200	200	271	271
9-6	264	264	117	117	212	212	273	273
9-7	264	264	117	117	214	214	271	271
9-8	264	264	114	114	196	196	271	271
9-9	268	268	117	117	208	208	273	273
9-10	264	264	117	117	200	200	273	273
9-11	264	264	117	117	212	212	271	271
9-12	264	264	117	117	200	200	271	271
10-1	264	264	114	114	214	214	281	281
10-2	264	264	117	117	206	206	271	271
10-3	264	264	117	117	212	212	273	273
10-4	264	264	114	114	214	214	281	281
10-5	264	264	120	120	202	202	271	271
10-6	268	268	120	120	212	212	273	273
10-7	264	264	117	117	212	212	273	273
10-8	264	264	117	117	202	202	271	271
10-9	268	268	117	117	212	212	271	271
10-10	264	264	117	117	202	202	271	271
10-11	268	268	117	117	212	212	271	271

10-12	264	264	117	117	206	206	271	271
10-13	264	264	120	120	208	208	281	281
7561-10b	264	264	117	117	226	226	271	271
7561-11b	264	264	117	117	226	226	271	271
7561-12b	264	264	117	117	228	228	271	271
7561-1a	264	264	117	117	208	208	275	275
7561-2a	264	264	117	117	210	210	275	275
7561-3a	264	264	117	117	210	210	275	275
7561-4a	264	264	117	117	210	210	275	275
7561-5a	264	264	117	117	210	210	275	275
7561-6a	264	264	117	117	210	210	275	275
7561-7b	264	264	117	117	226	226	271	271
7561-8b	264	264	117	117	226	226	271	271
7561-9b	264	264	117	117	220	220	271	271
7566-1	264	264	117	117	224	224	275	275
7566-10	264	264	120	120	228	228	275	275
7566-2	264	264	117	117	228	228	275	275
7566-3	264	264	117	117	224	224	275	275
7566-4	264	264	117	117	202	202	275	275
7566-5	264	264	117	117	202	202	275	275
7566-6	264	264	114	114	218	218	271	271
7566-8	264	264	120	120	228	228	275	275
7566a-11	264	264	117	117	224	224	275	275
7566a-12	264	264	117	117	224	224	275	275
7566a-7	264	264	120	120	228	228	275	275
7566a-9	264	264	120	120	228	228	275	275
7568-10	264	264	114	114	202	202	271	271
7568-11	264	264	114	114	202	202	271	271
7568-12	264	264	114	114	202	202	271	271
7568-13	264	264	117	117	208	208	271	271
7568-1i	264	264	114	114	202	202	271	271
7568-1ii	268	268	117	117	214	214	273	273
7568-2	268	268	117	117	212	212	273	273
7568-3	268	268	117	117	222	222	273	273
7568-4	264	264	114	114	202	202	271	271
7568-5	264	264	117	117	222	222	275	275
7568-6	264	264	117	117	200	200	273	273
7568-7	264	264	114	114	202	202	271	271
7568-8	264	264	114	114	218	218	271	271
7568-9	264	264	114	114	202	202	271	271
7569-1	264	264	117	117	238	238	271	271
7569-10	264	264	117	117	228	228	271	271
7569-11	264	264	117	117	238	238	271	271
7569-12	264	264	117	117	238	238	271	271
7569-13	264	264	117	117	238	238	271	271
7569-2	264	264	117	117	238	238	271	271
**7569-3**	**268**	**268**	**117**	**117**	**210**	**210**	**279**	**279**
7569-4	264	264	117	117	238	238	271	271
7569-5	264	264	117	117	238	238	271	271
7569-6	264	264	117	117	238	238	271	271
7569-8	261	264	117	117	238	238	271	271
7569-9	264	264	117	117	238	238	271	271
7570-1	264	264	117	117	210	210	273	273
7570-10	264	264	117	117	210	210	273	273
7570-11	264	264	117	117	210	210	273	273
7570-12	264	264	117	117	210	210	273	273
7570-13	264	264	117	117	198	198	273	273
7570-2	268	268	117	117	210	210	271	271
7570-3	264	264	117	117	210	210	273	273
7570-4	264	264	114	114	210	210	271	271
7570-5	268	268	117	117	210	210	271	271
7570-6	264	264	117	117	210	210	273	273
7570-7	264	264	114	114	210	210	271	271
7570-8	264	264	120	120	210	210	273	273

7570-9	264	264	117	117	210	210	273	273
7571-10	264	264	120	120	220	220	271	271
7571-12	264	264	117	117	202	202	271	271
7571-13	264	264	117	117	206	206	271	271
7571-1a	264	264	117	117	218	218	275	275
7571-2	264	264	117	117	210	210	271	271
7571-3	264	264	117	117	206	206	271	271
7571-4	264	264	117	117	218	218	275	275
7571-5	264	264	117	117	218	218	273	273
7571-6	264	264	117	117	210	210	271	271
7571-7	264	264	117	117	210	210	273	273
7571-9	264	264	117	117	206	206	271	271
7575-1	264	264	117	117	220	220	271	271
7575-2	264	264	117	117	222	222	271	271
7575-9	264	264	117	117	222	222	271	271
7575a-10	264	264	117	117	222	222	271	271
7575a-11	264	264	117	117	222	222	271	271
7575a-12	264	264	117	117	222	222	271	271
7575a-13	264	264	117	117	222	222	271	271
7575a-3	264	264	117	117	222	222	271	271
7575a-4	264	264	117	117	222	222	271	271
7575a-5	264	264	117	117	222	222	271	271
7575a-6	264	264	117	117	220	220	271	271
7575a-7	264	264	117	117	210	210	271	271
7577-1	264	264	117	117	216	216	273	273
7577-10	264	264	117	117	216	216	273	273
7577-11	268	268	117	117	196	196	281	281
7577-2	264	264	117	117	216	216	273	273
7577-3	264	264	117	117	214	214	273	273
7577-4	264	264	117	117	216	216	271	271
7577-5	264	264	117	117	214	214	273	273
7577-6	264	264	117	117	212	212	273	273
7577-7	264	264	117	117	216	216	273	273
7577-8	264	264	117	117	214	214	273	273
7577-9	268	268	120	120	214	214	271	271
7581-10	264	264	123	123	214	214	271	271
7581-11	264	264	117	117	212	212	273	273
7581-12	264	264	117	117	212	212	271	271
7581-2	268	268	117	117	208	208	273	273
7581-3	268	268	117	117	208	208	275	275
7581-4	264	264	114	114	212	212	271	271
7581-5	264	264	117	117	210	210	273	273
7581-6	264	264	117	117	208	208	275	275
7581-7	264	264	117	117	210	210	271	271
7581-8	264	264	117	117	210	210	273	273
7581-9	264	264	120	120	216	216	273	273
7583-1	264	264	120	120	202	202	273	273
7583-10	264	264	120	120	204	204	271	271
7583-11	264	264	120	120	204	204	271	271
7583-12	264	264	120	120	204	204	277	277
7583-2	264	264	120	120	202	202	273	273
7583-3	264	264	117	117	210	210	273	273
7583-4	264	264	120	120	204	204	271	271
7583-5	264	264	120	120	204	204	271	271
7583-6	264	264	120	120	204	204	271	271
7583-7	264	264	120	120	204	204	271	271
7583-8	264	264	120	120	204	204	271	271
7583-9	264	264	120	120	204	204	271	271
7584-1	268	268	117	117	202	202	271	271
7584-10	264	264	120	120	218	218	273	273
7584-11	264	264	117	117	202	202	271	271
7584-12	268	268	117	117	220	220	273	273
7584-2	264	264	117	117	218	218	273	273
7584-3	264	264	120	120	206	206	271	271

7584-4	264	264	117	117	212	212	273	273
7584-5	264	264	117	117	202	202	277	277
7584-6	268	268	117	117	218	218	273	273
7584-7	268	268	120	120	218	218	271	271
7584-8	264	264	117	117	202	202	277	277
7584-9	264	264	120	120	206	206	271	271
7586-1	264	264	114	114	204	204	271	271
7586-10	264	264	117	117	204	204	271	271
7586-11	264	264	114	114	204	204	271	271
7586-12	261	261	114	114	204	204	271	271
7586-2	264	264	114	114	204	204	281	281
7586-3	264	264	127	127	237	237	273	273
7586-4	264	264	114	114	204	204	271	271
7586-5	264	264	114	114	204	204	279	279
7586-6	264	264	114	114	204	204	271	271
7586-7	264	264	117	117	206	206	271	271
7586-8	264	264	114	114	204	204	271	271
7586-9	264	264	114	114	204	204	271	271
7588-10	264	264	117	117	226	226	271	271
7588-11	264	264	117	117	204	204	279	279
7588-12	264	264	117	117	204	204	279	279
7588-14	264	264	117	117	210	210	271	271
7588-2	264	264	117	117	226	226	271	271
7588-3	264	264	117	117	210	210	271	271
7588-4	264	264	117	117	210	210	271	271
7588-5	264	264	117	117	208	208	271	271
7588-6	264	264	117	117	210	210	271	271
7588-7	264	264	117	117	210	210	271	271
7588-8	268	268	120	120	202	202	271	271
7588-9	264	264	117	117	228	228	279	279
7594-1	264	264	117	117	222	222	275	275
7594-10	264	264	117	117	192	192	275	275
7594-11	264	264	117	117	192	192	271	271
7594-12	264	264	117	117	200	200	275	275
7594-2	264	264	117	117	202	202	275	275
7594-3	264	264	117	117	200	200	275	275
7594-4	264	264	120	120	204	204	271	271
7594-5	268	268	117	117	210	210	273	273
7594-6	268	268	117	117	210	210	273	273
7594-7	268	268	117	117	208	208	273	273
7594-8	264	264	117	117	200	200	275	275
7594-9	264	264	117	117	206	206	271	271
7595-1	268	268	120	120	212	212	271	271
7595-10	261	261	114	114	226	226	271	271
7595-11	264	264	117	117	224	224	271	271
7595-12	264	264	117	117	224	224	271	271
7595-13	264	264	120	120	212	212	271	271
7595-3	261	261	114	114	226	226	271	271
7595-4	261	261	114	114	226	226	271	271
7595-5	268	268	117	117	202	202	271	271
7595-6	261	261	114	114	228	228	271	271
7595-7	261	261	117	117	192	192	271	271
7595-8	261	261	117	117	200	200	271	271
7595-9	264	264	114	114	214	214	273	273
7596-1	264	264	123	123	224	224	273	273
7596-10	261	261	117	117	224	224	277	277
7596-11	268	268	117	117	200	200	271	271
7596-12	268	268	117	117	224	224	271	271
7596-2	261	261	120	120	216	216	279	279
7596-3	264	264	120	120	200	200	279	279
7596-4	264	264	117	117	214	214	275	275
7596-5	264	264	117	117	222	222	273	273
7596-7	264	264	117	117	200	200	273	273
7596-8	264	264	120	120	218	218	271	271

7596-9	264	264	120	120	212	212	273	273
7599-1	264	264	117	117	224	224	273	273
7599-10	268	268	117	117	224	224	271	271
7599-11	264	264	117	117	210	210	273	273
7599-12	264	264	117	117	224	224	273	273
7599-13	264	264	117	117	226	226	273	273
7599-3	268	268	114	114	224	224	273	273
7599-4	264	264	114	114	198	198	273	273
7599-5	264	264	114	114	202	202	271	271
7599-6	264	264	114	114	196	196	273	273
7599-7	264	264	117	117	210	210	273	273
7599-8	264	264	117	117	212	212	271	271
7599-9	264	264	117	117	212	212	271	271
7600-1	261	261	117	117	200	200	271	271
7600-10	261	261	114	114	210	210	275	275
7600-11	264	264	117	117	210	210	275	275
7600-2	264	264	117	117	218	218	275	275
7600-3	264	264	117	117	222	222	273	273
7600-4	264	264	117	117	218	218	275	275
7600-5	264	264	117	117	204	204	273	273
7600-6	264	264	117	117	204	204	273	273
7600-7	264	264	117	117	204	204	273	273
7600-8	261	261	114	114	210	210	275	275
7600-9	261	261	114	114	210	210	275	275
7602-10	264	264	117	117	222	222	275	275
7602-11	264	264	117	117	210	210	273	273
7602-12	264	264	117	117	210	210	273	273
7602-2	264	264	117	117	222	222	275	275
7602-4	264	264	117	117	224	224	275	275
7602-5	264	264	117	117	222	222	275	275
7602-6	264	264	117	117	222	222	275	275
7602-7	264	264	117	117	224	224	275	275
7602-9	264	264	117	117	210	210	275	275
7604-1	261	261	117	117	220	220	277	277
7604-10	264	264	117	117	222	222	275	275
7604-11	264	264	117	117	222	222	275	275
7604-12	264	264	117	117	208	208	277	277
7604-13	264	264	117	117	222	222	275	275
7604-2	264	264	117	117	222	222	275	275
7604-3	264	264	117	117	222	222	275	275
7604-4	264	264	117	117	224	224	275	275
7604-6	264	264	117	117	224	224	275	275
7604-7	268	268	117	117	206	206	273	273
7604-8	264	264	117	117	220	220	275	275
7604-9	264	264	117	117	222	222	275	275
7606-1	264	264	117	117	210	210	273	273
7606-10	264	264	114	114	224	224	275	275
7606-11	264	264	117	117	218	218	273	273
7606-12	264	264	117	117	218	218	273	273
7606-3	264	264	117	117	218	218	273	273
7606-4	268	268	117	117	206	206	273	273
7606-5	264	264	120	120	212	212	273	273
7606-6	264	264	120	120	212	212	273	273
7606-7	264	264	117	117	220	220	275	275
7606-8	264	264	117	117	220	220	275	275
7606-9	264	264	117	117	218	218	273	273
7610-1	264	264	117	117	204	204	279	279
7610-10	264	264	117	117	198	198	281	281
7610-11	268	268	117	117	198	198	273	273
7610-12	264	264	117	117	198	198	281	281
7610-2	264	264	117	117	202	202	279	279
7610-3	261	264	117	117	208	208	273	273
7610-4	264	264	117	117	224	224	281	281
7610-5	268	268	117	117	198	198	271	271

7610-6	268	268	117	117	200	200	271	271
7610-7	268	268	117	117	200	200	271	271
7610-8	268	268	117	117	200	200	271	271
7610-9	268	268	117	117	206	206	271	271
7612-1	264	264	117	117	224	224	275	275
7612-10	264	264	117	117	224	224	275	275
7612-11	268	268	117	117	220	220	275	275
7612-12	264	264	117	117	222	222	275	275
7612-13	264	264	117	117	222	222	275	275
7612-2	264	264	117	117	222	222	275	275
7612-3	264	264	117	117	224	224	275	275
7612-4	264	264	114	114	224	224	275	275
7612-5	264	264	117	117	224	224	275	275
7612-6	264	264	117	117	222	222	275	275
7612-8	268	268	114	114	202	202	273	273
7612-9	264	264	117	117	220	220	275	275
7616-1	264	264	114	114	224	224	275	275
7616-10	264	264	114	114	224	224	275	275
7616-13	264	264	114	114	224	224	275	275
7616-14	264	264	114	114	222	222	275	275
7616-1ii	261	261	117	117	200	200	271	271
7616-2	264	264	114	114	224	224	275	275
7616-3	264	264	117	117	208	208	279	279
7616-4	264	264	114	114	224	224	275	275
7616-5	264	264	114	114	224	224	275	275
7616-6	264	264	114	114	224	224	275	275
7616-7	264	264	117	117	222	222	275	275
7616-8	261	261	117	117	212	212	273	273
7616-9i	264	264	114	114	224	224	275	275
7625-1	268	268	117	117	210	210	277	277
7625-10	268	268	117	117	210	210	277	277
7625-11	268	268	114	114	214	214	273	273
7625-2	268	268	117	117	210	210	277	277
7625-4	268	268	117	117	210	210	277	277
7625-5	268	268	117	117	210	210	277	277
7625-7	268	268	117	117	210	210	277	277
7625-8	264	264	117	117	210	210	277	277
7628-1	268	268	117	117	214	214	273	273
7628-10	268	268	117	117	214	214	273	273
7628-11	268	268	117	117	214	214	273	273
7628-12	264	264	117	117	202	202	277	277
7628-2	268	268	117	117	214	214	273	273
7628-3	268	268	114	114	210	210	277	277
7628-4	268	268	114	114	210	210	277	277
7628-5	268	268	117	117	214	214	273	273
7628-6	268	268	117	117	214	214	277	277
7628-7	264	264	117	117	216	216	273	273
7628-8	268	268	117	117	214	214	273	273
7628-9	268	268	117	117	214	214	273	273
7640-1	268	268	117	117	204	204	273	273
7640-11	264	264	117	117	216	216	271	271
7640-12	264	264	117	117	210	210	273	273
7640-2	264	264	117	117	210	210	273	273
7640-3	264	264	117	117	210	210	273	273
7640-4	264	264	117	117	238	238	271	271
7640-5	264	264	120	120	238	238	281	281
7640-6	264	264	117	117	210	210	271	271
7640-7	264	264	120	120	210	210	273	273
7640-8	264	264	120	120	208	208	273	273
7640-9	264	264	120	120	208	208	271	271

Individual	GBM1110	GBM1110	GBM1326	GBM1326	GBM1013	GBM1013	GBM1015	GBM1015
1-2	227	227	157	157	160	160	227	227
1-3	233	233	151	151	160	160	231	231
1-4	225	225	151	151	160	160	219	219
1-6	233	233	157	157	160	160	231	231
1-7	237	237	151	151	160	160	231	231
1-8	231	231	151	151	160	160	227	227
1-9	233	233	151	151	160	160	231	231
1-10	225	225	151	151	160	160	231	231
1-11	233	233	151	151	160	160	227	227
1-12	233	233	151	151	160	160	231	231
1-13	233	233	151	151	160	160	231	231
4-1	237	237	160	160	160	160	255	255
4-3	235	235	160	160	160	160	255	255
4-4	233	233	151	151	160	160	255	255
4-5	233	233	151	151	160	160	255	255
4-6	237	237	160	160	160	160	255	255
4-7	237	237	160	160	160	160	255	255
4-8	235	235	151	151	160	160	255	255
4-9	237	237	160	160	160	160	235	235
4-10	237	237	160	160	160	160	235	235
4-11	237	237	160	160	160	160	235	235
4-12	237	237	160	160	160	160	235	235
4-13	237	237	160	160	160	160	235	235
7-1	235	235	160	160	160	160	235	235
7-2	235	235	160	160	160	160	235	235
7-3	235	235	160	160	160	160	235	235
7-4	235	235	160	160	160	160	235	235
7-5	235	235	160	160	160	160	227	227
7-6	233	233	160	160	160	160	227	227
7-8	235	235	160	160	160	160	263	263
7-9	235	235	157	157	160	160	263	263
7-10	235	237	160	160	160	160	263	263
7-11	237	237	160	160	160	160	263	263
7-12	235	235	160	160	160	160	242	242
9-1	233	233	154	154	163	163	227	227
9-2	235	235	154	154	160	160	235	235
9-3	233	233	173	173	160	160	223	223
9-4	231	231	154	154	163	163	223	223
9-5	233	233	173	173	160	160	223	223
9-6	237	237	154	154	163	163	235	235
9-7	233	233	154	154	163	163	223	223
9-8	233	233	151	151	160	160	206	206
9-9	237	237	154	154	163	163	223	223
9-10	233	233	173	173	163	163	223	223
9-11	233	233	154	154	163	163	227	227
9-12	233	233	173	173	160	160	223	223
10-1	237	237	173	173	160	160	214	214
10-2	233	233	173	173	163	163	223	223
10-3	235	235	173	173	160	160	223	223
10-4	233	233	148	148	160	160	227	227
10-5	237	237	151	151	160	160	227	227
10-6	233	233	154	154	160	160	194	194
10-7	237	237	154	154	160	160	227	227
10-8	237	237	173	173	163	163	206	206
10-9	233	233	151	151	163	163	223	223
10-10	237	237	173	173	163	163	206	206
10-11	233	233	151	151	163	163	223	223

10-12	235	235	154	154	163	163	206	206
10-13	233	233	164	164	160	160	223	223
7561-10b	227	227	157	157	167	167	227	227
7561-11b	227	227	157	157	167	167	227	227
7561-12b	227	227	157	157	167	167	227	227
7561-1a	227	227	151	151	157	157	223	223
7561-2a	227	227	151	151	157	157	223	223
7561-3a	227	227	151	151	157	157	223	223
7561-4a	227	227	151	151	157	157	223	223
7561-5a	227	227	151	151	157	157	223	223
7561-6a	227	227	151	151	157	157	223	223
7561-7b	227	227	157	157	167	167	227	227
7561-8b	227	227	157	157	167	167	227	227
7561-9b	227	227	157	157	167	167	227	227
7566-1	237	237	157	157	160	160	227	227
7566-10	233	233	151	151	163	163	227	227
7566-2	237	237	157	157	160	160	227	227
7566-3	237	237	157	157	160	160	227	227
7566-4	231	231	154	154	160	160	231	231
7566-5	231	231	154	154	160	160	231	231
7566-6	227	227	157	157	157	157	231	231
7566-8	233	233	151	151	163	163	227	227
7566a-11	235	235	148	148	163	163	227	227
7566a-12	237	237	157	157	160	160	227	227
7566a-7	233	233	151	151	163	163	227	227
7566a-9	233	233	151	151	163	163	227	227
7568-10	233	233	157	157	160	160	242	242
7568-11	233	233	157	157	160	160	242	242
7568-12	233	233	157	157	160	160	242	242
7568-13	233	233	154	154	160	160	219	219
7568-1i	233	233	157	157	160	160	242	242
7568-1ii	231	231	167	167	160	160	210	210
7568-2	231	231	167	167	160	160	210	210
7568-3	235	235	151	151	160	160	235	235
7568-4	231	231	157	157	160	160	242	242
7568-5	227	227	164	164	157	157	227	227
7568-6	229	229	151	151	160	160	247	247
7568-7	233	233	157	157	160	160	242	242
7568-8	233	233	157	157	160	160	227	227
7568-9	233	233	157	157	160	160	242	242
7569-1	235	235	148	148	160	160	219	219
7569-10	233	233	148	148	160	160	219	219
7569-11	233	233	148	148	160	160	219	219
7569-12	233	233	148	148	160	160	219	219
7569-13	233	233	148	148	160	160	219	219
7569-2	233	233	148	148	160	160	219	219
**7569-3**	**233**	**233**	**154**	**154**	**154**	**154**	**223**	**223**
7569-4	233	233	148	148	160	160	219	219
7569-5	233	233	148	148	160	160	219	219
7569-6	233	233	148	148	160	160	219	219
7569-8	233	233	148	148	160	160	219	219
7569-9	233	233	148	148	160	160	219	219
7570-1	221	221	164	164	160	160	227	227
7570-10	233	233	157	157	167	167	223	223
7570-11	233	233	157	157	160	160	223	223
7570-12	233	233	157	157	167	167	223	223
7570-13	233	233	154	154	160	160	223	223
7570-2	233	233	157	157	160	160	227	227
7570-3	233	233	157	157	167	167	223	223
7570-4	221	221	164	164	160	160	210	210
7570-5	233	233	154	154	160	160	210	210
7570-6	233	233	157	157	167	167	223	223
7570-7	221	221	164	164	160	160	210	210
7570-8	233	233	154	154	167	167	227	227

7570-9	233	233	157	157	167	167	223	223
7571-10	231	231	154	154	160	160	239	239
7571-12	233	233	154	154	160	160	210	210
7571-13	233	233	160	160	160	160	239	239
7571-1a	235	235	154	154	160	160	223	223
7571-2	229	229	154	154	167	167	239	239
7571-3	233	233	160	160	160	160	239	239
7571-4	233	233	154	154	160	160	223	223
7571-5	235	235	167	167	163	163	239	239
7571-6	233	233	154	154	167	167	239	239
7571-7	231	231	154	154	167	167	239	239
7571-9	233	233	160	160	160	160	239	239
7575-1	233	233	164	164	157	157	223	223
7575-2	235	235	164	164	157	157	223	223
7575-9	235	235	164	164	157	157	223	223
7575a-10	235	235	164	164	157	157	223	223
7575a-11	235	235	164	164	157	157	223	223
7575a-12	235	235	164	164	157	157	223	223
7575a-13	235	235	164	164	157	157	223	223
7575a-3	235	235	164	164	157	157	223	223
7575a-4	233	233	164	164	157	157	223	223
7575a-5	235	235	164	164	157	157	223	223
7575a-6	235	235	164	164	157	157	223	223
7575a-7	233	233	164	164	157	157	223	223
7577-1	227	227	148	148	160	160	227	227
7577-10	227	227	148	148	160	160	227	227
7577-11	231	231	154	154	160	160	247	247
7577-2	227	227	148	148	160	160	227	227
7577-3	227	227	148	148	160	160	227	227
7577-4	233	233	148	148	160	160	247	247
7577-5	227	227	148	148	160	160	227	227
7577-6	227	227	151	151	160	160	242	242
7577-7	227	227	148	148	160	160	227	227
7577-8	227	227	148	148	160	160	227	227
7577-9	235	235	154	154	160	160	214	214
7581-10	231	231	154	154	160	160	227	227
7581-11	235	235	151	151	160	160	227	227
7581-12	235	235	151	151	160	160	227	227
7581-2	235	235	151	151	160	160	223	223
7581-3	235	235	154	154	160	160	223	223
7581-4	231	231	151	151	160	160	210	210
7581-5	231	231	151	151	160	160	210	210
7581-6	221	221	151	151	160	160	227	227
7581-7	231	231	151	151	160	160	223	223
7581-8	231	231	151	151	160	160	210	210
7581-9	237	237	151	151	160	160	227	227
7583-1	231	231	160	160	160	160	219	219
7583-10	233	233	151	151	160	160	227	227
7583-11	231	231	151	151	160	160	227	227
7583-12	235	235	157	157	160	160	227	227
7583-2	231	231	160	160	160	160	219	219
7583-3	221	221	154	154	163	163	227	227
7583-4	233	233	151	151	160	160	227	227
7583-5	233	233	151	151	160	160	227	227
7583-6	231	231	151	151	160	160	227	227
7583-7	233	233	151	151	160	160	227	227
7583-8	235	235	151	151	160	160	227	227
7583-9	233	233	151	151	160	160	227	227
7584-1	247	247	151	151	160	160	227	227
7584-10	233	233	145	145	160	160	219	219
7584-11	233	233	170	170	157	157	219	219
7584-12	233	233	154	154	160	160	219	219
7584-2	233	233	145	145	163	163	231	231
7584-3	221	221	154	154	163	163	231	231

7584-4	235	235	154	154	160	160	227	227
7584-5	231	231	157	157	163	163	263	263
7584-6	233	233	154	154	160	160	219	219
7584-7	233	233	154	154	160	160	219	219
7584-8	231	231	157	157	163	163	263	263
7584-9	221	221	154	154	163	163	231	231
7586-1	231	231	160	160	160	160	214	214
7586-10	231	231	160	160	160	160	223	223
7586-11	231	231	160	160	160	160	214	214
7586-12	231	231	160	160	160	160	214	214
7586-2	231	231	160	160	160	160	206	206
7586-3	231	231	154	154	160	160	227	227
7586-4	231	231	160	160	160	160	214	214
7586-5	233	233	154	154	163	163	239	239
7586-6	231	231	154	154	160	160	214	214
7586-7	237	237	160	160	160	160	259	259
7586-8	231	231	160	160	160	160	214	214
7586-9	231	231	160	160	160	160	214	214
7588-10	221	221	151	151	160	160	219	219
7588-11	233	233	154	154	157	157	242	242
7588-12	233	233	154	154	160	160	242	242
7588-14	233	233	157	157	167	167	223	223
7588-2	221	221	151	151	160	160	219	219
7588-3	229	229	154	154	160	160	235	235
7588-4	231	231	154	154	157	157	239	239
7588-5	231	231	154	154	160	160	231	231
7588-6	231	231	154	154	160	160	239	239
7588-7	231	231	157	157	167	167	223	223
7588-8	233	233	154	154	160	160	239	239
7588-9	219	219	151	151	160	160	219	219
7594-1	227	227	157	157	160	160	210	210
7594-10	233	233	151	151	160	160	219	219
7594-11	233	233	164	164	160	160	227	227
7594-12	235	235	157	157	160	160	210	210
7594-2	233	233	151	151	160	160	219	219
7594-3	235	235	157	157	160	160	210	210
7594-4	233	233	148	148	160	160	219	219
7594-5	233	233	160	160	160	160	231	231
7594-6	233	233	160	160	160	160	231	231
7594-7	231	231	160	160	160	160	231	231
7594-8	235	235	157	157	160	160	210	210
7594-9	231	231	151	151	160	160	251	251
7595-1	235	235	164	164	160	160	223	223
7595-10	233	233	164	164	160	160	223	223
7595-11	233	233	164	164	160	160	219	219
7595-12	233	233	164	164	160	160	219	219
7595-13	231	231	164	164	160	160	223	223
7595-3	233	233	164	164	160	160	223	223
7595-4	231	231	164	164	160	160	223	223
7595-5	233	233	160	160	172	172	223	223
7595-6	233	233	164	164	160	160	223	223
7595-7	235	235	151	151	160	160	219	219
7595-8	233	233	164	164	160	160	219	219
7595-9	233	233	151	151	160	160	219	219
7596-1	235	235	157	157	160	160	210	210
7596-10	239	239	160	160	160	160	219	219
7596-11	227	227	160	160	160	160	210	210
7596-12	221	221	151	151	160	160	239	239
7596-2	239	239	157	157	170	170	235	235
7596-3	231	231	157	157	160	160	210	210
7596-4	235	235	151	151	160	160	210	210
7596-5	235	235	151	151	160	160	210	210
7596-7	235	235	151	151	160	160	210	210
7596-8	229	229	160	160	160	160	210	210

7596-9	235	235	157	157	160	160	210	210
7599-1	235	235	157	157	160	160	227	227
7599-10	233	233	157	157	160	160	251	251
7599-11	237	237	157	157	160	160	223	223
7599-12	233	233	157	157	160	160	227	227
7599-13	227	227	151	151	160	160	247	247
7599-3	233	233	157	157	160	160	227	227
7599-4	231	231	164	164	160	160	210	210
7599-5	233	233	157	157	160	160	227	227
7599-6	237	237	164	164	167	167	227	227
7599-7	237	237	157	157	160	160	223	223
7599-8	231	231	157	157	160	160	223	223
7599-9	231	231	157	157	160	160	223	223
7600-1	235	235	151	151	167	167	219	219
7600-10	233	233	160	160	160	160	223	223
7600-11	227	227	151	151	157	157	223	223
7600-2	233	233	160	160	160	160	219	219
7600-3	233	233	160	160	167	167	219	219
7600-4	231	231	160	160	160	160	219	219
7600-5	233	233	157	157	167	167	219	219
7600-6	233	233	157	157	167	167	219	219
7600-7	233	233	157	157	160	160	223	223
7600-8	233	233	160	160	160	160	223	223
7600-9	233	233	160	160	160	160	223	223
7602-10	237	237	164	164	157	157	227	227
7602-11	227	227	154	154	157	157	227	227
7602-12	227	227	154	154	157	157	227	227
7602-2	227	227	148	148	157	157	223	223
7602-4	227	227	154	154	160	160	231	231
7602-5	227	227	154	154	157	157	223	223
7602-6	227	227	164	164	160	160	227	227
7602-7	233	233	148	148	160	160	223	223
7602-9	227	227	164	164	160	160	231	231
7604-1	227	227	164	164	160	160	227	227
7604-10	227	227	154	154	160	160	227	227
7604-11	227	227	164	164	160	160	227	227
7604-12	227	227	154	154	160	160	227	227
7604-13	227	227	154	154	160	160	223	223
7604-2	227	227	164	164	160	160	227	227
7604-3	227	227	164	164	160	160	227	227
7604-4	227	227	154	154	160	160	227	227
7604-6	227	227	154	154	160	160	227	227
7604-7	235	235	148	148	160	160	227	227
7604-8	227	227	154	154	160	160	227	227
7604-9	227	227	164	164	160	160	227	227
7606-1	227	227	157	157	160	160	227	227
7606-10	237	237	148	148	160	160	227	227
7606-11	233	233	157	157	160	160	231	231
7606-12	233	233	157	157	160	160	231	231
7606-3	233	233	151	151	160	160	227	227
7606-4	233	233	154	154	160	160	227	227
7606-5	225	225	160	160	167	167	227	227
7606-6	225	225	160	160	167	167	227	227
7606-7	227	227	164	164	160	160	227	227
7606-8	227	227	164	164	160	160	227	227
7606-9	233	233	157	157	160	160	231	231
7610-1	233	233	160	160	160	160	227	227
7610-10	233	233	151	151	160	160	227	227
7610-11	237	237	157	157	160	160	231	231
7610-12	233	233	151	151	160	160	227	227
7610-2	227	227	160	160	160	160	223	227
7610-3	233	233	157	157	160	160	227	227
7610-4	233	233	151	151	170	170	231	231
7610-5	237	237	151	151	160	160	219	219

7610-6	235	235	157	157	160	160	219	219
7610-7	233	233	151	151	160	160	219	219
7610-8	233	233	151	151	160	160	219	219
7610-9	231	231	151	151	160	160	227	227
7612-1	227	227	148	148	160	160	227	227
7612-10	227	227	148	148	160	160	227	227
7612-11	227	227	164	164	157	157	223	223
7612-12	227	227	164	164	160	160	227	227
7612-13	225	225	148	148	160	160	227	227
7612-2	233	233	154	154	160	160	223	223
7612-3	227	227	148	148	160	160	227	227
7612-4	227	227	154	154	160	160	242	242
7612-5	233	233	154	154	160	160	231	231
7612-6	227	227	154	154	157	157	223	223
7612-8	237	237	154	154	160	160	227	227
7612-9	227	227	164	164	160	160	227	227
7616-1	237	237	148	148	160	160	227	227
7616-10	237	237	148	148	160	160	227	227
7616-13	237	237	148	148	160	160	227	227
7616-14	237	237	164	164	160	160	223	223
7616-1ii	233	233	151	151	167	167	219	219
7616-2	237	237	148	148	160	160	227	227
7616-3	227	227	151	151	160	160	206	206
7616-4	237	237	148	148	160	160	227	227
7616-5	237	237	148	148	160	160	227	227
7616-6	237	237	148	148	160	160	227	227
7616-7	227	227	151	151	157	157	231	231
7616-8	237	237	151	151	170	170	198	198
7616-9i	237	237	148	148	160	160	227	227
7625-1	235	237	160	160	160	160	231	231
7625-10	237	237	160	160	160	160	235	235
7625-11	235	237	160	160	160	160	251	251
7625-2	235	235	160	160	160	160	235	235
7625-4	235	237	160	160	160	160	235	235
7625-5	237	237	160	160	160	160	235	235
7625-7	235	237	160	160	160	160	235	235
7625-8	237	237	160	160	160	160	235	235
7628-1	233	233	160	160	160	160	235	235
7628-10	233	233	160	160	160	160	235	235
7628-11	233	233	160	160	160	160	235	235
7628-12	235	235	160	160	160	160	235	235
7628-2	235	235	160	160	160	160	280	280
7628-3	235	235	160	160	160	160	293	293
7628-4	235	235	160	160	160	160	293	293
7628-5	235	235	160	160	160	160	235	235
7628-6	235	235	160	160	160	160	235	235
7628-7	231	231	157	157	160	160	231	231
7628-8	233	233	160	160	160	160	235	235
7628-9	233	233	160	160	160	160	235	235
7640-1	221	221	151	151	160	160	242	242
7640-11	233	233	164	164	160	160	247	247
7640-12	231	231	154	154	160	160	223	223
7640-2	233	233	157	157	160	160	239	239
7640-3	231	231	154	154	160	160	223	223
7640-4	221	221	167	167	167	167	219	219
7640-5	233	233	157	157	163	163	239	239
7640-6	233	233	157	157	160	160	247	247
7640-7	233	233	157	157	160	160	223	223
7640-8	235	235	157	157	160	160	223	223
7640-9	233	233	157	157	160	160	239	239

Individual	GBM1176	GBM1176	GBM1043	GBM1043	GBM1031	GBM1031	GBM1212	GBM1212
1-2	286	286	209	209	284	284	103	103
1-3	286	286	206	206	284	284	103	103
1-4	286	286	206	206	284	284	103	103
1-6	286	286	206	206	284	284	103	103
1-7	286	286	206	206	284	284	103	103
1-8	286	286	206	206	284	284	103	103
1-9	286	286	206	206	284	284	103	103
1-10	284	284	203	203	284	284	103	103
1-11	284	284	203	203	284	284	103	103
1-12	286	286	206	206	284	284	106	106
1-13	286	286	206	206	284	284	103	103
4-1	286	286	206	206	284	284	103	103
4-3	286	286	206	206	284	284	103	103
4-4	286	286	206	206	284	284	106	106
4-5	286	286	206	206	284	284	106	106
4-6	286	286	206	206	284	284	103	103
4-7	286	286	206	206	284	284	103	103
4-8	286	286	206	206	284	284	106	106
4-9	286	286	206	206	284	284	106	106
4-10	286	286	206	206	284	284	106	106
4-11	286	286	206	206	284	284	106	106
4-12	286	286	206	206	284	284	106	106
4-13	286	286	206	206	284	284	106	106
7-1	286	286	206	206	284	284	103	103
7-2	286	286	206	206	284	284	103	103
7-3	286	286	206	206	284	284	103	103
7-4	286	286	206	206	284	284	103	103
7-5	286	286	206	206	284	284	106	106
7-6	286	286	209	209	284	284	106	106
7-8	284	284	206	206	284	284	103	103
7-9	286	286	206	206	284	284	106	106
7-10	284	284	206	206	284	284	103	103
7-11	284	284	206	206	284	284	103	103
7-12	284	284	206	206	284	284	103	103
9-1	286	286	209	209	284	284	103	103
9-2	286	286	206	206	284	284	103	103
9-3	284	284	209	209	284	284	103	103
9-4	284	284	206	206	284	284	106	106
9-5	284	284	206	206	284	284	103	103
9-6	284	284	206	206	284	284	103	103
9-7	284	284	209	209	284	284	106	106
9-8	286	286	211	211	284	284	106	106
9-9	286	286	209	209	284	284	106	106
9-10	286	286	206	206	284	284	103	103
9-11	284	284	209	209	284	284	106	106
9-12	284	284	206	206	284	284	103	103
10-1	282	282	206	206	284	284	106	106
10-2	284	284	209	209	284	284	106	106
10-3	286	286	206	206	284	284	103	103
10-4	284	284	203	203	284	284	106	106
10-5	286	286	206	206	284	284	103	103
10-6	284	284	211	211	284	284	103	103
10-7	286	286	206	206	284	284	103	103
10-8	286	286	206	206	284	284	103	103
10-9	284	284	211	211	284	284	103	103
10-10	286	286	209	209	284	284	103	103
10-11	284	284	209	209	284	284	103	103

10-12	284	284	209	209	284	284	103	103
10-13	284	284	206	206	284	284	103	103
7561-10b	286	286	197	197	284	284	103	103
7561-11b	286	286	197	197	284	284	103	103
7561-12b	286	286	197	197	284	284	103	103
7561-1a	280	280	197	197	284	284	103	103
7561-2a	286	286	197	197	284	284	103	103
7561-3a	286	286	197	197	284	284	103	103
7561-4a	286	286	197	197	284	284	103	103
7561-5a	286	286	197	197	284	284	103	103
7561-6a	286	286	197	197	284	284	103	103
7561-7b	286	286	197	197	284	284	103	103
7561-8b	286	286	197	197	284	284	103	103
7561-9b	288	288	197	197	284	284	103	106
7566-1	286	286	197	197	284	284	103	103
7566-10	286	286	203	203	284	284	103	103
7566-2	286	286	197	197	284	284	103	103
7566-3	286	286	197	197	284	284	103	103
7566-4	288	288	206	206	284	284	103	103
7566-5	288	288	206	206	284	284	103	103
7566-6	286	286	206	206	284	284	103	103
7566-8	286	286	203	203	284	284	103	103
7566a-11	286	286	209	209	284	284	100	100
7566a-12	286	286	197	197	284	284	103	103
7566a-7	286	286	203	203	284	284	103	103
7566a-9	286	286	203	203	284	284	103	103
7568-10	286	286	206	206	284	284	103	103
7568-11	286	286	206	206	284	284	103	103
7568-12	286	286	206	206	284	284	103	103
7568-13	286	286	206	206	284	284	106	106
7568-1i	286	286	206	206	284	284	103	103
7568-1ii	286	286	209	209	284	284	106	106
7568-2	286	286	209	209	284	284	106	106
7568-3	286	286	206	206	284	284	106	106
7568-4	286	286	206	206	284	284	103	103
7568-5	286	286	197	197	284	284	103	103
7568-6	284	284	206	206	284	284	106	106
7568-7	286	286	206	206	284	284	103	103
7568-8	288	288	209	209	284	284	103	103
7568-9	286	286	206	206	284	284	103	103
7569-1	284	284	206	206	284	284	106	106
7569-10	284	284	206	206	284	284	106	106
7569-11	284	284	206	206	284	284	106	106
7569-12	284	284	206	206	284	284	106	106
7569-13	284	284	206	206	284	284	106	106
7569-2	284	284	206	206	284	284	106	106
**7569-3**	**284**	**284**	**206**	**206**	**284**	**284**	**103**	**103**
7569-4	284	284	206	206	284	284	106	106
7569-5	284	284	206	206	284	284	106	106
7569-6	284	284	206	206	284	284	106	106
7569-8	284	284	206	206	284	284	106	106
7569-9	284	284	206	206	284	284	106	106
7570-1	290	290	206	206	284	284	103	103
7570-10	286	286	206	206	284	284	106	106
7570-11	286	286	206	206	284	284	106	106
7570-12	286	286	206	206	284	284	106	106
7570-13	284	284	206	206	284	284	103	103
7570-2	286	286	206	206	284	284	103	103
7570-3	286	286	206	206	284	284	106	106
7570-4	284	284	206	206	284	284	106	106
7570-5	290	290	206	206	284	284	103	103
7570-6	286	286	206	206	284	284	106	106
7570-7	284	284	206	206	284	284	106	106
7570-8	290	290	206	206	284	284	100	100

7570-9	286	286	206	206	284	284	106	106
7571-10	286	286	209	209	284	284	103	103
7571-12	286	286	206	206	284	284	103	103
7571-13	284	284	209	209	284	284	106	106
7571-1a	286	286	206	206	284	284	106	106
7571-2	286	286	206	206	284	284	103	103
7571-3	282	282	209	209	284	284	106	106
7571-4	282	282	206	206	284	284	106	106
7571-5	284	284	206	206	284	284	103	103
7571-6	286	286	206	206	284	284	103	103
7571-7	286	286	203	203	284	284	106	106
7571-9	282	282	206	206	284	284	106	106
7575-1	286	286	206	206	284	284	103	103
7575-2	284	284	206	206	284	284	103	103
7575-9	284	284	206	206	284	284	103	103
7575a-10	284	284	206	206	284	284	103	103
7575a-11	286	286	206	206	284	284	103	103
7575a-12	284	284	206	206	284	284	103	103
7575a-13	284	284	206	206	284	284	103	103
7575a-3	284	284	206	206	284	284	103	103
7575a-4	284	284	206	206	284	284	103	103
7575a-5	286	286	206	206	284	284	103	103
7575a-6	284	284	206	206	284	284	103	103
7575a-7	284	284	206	206	284	284	103	103
7577-1	286	286	209	209	284	284	106	106
7577-10	286	286	209	209	284	284	106	106
7577-11	286	286	206	206	284	284	103	103
7577-2	286	286	209	209	284	284	106	106
7577-3	286	286	211	211	284	284	106	106
7577-4	284	284	209	209	284	284	103	103
7577-5	286	286	211	211	284	284	106	106
7577-6	284	284	211	211	284	284	106	106
7577-7	286	286	211	211	284	284	106	106
7577-8	286	286	211	211	284	284	106	106
7577-9	286	286	203	203	284	284	106	106
7581-10	286	286	197	197	284	284	110	110
7581-11	286	286	206	206	284	284	103	103
7581-12	286	286	206	206	284	284	103	103
7581-2	282	282	206	206	284	284	103	103
7581-3	280	280	206	206	284	284	106	106
7581-4	282	282	206	206	284	284	106	106
7581-5	286	286	206	206	284	284	106	106
7581-6	286	286	206	206	284	284	103	103
7581-7	286	286	209	209	284	284	106	106
7581-8	286	286	206	206	284	284	106	106
7581-9	286	286	209	209	284	284	103	103
7583-1	284	284	206	206	284	284	106	106
7583-10	284	284	206	206	284	284	103	103
7583-11	284	284	209	209	284	284	103	103
7583-12	284	284	206	206	284	284	103	103
7583-2	284	284	209	209	284	284	106	106
7583-3	284	284	206	206	284	284	106	106
7583-4	284	284	206	206	284	284	103	103
7583-5	284	284	206	206	284	284	103	103
7583-6	284	284	206	206	284	284	103	103
7583-7	284	284	206	206	284	284	103	103
7583-8	284	284	206	206	284	284	103	103
7583-9	284	284	206	206	284	284	103	103
7584-1	284	284	203	203	284	284	103	103
7584-10	286	286	206	206	284	284	103	103
7584-11	290	290	203	203	284	284	103	103
7584-12	290	290	203	203	284	284	103	103
7584-2	290	290	206	206	284	284	103	103
7584-3	286	286	209	209	284	284	103	103

7584-4	286	286	206	206	284	284	103	103
7584-5	290	290	206	206	284	284	106	106
7584-6	288	288	203	203	284	284	103	103
7584-7	286	286	206	206	284	284	103	103
7584-8	286	286	206	206	284	284	106	106
7584-9	286	286	206	206	284	284	103	103
7586-1	290	290	206	206	284	284	103	103
7586-10	284	284	206	206	284	284	103	103
7586-11	290	290	206	206	284	284	103	103
7586-12	290	290	206	206	284	284	103	103
7586-2	290	290	209	209	284	284	103	103
7586-3	284	284	206	206	284	284	106	106
7586-4	290	290	206	206	284	284	103	103
7586-5	286	286	206	206	284	284	103	103
7586-6	290	290	206	206	284	284	103	103
7586-7	286	286	206	206	284	284	103	103
7586-8	290	290	206	206	284	284	103	103
7586-9	290	290	206	206	284	284	103	103
7588-10	284	284	211	211	284	284	103	103
7588-11	286	286	211	211	284	284	106	106
7588-12	286	286	211	211	284	284	106	106
7588-14	288	288	206	206	284	284	106	106
7588-2	284	284	211	211	284	284	103	103
7588-3	286	286	206	206	284	284	103	103
7588-4	290	290	209	209	284	284	103	103
7588-5	290	290	206	206	284	284	103	103
7588-6	290	290	206	206	284	284	103	103
7588-7	284	284	211	211	284	284	106	106
7588-8	288	288	206	206	284	284	106	106
7588-9	286	286	209	209	284	284	103	103
7594-1	290	290	206	206	284	284	103	103
7594-10	284	284	206	206	284	284	103	103
7594-11	284	284	206	206	284	284	103	103
7594-12	290	290	206	206	284	284	103	103
7594-2	290	290			284	284	103	103
7594-3	286	286	206	206	284	284	103	103
7594-4	290	290	197	197	284	284	103	103
7594-5	286	286	206	206	284	284	103	103
7594-6	290	290	206	206	284	284	103	103
7594-7	290	290	209	209	284	284	103	103
7594-8	286	286	206	206	284	284	103	103
7594-9	294	294	211	211	284	284	106	106
7595-1	284	284	197	197	284	284	106	106
7595-10	284	284	197	197	284	284	106	106
7595-11	288	288	206	206	284	284	103	103
7595-12	288	288	206	206	284	284	103	103
7595-13	284	284	197	197	284	284	106	106
7595-3	284	284	197	197	284	284	106	106
7595-4	284	284	197	197	284	284	106	106
7595-5	282	282	206	206	284	284	103	103
7595-6	284	284	197	197	284	284	106	106
7595-7	284	284	203	203	284	284	106	106
7595-8	284	284	203	203	284	284	106	106
7595-9	290	290	209	209	284	284	103	103
7596-1	286	286	197	197	284	284	106	106
7596-10	286	286	206	206	284	284	103	103
7596-11	286	286	203	203	284	284	103	103
7596-12	286	286	206	206	284	284	103	103
7596-2	286	286	206	206	284	284	103	103
7596-3	284	284	203	203	284	284	103	103
7596-4	280	280	203	203	284	284	103	103
7596-5	284	284	203	203	284	284	103	103
7596-7	284	284	206	206	284	284	103	103
7596-8	286	286	197	197	284	284	103	103

7596-9	286	286	209	209	284	284	103	103
7599-1	284	284	209	209	284	284	106	106
7599-10	284	284	206	206	284	284	103	103
7599-11	286	286	206	206	284	284	106	106
7599-12	284	284	206	206	284	284	106	106
7599-13	288	288	206	206	284	284	103	103
7599-3	284	284	206	206	284	284	106	106
7599-4	286	286	206	206	284	284	106	106
7599-5	284	284	206	206	284	284	106	106
7599-6	286	286	206	206	284	284	103	103
7599-7	286	286	206	206	284	284	106	106
7599-8	286	286	206	206	284	284	106	106
7599-9	286	286	206	206	284	284	106	106
7600-1	284	284	206	206	284	284	106	106
7600-10	284	284	203	203	284	284	103	103
7600-11	286	286	197	197	284	284	103	103
7600-2	284	284	211	211	284	284	103	103
7600-3	284	284	209	209	284	284	103	103
7600-4	284	284	211	211	284	284	103	103
7600-5	284	284	209	209	284	284	103	103
7600-6	284	284	209	209	284	284	103	103
7600-7	286	286	206	206	284	284	103	103
7600-8	284	284	206	206	284	284	103	103
7600-9	284	284	206	206	284	284	103	103
7602-10	290	290	206	206	284	284	100	100
7602-11	284	284	206	206				
7602-12	286	286	206	206	284	284	100	100
7602-2	286	286	206	206	284	284	103	103
7602-4	286	286	197	197	284	284	103	103
7602-5	286	286	206	206	284	284	103	103
7602-6	286	286	206	206	284	284	103	103
7602-7	286	286	206	206	284	284	103	103
7602-9	286	286	206	206	284	284	103	103
7604-1	286	286	206	206	284	284	103	103
7604-10	286	286	211	211	284	284	103	103
7604-11	286	286	206	206	284	284	103	103
7604-12	286	286	211	211	284	284	103	103
7604-13	286	286	206	206	284	284	103	103
7604-2	286	286	206	206	284	284	103	103
7604-3	286	286	206	206	284	284	103	103
7604-4	286	286	211	211	284	284	103	103
7604-6	286	286	211	211	284	284	103	103
7604-7	280	280	209	209	284	284	103	103
7604-8	286	286	206	206	284	284	103	103
7604-9	286	286	206	206	284	284	103	103
7606-1	288	288	203	203	284	284	103	103
7606-10	286	286	206	206	284	284	103	103
7606-11	280	280	209	209	284	284	103	103
7606-12	280	280	209	209	284	284	103	103
7606-3	286	286	206	206	284	284	103	103
7606-4	284	284	206	206	284	284	103	103
7606-5	284	284	206	206	284	284	103	103
7606-6	284	284	206	206	284	284	103	103
7606-7	286	286	206	206	284	284	103	103
7606-8	286	286	206	206	284	284	103	103
7606-9	280	280	209	209	284	284	103	103
7610-1	290	290	206	206	284	284	106	106
7610-10	284	284	206	206	284	284	103	103
7610-11	288	288	209	209	284	284	103	103
7610-12	284	284	206	206	284	284	103	103
7610-2	284	284	206	206	284	284	103	103
7610-3	286	286	206	206	284	284	103	103
7610-4	286	286	206	206	284	284	103	103
7610-5	284	284	206	206	284	284	103	103

7610-6	284	284	206	206	284	284	103	103
7610-7	286	286	206	206	284	284	103	103
7610-8	284	284	206	206	284	284	103	103
7610-9	290	290	209	209	284	284	103	103
7612-1	288	288	197	197	284	284	106	106
7612-10	288	288	197	197	284	284	106	106
7612-11	286	286	206	206	284	284	103	103
7612-12	290	290	206	206	284	284	103	103
7612-13	288	288	197	197	284	284	106	106
7612-2	288	288	206	206	284	284	103	103
7612-3	288	288	197	197	284	284	106	106
7612-4	286	286	206	206	284	284	103	103
7612-5	286	286	206	206	284	284	103	103
7612-6	286	286	206	206	284	284	103	103
7612-8	288	288	203	203	284	284	106	106
7612-9	290	290	206	206	284	284	103	103
7616-1	286	286	206	206	284	284	103	103
7616-10	286	286	206	206	284	284	103	103
7616-13	286	286	206	206	284	284	103	103
7616-14	286	286	206	206	284	284	100	100
7616-1ii	284	284	206	206	284	284	106	106
7616-2	286	286	206	206	284	284	103	103
7616-3	284	284	206	206	284	284	106	106
7616-4	286	286	206	206	284	284	103	103
7616-5	286	286	206	206	284	284	103	103
7616-6	286	286	206	206	284	284	103	103
7616-7	284	284	206	206	284	284	103	103
7616-8	290	290	206	206	284	284	103	103
7616-9i	286	286	206	206	284	284	103	103
7625-1	286	286	206	206	284	284	106	106
7625-10	286	286	206	206	284	284	106	106
7625-11	284	284	206	206	284	284	103	103
7625-2	286	286	206	206	284	284	106	106
7625-4	286	286	206	206	284	284	106	106
7625-5	286	286	206	206	284	284	106	106
7625-7	286	286	206	206	284	284	106	106
7625-8	286	286	206	206	284	284	106	106
7628-1	286	286	206	206	284	284	106	106
7628-10	286	286	206	206	284	284	106	106
7628-11	286	286	206	206	284	284	106	106
7628-12	286	286	206	206	284	284	106	106
7628-2	286	286	206	206	284	284	106	106
7628-3	286	286	206	206	284	284	106	106
7628-4	286	286	206	206	284	284	106	106
7628-5	286	286	206	206	284	284	106	106
7628-6	286	286	206	206	284	284	103	103
7628-7	284	284	209	209	284	284	106	106
7628-8	286	286	206	206	284	284	106	106
7628-9	286	286	206	206	284	284	106	106
7640-1	286	286	209	209	284	284	103	103
7640-11	282	282	206	206	284	284	103	103
7640-12	286	286	203	203	284	284	103	103
7640-2	282	282	206	206	284	284	103	103
7640-3	286	286	203	203	284	284	106	106
7640-4	284	284	209	209	284	284	106	106
7640-5	282	282	206	206	284	284	106	106
7640-6	282	282	209	209	284	284	106	106
7640-7	282	282	206	206	284	284	103	103
7640-8	282	282	206	206	284	284	103	103
7640-9	282	282	206	206	284	284	103	103

Individual	GBM1064	GBM1064	GBM1035	GBM1035	GBM1003	GBM1003	GBM1334	GBM1334
1-2	288	288	272	272	195	195	118	118
1-3	288	288	274	274	195	195	129	129
1-4	288	288	278	278	195	195	129	129
1-6	288	288	274	274	195	195	129	129
1-7	288	288	272	272	198	198	129	129
1-8	288	288	280	280	195	195	126	126
1-9	288	288	274	274	195	195	129	129
1-10	288	288	278	278	204	204	129	129
1-11	288	288	276	276	201	201	129	129
1-12	288	288	274	274	213	213	129	129
1-13	288	288	274	274	195	195	129	129
4-1	288	288	274	274	207	207	129	129
4-3	288	288	276	276	207	207	129	129
4-4	288	288	276	276	195	195	129	129
4-5	288	288	276	276	195	195	129	129
4-6	288	288	276	276	207	207	129	129
4-7	288	288	276	276	210	210	129	129
4-8	288	288	276	276	195	195	129	129
4-9	294	294	276	276	198	198	129	129
4-10	294	294	276	276	198	198	129	129
4-11	294	294	276	276	198	198	129	129
4-12	294	294	276	276	195	195	129	129
4-13	294	294	274	274	198	198	129	129
7-1	297	297	276	276	195	195	129	129
7-2	297	297	276	276	195	195	129	129
7-3	297	297	276	276	195	195	129	129
7-4	297	297	276	276	195	195	129	129
7-5	288	294	280	280	210	210	129	129
7-6	288	288	280	280	210	210	129	129
7-8	297	297	276	276	195	195	129	129
7-9	288	288	274	274	210	210	129	129
7-10	297	297	276	276	195	195	129	129
7-11	297	297	276	276	195	195	129	129
7-12	297	297	276	276	195	195	129	129
9-1	280	280	274	274	204	204	126	126
9-2	288	288	274	274	201	201	118	118
9-3	288	288	274	274	204	204	126	126
9-4	288	288	274	274	204	204	126	126
9-5	288	288	274	274	204	204	126	126
9-6	288	288	274	274	201	201	126	126
9-7	288	288	274	274	204	204	126	126
9-8	288	288	274	274	204	204	126	126
9-9	288	288	274	274	207	207	126	126
9-10	288	288	274	274	201	201	126	126
9-11	288	288	274	274	204	204	126	126
9-12	288	288	274	274	204	204	126	126
10-1	288	288	274	274	204	204	118	118
10-2	280	280	274	280	195	195	126	126
10-3	288	288	278	278	204	204	126	126
10-4	288	288	274	274	204	204	118	118
10-5	280	280	274	280	204	204	126	126
10-6	288	288	276	276	187	187	126	126
10-7	288	288	276	276	195	195	126	126
10-8	288	288	276	276	195	195	126	126
10-9	288	288	278	278	204	204	129	129
10-10	288	288	276	276	195	195	126	126
10-11	288	288	278	278	204	204	129	129

10-12	288	288	276	276	195	195	126	126
10-13	288	288	278	278	198	198	129	129
7561-10b	280	280	276	276	207	207	129	129
7561-11b	280	280	276	276	207	207	129	129
7561-12b	280	280	276	276	207	207	129	129
7561-1a	280	280	274	274	210	210	129	129
7561-2a	280	280	274	274	210	210	129	129
7561-3a	280	280	274	274	207	207	129	129
7561-4a	280	280	276	276	210	210	129	129
7561-5a	280	280	274	274	210	210	129	129
7561-6a	280	280	276	276	210	210	129	129
7561-7b	280	280	274	274	210	210	129	129
7561-8b	280	280	274	274	210	210	129	129
7561-9b	280	280	276	276	207	207	129	129
7566-1	280	280	278	278	210	210	129	129
7566-10	280	280	280	280	195	195	126	126
7566-2	280	280	278	278	210	210	129	129
7566-3	280	280	278	278	207	207	129	129
7566-4	280	280	274	274	210	210	129	129
7566-5	280	280	274	280	210	210	129	129
7566-6	280	280	276	276	201	201	129	129
7566-8	280	280	280	280	195	195	126	126
7566a-11	280	280	276	276	210	210	126	126
7566a-12	280	280	278	278	210	210	129	129
7566a-7	280	280	280	280	195	195	126	126
7566a-9	280	280	280	280	195	195	126	126
7568-10	280	280	280	280	207	207	129	129
7568-11	280	280	280	280	207	207	129	129
7568-12	280	280	280	280	207	207	129	129
7568-13	288	288	274	274	204	204	126	126
7568-1i	280	280	280	280	207	207	129	129
7568-1ii	294	294	274	274	201	201	118	118
7568-2	294	294	274	274	201	201	118	118
7568-3	294	294	276	276	195	195	129	129
7568-4	280	280	280	280	207	207	129	129
7568-5	280	280	276	276	207	207	129	129
7568-6	280	280	274	274	201	201	129	129
7568-7	280	280	280	280	207	207	129	129
7568-8	280	280	280	280	195	195	126	126
7568-9	280	280	280	280	207	207	129	129
7569-1	288	288	278	278	195	195	157	157
7569-10	288	288	276	276	195	195	157	157
7569-11	288	288	276	276	195	195	157	157
7569-12	288	288	278	278	195	195	157	157
7569-13	288	288	276	276	195	195	157	157
7569-2	288	288	278	278	195	195	157	157
**7569-3**	**288**	**288**	**274**	**274**	**201**	**201**	**129**	**129**
7569-4	288	288	276	276	195	195	157	157
7569-5	288	288	276	276	195	195	157	157
7569-6	288	288	276	276	195	195	157	157
7569-8	288	288	276	276	195	195	157	157
7569-9	288	288	276	276	195	195	157	157
7570-1	280	280	280	280	201	201	129	129
7570-10	280	280	272	280	195	195	126	126
7570-11	280	288	272	272	195	195	126	126
7570-12	280	280	274	274	195	195	126	126
7570-13	297	297	274	274	187	187	129	129
7570-2	280	280	278	278	187	187	118	118
7570-3	280	280	272	280	195	195	126	126
7570-4	280	280	276	276	201	201	118	118
7570-5	280	280	278	278	201	201	129	129
7570-6	280	280	272	280	195	195	126	126
7570-7	280	280	276	276	201	201	118	118
7570-8	280	280	272	280	195	195	129	129

7570-9	280	280	272	280	195	195	126	126
7571-10	288	288	272	272	204	204	129	129
7571-12	280	280	274	274	201	201	129	129
7571-13	288	288	274	274	201	201	118	118
7571-1a	280	280	276	276	198	198	129	129
7571-2	288	288	272	272	201	201	126	126
7571-3	288	288	274	274	201	201	118	118
7571-4	280	280	276	276	195	195	129	129
7571-5	288	288	276	276	187	187	118	118
7571-6	288	288	274	274	201	201	126	146
7571-7	294	294	272	272	201	201	126	126
7571-9	288	288	276	276	201	201	118	118
7575-1	288	288	276	276	195	195	129	129
7575-2	288	288	276	276	195	195	129	129
7575-9	288	288	276	276	195	195	129	129
7575a-10	288	288	276	276	195	195	129	129
7575a-11	288	288	276	276	195	195	129	129
7575a-12	288	288	276	276	195	195	129	129
7575a-13	288	288	276	276	195	195	129	129
7575a-3	288	288	276	276	195	195	129	129
7575a-4	288	288	276	276	195	195	129	129
7575a-5	288	288	276	276	195	195	129	129
7575a-6	288	288	276	276	195	195	129	129
7575a-7	288	288	276	276	195	195	129	129
7577-1	288	288	278	278	207	207	118	118
7577-10	288	288	278	278	207	207	118	118
7577-11	288	288	274	274	207	207	126	126
7577-2	288	288	278	278	207	207	118	118
7577-3	288	288	278	278	207	207	118	118
7577-4	288	288	276	276	207	207	126	126
7577-5	288	288	276	276	201	201	118	118
7577-6	288	288	276	276	207	207	126	126
7577-7	288	288	278	278	207	207	118	118
7577-8	288	288	278	278	207	207	118	118
7577-9	288	288	278	278	204	204	118	118
7581-10	280	280	274	274	195	195	126	126
7581-11	288	288	274	274	187	187	126	126
7581-12	288	288	274	274	187	187	126	126
7581-2	288	288	274	274	201	201	126	126
7581-3	288	288	274	274	201	201	126	126
7581-4	288	288	274	274	195	195	126	126
7581-5	288	288	274	274	195	195	126	126
7581-6	288	288	274	274	195	195	129	129
7581-7	288	288	278	278	204	204	126	126
7581-8	288	288	274	274	195	195	126	126
7581-9	288	288	274	274	195	195	126	126
7583-1	288	288	278	278	195	195	126	126
7583-10	280	280	278	278	195	195	118	118
7583-11	280	280	278	278	195	195	118	118
7583-12	288	288	274	274	195	195	126	126
7583-2	288	288	278	278	195	195	126	126
7583-3	280	280	278	278	195	195	118	118
7583-4	280	280	278	278	195	195	118	118
7583-5	280	280	278	278	195	195	118	118
7583-6	280	280	278	278	195	195	118	118
7583-7	280	280	280	280	195	195	118	118
7583-8	280	280	278	278	195	195	118	118
7583-9	280	280	278	278	195	195	118	118
7584-1	285	285	274	274	213	213	118	118
7584-10	288	288	274	274	195	195	129	129
7584-11	280	280	276	276	195	195	118	118
7584-12	285	285	274	274	195	195	118	118
7584-2	285	285	274	274	201	201	118	118
7584-3	285	285	276	276	204	204	126	126

7584-4	288	288	274	274	195	195	126	126
7584-5	288	288	272	272	195	195	126	126
7584-6	285	285	274	274	195	195	129	129
7584-7	280	280	274	274	195	195	118	118
7584-8	288	288	272	272	195	195	126	126
7584-9	285	285	274	274	204	204	126	126
7586-1	288	288	274	274	187	187	126	126
7586-10	288	288	274	274	187	187	126	126
7586-11	288	288	274	274	187	187	126	126
7586-12	288	288	274	274	187	187	126	126
7586-2	288	288	274	274	195	195	118	118
7586-3	288	288	274	274	187	187	126	126
7586-4	288	288	274	274	187	187	126	126
7586-5	288	288	274	274	201	201	118	118
7586-6	288	288	274	274	187	187	126	126
7586-7	288	288	278	278	201	201	129	129
7586-8	288	288	274	274	187	187	126	126
7586-9	288	288	274	274	187	187	126	126
7588-10	288	288	282	282	195	195	118	118
7588-11	280	280	282	282	201	201	129	129
7588-12	280	280	280	280	201	201	129	129
7588-14	280	280	274	274	201	201	126	126
7588-2	288	288	282	282	195	195	118	118
7588-3	280	280	280	280	201	201	118	118
7588-4	280	280	274	274	201	201	126	126
7588-5	280	280	274	274	201	201	126	126
7588-6	280	280	274	274	201	201	126	126
7588-7	280	280	274	274	201	201	126	126
7588-8	280	280	274	274	195	195	129	129
7588-9	280	280	274	280	195	195	118	118
7594-1	280	280	274	274	204	204	129	129
7594-10	280	280	276	276	195	195	129	129
7594-11	280	280	274	274	195	195	129	129
7594-12	280	280	274	274	195	195	129	129
7594-2	280	280	274	274	195	195	126	126
7594-3	280	280	276	276	201	201	129	129
7594-4	297	297	274	274	195	195	129	129
7594-5	280	280	274	274	195	195	126	126
7594-6	280	280	274	274	195	195	126	126
7594-7	280	280	274	274	195	195	126	126
7594-8	280	280	274	274	195	195	129	129
7594-9	294	294	274	274	204	204	129	129
7595-1	288	288	274	274	198	198	126	126
7595-10	288	288	276	276	195	195	126	126
7595-11	288	288	276	276	204	204	129	129
7595-12	288	288	276	276	204	204	129	129
7595-13	288	288	274	274	195	195	126	126
7595-3	288	288	274	274	195	195	126	126
7595-4	288	288	274	274	195	195	126	126
7595-5	288	288	274	274	195	195	126	126
7595-6	288	288	276	276	195	195	126	126
7595-7	288	288	274	274	204	204	129	129
7595-8	288	288	274	274	204	204	126	126
7595-9	280	280	272	272	195	195	126	126
7596-1	297	297	274	274	195	195	129	129
7596-10	280	280	274	274	195	195	129	146
7596-11	280	280	274	274	195	195	129	129
7596-12	280	280	280	280	195	195	118	118
7596-2	288	288	274	274	195	195	118	118
7596-3	280	280	274	274	195	195	129	129
7596-4	288	288	280	280	195	195	129	129
7596-5	288	288	274	274	195	195	129	129
7596-7	280	280	274	274	195	195	118	118
7596-8	288	288	276	276	187	187	126	126

7596-9	288	288	276	276	195	195		
7599-1	280	280	276	276	195	195	118	118
7599-10	280	280	274	274	201	201	118	118
7599-11	294	294	276	276	195	195	118	118
7599-12	280	280	276	276	195	195	118	118
7599-13	280	280	272	272	201	201	118	118
7599-3	280	280	276	276	195	195	118	118
7599-4	280	280	274	274	223	223	126	126
7599-5	280	280	280	280	195	195	118	118
7599-6	280	280	274	274	201	201	129	129
7599-7	294	294	276	276	195	195	118	118
7599-8	280	280	276	276	204	204	126	126
7599-9	280	280	274	274	204	204	129	129
7600-1	288	288	274	274	195	195	118	118
7600-10	288	288	276	276			129	129
7600-11	280	280	274	274	210	210	129	129
7600-2	288	288	274	274	204	204	129	129
7600-3	288	288	274	274	204	204	129	129
7600-4	288	288	274	274	204	204	129	129
7600-5	288	288	274	274	201	201	129	129
7600-6	288	288	274	274	201	201	129	129
7600-7	288	288	274	274	201	201	129	129
7600-8	288	288	274	274	207	207	129	129
7600-9	288	288	274	274	207	207	129	129
7602-10	280	280	274	274	207	207	129	129
7602-11	280	280	274	274	210	210	129	129
7602-12	280	280	276	276	195	195	129	129
7602-2	288	288	274	274	201	201	129	129
7602-4	280	280	274	280	210	210	129	129
7602-5	280	280	276	276	201	201	129	129
7602-6	280	280	276	276	210	210	129	129
7602-7	280	280	274	274	207	207	118	118
7602-9	288	288	274	274	207	207	129	129
7604-1	280	280	278	278	201	201	129	129
7604-10	288	288	278	278	201	201	129	129
7604-11	280	280	276	276	207	207	129	129
7604-12	280	280	280	280	207	207	129	129
7604-13	280	280	274	274	207	207	129	129
7604-2	280	280	276	276	207	207	129	129
7604-3	280	280	276	276	207	207	129	129
7604-4	280	280	276	276	210	210	129	129
7604-6	280	280	274	274	210	210	129	129
7604-7	280	280	274	274	210	210	129	129
7604-8	288	288	278	278	207	207	129	129
7604-9	280	280	276	276	207	207	129	129
7606-1	288	288	276	276	195	195	129	129
7606-10	280	280	278	278	195	195	129	129
7606-11	288	288	272	272	195	195	118	118
7606-12	288	288	272	272	195	195	118	118
7606-3	288	288	274	274	195	195	129	129
7606-4	288	288	282	282	195	195	129	129
7606-5	285	285	274	274	195	195	129	129
7606-6	285	285	274	274	195	195	129	129
7606-7	280	280	276	276	210	210	129	129
7606-8	280	280	276	276	210	210	129	129
7606-9	288	288	272	272	195	195	118	118
7610-1	288	288	274	274	201	201	129	129
7610-10	280	280	274	274	195	195	129	129
7610-11	288	288	274	274	195	195	129	129
7610-12	280	280	274	274	195	195	129	129
7610-2	288	288	274	274	195	195	129	129
7610-3	280	280	274	274	195	195	126	126
7610-4	280	280	274	274	195	195	118	118
7610-5	288	288	280	280	195	195	129	129

7610-6	288	288	280	280	195	195	129	129
7610-7	288	288	280	280	195	195	129	129
7610-8	288	288	280	280	195	195	129	129
7610-9	288	288	274	274	195	195	118	118
7612-1	280	280	274	274	195	195	129	129
7612-10	280	280	274	274	195	195	129	129
7612-11	280	280	274	274	207	207	129	129
7612-12	280	280	276	276	210	210	129	129
7612-13	280	280	274	274	195	195	129	129
7612-2	280	280	276	276	195	195	129	129
7612-3	280	280	274	274	195	195	129	129
7612-4	280	280	276	276	210	210	129	129
7612-5	280	280	276	276	207	207	129	129
7612-6	280	280	276	276	207	207	129	129
7612-8	288	288	276	276	195	195	129	129
7612-9	280	280	276	276	207	207	129	129
7616-1	280	280	278	278	195	195	129	129
7616-10	280	280	278	278	195	195	129	129
7616-13	280	280	278	278	195	195	129	129
7616-14	280	280	278	278	210	210	129	129
7616-1ii	288	288	274	274	195	195	118	118
7616-2	280	280	278	278	195	195	129	129
7616-3	280	280	274	274	213	213	126	126
7616-4	280	280	278	278	195	195	129	129
7616-5	280	280	278	278	195	195	129	129
7616-6	280	280	278	278	195	195	129	129
7616-7	280	280	276	276	207	207	129	129
7616-8	285	285	274	274	195	195	126	126
7616-9i	280	280	278	278	195	195	129	129
7625-1	294	294	276	276	210	210	129	129
7625-10	294	294	274	274	207	207	129	129
7625-11	288	288	274	274	195	195	129	129
7625-2	294	294	274	274	207	207	129	129
7625-4	294	294	274	274	207	207	129	129
7625-5	294	294	276	276	210	210	129	129
7625-7	294	294	276	276	210	210	129	129
7625-8	294	294	276	276	207	207	129	129
7628-1	288	288	280	280	210	210	129	129
7628-10	288	288	280	280	210	210	129	129
7628-11	288	288	280	280	207	207	129	129
7628-12	288	288	276	276	204	204	129	129
7628-2	297	297	276	276	195	195	129	129
7628-3	288	288	276	276	210	210	129	129
7628-4	288	288	276	276	207	207	129	129
7628-5	288	288	280	280	210	210	129	129
7628-6	288	288	276	276	207	207	129	129
7628-7	280	280	274	280	201	201	129	129
7628-8	288	288	280	280	207	207	129	129
7628-9	288	288	280	280	207	207	129	129
7640-1	280	280	274	280	201	201	129	129
7640-11	288	288	276	276	195	195	129	129
7640-12	280	280	280	280	207	207	129	129
7640-2	280	280	274	274	201	201	129	129
7640-3	280	280	280	280	195	195	129	129
7640-4	297	297	272	272	187	187	129	129
7640-5	288	288	274	274	201	201	129	129
7640-6	297	297	274	274	201	201	129	129
7640-7	297	297	274	274	195	195	129	129
7640-8	280	280	274	274	201	201	157	157
7640-9	280	280	274	274	201	201	129	157

Individual	GBM1036	GBM1036	GBM1020	GBM1020	GBM1029	GBM1029	GBM1047	GBM1047
1-2	149	149	246	246	225	225	213	213
1-3	147	147	246	246	225	225	205	205
1-4	149	149	248	248	225	225	205	205
1-6	147	147	246	246	225	225	205	205
1-7	151	151	248	248	225	225	205	205
1-8	147	147	246	246	225	225	205	205
1-9	149	149	248	248	225	225	205	205
1-10	149	149	248	248	225	225	213	213
1-11	149	149	246	246	225	225	213	213
1-12	147	147	246	246	225	225	205	205
1-13	149	149	246	246	225	225	205	205
4-1	149	149	246	246	225	225	209	209
4-3	149	149	246	246	225	225	209	209
4-4	149	149	246	246	225	225	211	211
4-5	149	149	246	246	225	225	209	209
4-6	149	149	246	246	225	225	211	211
4-7	147	147	244	244	225	225	211	211
4-8	149	149	246	246	225	225	209	209
4-9	149	149	244	244	225	225	211	211
4-10	149	149	244	244	225	225	209	209
4-11	149	149	246	246	225	225	209	209
4-12	149	149	246	246	225	225		
4-13	149	149	244	244	225	225	211	211
7-1	149	149	244	244	229	229	211	211
7-2	149	149	244	244	229	229	211	211
7-3	149	149	244	244	229	229	209	209
7-4	149	149	244	244	229	229	209	209
7-5	149	149	244	244	229	229	211	211
7-6	149	149	244	244	229	229	211	211
7-8	149	149	246	246	225	225	211	211
7-9	147	147	244	244	231	231	211	211
7-10	149	149	246	246	225	225	211	211
7-11	149	149	246	246	225	225	209	209
7-12	147	147	244	244	225	225	209	209
9-1	149	149	244	244	225	225	211	211
9-2	149	149	246	246	225	225	211	211
9-3	151	151	246	246	225	225	211	211
9-4	151	151	248	248	225	225	211	211
9-5	151	151	246	246	225	225	211	211
9-6	151	151	248	248	225	225	211	211
9-7	149	149	246	246	225	225	211	211
9-8	149	149	248	248	225	225	211	211
9-9	151	151	244	244	225	225	211	211
9-10	151	151	246	246	225	225	211	211
9-11	149	149	244	244	225	225	211	211
9-12	151	151	246	246	225	225	211	211
10-1	149	149	248	248	227	227	211	211
10-2	149	149	246	246	225	225	211	211
10-3	149	149	246	246	225	225	209	209
10-4	149	149	248	248	227	227	209	209
10-5	149	149	244	244	225	225	211	211
10-6	151	151	246	246	225	225	211	211
10-7	151	151	244	244	225	225	211	211
10-8	147	147	238	238	225	225	209	209
10-9	149	149	246	246	225	225	213	213
10-10	147	147	238	238	225	225	211	211
10-11	149	149	244	244	225	225	213	213

10-12	147	147	238	238	225	225	209	209
10-13	149	149	250	250	225	225	209	209
7561-10b	149	149	248	248	233	233	213	213
7561-11b	149	149	248	248	233	233	213	213
7561-12b	149	149	248	248	233	233	213	213
7561-1a	149	149	250	250	233	233	213	213
7561-2a	149	149	248	248	233	233	213	213
7561-3a	149	149	248	248	233	233		
7561-4a	149	149	248	248	233	233	213	213
7561-5a	149	149	248	248	233	233	213	213
7561-6a	149	149	248	248	233	233	213	213
7561-7b	149	149	248	248	233	233	213	213
7561-8b	149	149	248	248	233	233	213	213
7561-9b	149	149	248	250	233	233	213	213
7566-1	149	149	244	244	233	233	213	213
7566-10	149	149	246	246	225	225	213	213
7566-2	149	149	244	244	233	233	213	213
7566-3	149	149	244	244	233	233	213	213
7566-4	145	145	244	244	225	225	213	213
7566-5	147	147	244	244	225	225	213	213
7566-6	151	151	246	246	233	233	211	211
7566-8	147	147	244	244	225	225	213	213
7566a-11	149	149	246	246	233	233	207	207
7566a-12	149	149	244	244	233	233	213	213
7566a-7	149	149	244	244	225	225	215	215
7566a-9	149	149	244	244	225	225	213	213
7568-10	149	149	246	246	225	225	213	213
7568-11	149	149	246	246	225	225	213	213
7568-12	149	149	246	246	225	225	213	213
7568-13	149	149	248	248	227	227	211	211
7568-1i	149	149	246	246	225	225	213	213
7568-1ii	147	147	246	246	225	225	211	211
7568-2	147	147	246	246	225	225	211	211
7568-3	149	149	244	244	229	229	211	211
7568-4	149	149	246	246	225	225	213	213
7568-5	149	149	248	248	233	233	213	213
7568-6	151	151	244	244	225	225		
7568-7	149	149	246	246	225	225	213	213
7568-8	149	149	250	250	225	225	213	213
7568-9	149	149	248	248	225	225	213	213
7569-1	149	149	250	250	225	225	215	215
7569-10	149	149	248	248	225	225	213	213
7569-11	149	149	250	250	225	225	215	215
7569-12	149	149	248	248	225	225	213	213
7569-13	149	149	248	248	225	225	213	213
7569-2	149	149	248	248	225	225	213	213
**7569-3**	**149**	**149**	**244**	**244**	**225**	**225**	**215**	**215**
7569-4	149	149	248	248	225	225	213	213
7569-5	149	149	248	248	225	225	213	213
7569-6	149	149	248	248	225	225		
7569-8	149	149	248	248	225	225	213	213
7569-9	149	149	248	248	225	225		
7570-1	149	149	248	248	225	225	213	213
7570-10	145	145	244	244	225	225	211	211
7570-11	147	147	244	244	225	225	211	211
7570-12	147	147	244	244	225	225	211	211
7570-13	151	151	244	244	225	225	211	211
7570-2	147	147	244	244	225	225	211	211
7570-3	147	147	244	244	225	225	209	209
7570-4	149	149	248	248	225	225	205	205
7570-5	149	149	353	353	225	225	213	213
7570-6	147	147	244	244	225	225	211	211
7570-7	149	149	248	248	225	225	205	205
7570-8	147	147	244	244	225	225	211	211

7570-9	145	145	244	244	225	225	211	211
7571-10	149	149	248	248	225	225	213	213
7571-12	147	147	244	244	225	225	213	213
7571-13	149	149	246	246	225	225	205	205
7571-1a	151	151	250	250	225	225	213	213
7571-2	147	147	248	248	225	225	211	211
7571-3	151	151	246	246	225	225	205	205
7571-4	151	151	246	246	225	225	211	211
7571-5	147	147	246	246	225	225	211	211
7571-6	147	147	244	244	225	225	213	213
7571-7	147	147	246	246	225	225	209	209
7571-9	151	151	246	246	225	225	205	205
7575-1	151	151	246	246	233	233	213	213
7575-2	151	151	248	248	233	233	213	213
7575-9	151	151	248	248	233	233	213	213
7575a-10	145	145	246	246	233	233	215	215
7575a-11	151	151	248	248	233	233	213	213
7575a-12	151	151	248	248	233	233	213	213
7575a-13	151	151	248	248	233	233	213	213
7575a-3	151	151	248	248	233	233	213	213
7575a-4	151	151	248	248	233	233	213	213
7575a-5	151	151	246	246	233	233	215	215
7575a-6	151	151	248	248	233	233	213	213
7575a-7	151	151	248	248	233	233	213	213
7577-1	151	151	246	246	225	225	213	213
7577-10	151	151	246	246	225	225	213	213
7577-11	151	151	248	248	225	225	213	213
7577-2	151	151	246	246	225	225	213	213
7577-3	151	151	246	246	225	225	211	211
7577-4	151	151	248	248	225	225	209	209
7577-5	151	151	244	244	225	225	213	213
7577-6	151	151	244	244	225	225	209	209
7577-7	151	151	246	246	225	225	213	213
7577-8	151	151	244	244	225	225	211	211
7577-9	149	149	250	250	225	225	215	215
7581-10	147	147	246	246	225	225	213	213
7581-11	151	151	248	248	225	225	213	213
7581-12	151	151	248	248	225	225	211	211
7581-2	149	149	244	244	225	225	211	211
7581-3	151	151	244	244	225	225	209	209
7581-4	151	151	244	244	225	225	213	213
7581-5	151	151	246	246	225	225	213	213
7581-6	149	149	246	246	225	225	209	209
7581-7	149	149	248	248	225	225	209	209
7581-8	151	151	246	246	225	225	213	213
7581-9	149	149	244	244	225	225	213	213
7583-1	149	149	246	246	225	225	213	213
7583-10	145	145	242	242	225	225	209	209
7583-11	145	145	244	244	225	225	211	211
7583-12	149	149	244	244	225	225	211	211
7583-2	149	149	246	246	225	225	213	213
7583-3	147	147	244	244	225	225	211	211
7583-4	145	145	242	242	225	225	211	211
7583-5	145	145	242	242	225	225	211	211
7583-6	145	145	242	242	225	225	211	211
7583-7	145	145	242	242	225	225	211	211
7583-8	145	145	242	242	225	225	209	209
7583-9	145	145	242	242	225	225	211	211
7584-1	149	149	248	248	225	225	213	213
7584-10	147	147	248	248	225	225	213	213
7584-11	147	147	244	244	225	225	209	209
7584-12	147	149	244	244	225	225	211	211
7584-2	147	147	244	244	225	225	213	213
7584-3	151	151	246	246	225	225	213	213

7584-4	147	147	250	250	225	225	213	213
7584-5	149	149	242	242	225	225	213	213
7584-6	147	147	244	244	225	225	211	211
7584-7	151	151	244	244	225	225	213	213
7584-8	149	149	244	244	225	225	213	213
7584-9	151	151	246	246	225	225	211	211
7586-1	149	149	246	246	225	225	211	211
7586-10	149	149	250	250	225	225	211	211
7586-11	149	149	246	246	225	225	211	211
7586-12	149	149	246	246	225	225	211	211
7586-2	151	151	250	250	225	225	211	211
7586-3	149	149	250	250	229	229	213	213
7586-4	149	149	246	246	225	225	211	211
7586-5	149	149	250	250	225	225	209	209
7586-6	149	149	246	246	225	225		
7586-7	149	149	246	246	225	225	215	215
7586-8	149	149	246	246	225	225	211	211
7586-9	149	149	246	246	225	225	211	211
7588-10	151	151	244	244	225	225	211	211
7588-11	147	147	246	246	225	225	211	211
7588-12	147	147	246	246	225	225	209	209
7588-14	149	149	244	244	225	225	211	211
7588-2	151	151	244	244	225	225	211	211
7588-3	147	147	248	248	225	225	211	211
7588-4	147	147	244	244	225	225	213	213
7588-5	147	147	244	244	225	225	213	213
7588-6	147	147	246	246	225	225	213	213
7588-7	149	149	246	246	225	225	211	211
7588-8	151	151	244	244	225	225	211	211
7588-9	151	151	244	244	225	225	211	211
7594-1	149	149	246	246	225	225	205	205
7594-10	147	147	246	246	225	225	211	211
7594-11	149	149	248	248	225	225	211	211
7594-12	149	149	244	244	225	225	205	205
7594-2	149	149	246	246	227	227	215	215
7594-3	149	149	244	244	225	225	205	205
7594-4	147	147	248	248	225	225	209	209
7594-5	151	151	246	246	225	225	213	213
7594-6	151	151	244	244	225	225	213	213
7594-7	151	151	244	244	225	225	213	213
7594-8	149	149	244	244	225	225	205	205
7594-9	149	149	246	246	225	225	213	213
7595-1	149	149	248	248	229	229	213	213
7595-10	149	149	246	246	229	229	213	213
7595-11	149	149	246	246	225	225	211	211
7595-12	149	149	246	246	225	225	211	211
7595-13	149	149			229	229	213	213
7595-3	149	149	246	246	229	229	213	213
7595-4	149	149	246	246	229	229	213	213
7595-5	151	151	250	250	229	229	215	215
7595-6	149	149	246	246	229	229	213	213
7595-7	143	143	250	250	225	225	217	217
7595-8	149	149	250	250	225	225	211	211
7595-9	149	149	248	248	225	225	213	213
7596-1	147	147	248	248	225	225	211	211
7596-10	147	147	246	246	225	225	215	215
7596-11	149	149	248	248	225	225	209	209
7596-12	149	149	246	246	227	227	213	213
7596-2	149	149	248	248	225	225	213	213
7596-3	149	149	248	248	225	225	211	211
7596-4	149	149	246	246	225	225	215	215
7596-5	149	149	244	244	225	225	213	213
7596-7	149	149	248	248	225	225	213	213
7596-8	149	149	248	248	225	225	211	211

7596-9					225	225	213	213
7599-1	149	149	250	250	225	225	213	213
7599-10	149	149	248	248	221	221	211	211
7599-11	147	147	248	248	225	225	213	213
7599-12	149	149	248	248	225	225	215	215
7599-13	149	149	248	248	225	225	213	213
7599-3	149	149	248	248	225	225	213	213
7599-4	143	143	248	248	225	225	215	215
7599-5	149	149	248	248	225	225	211	211
7599-6	147	147	246	246	221	221	211	211
7599-7	147	147	250	250	225	225	215	215
7599-8	147	147	246	246	221	221	215	215
7599-9	147	147	248	248	221	221	213	213
7600-1	149	149	248	248	225	225	211	211
7600-10	149	149	246	246	225	225	211	211
7600-11	149	149	248	248	233	233	213	213
7600-2	149	149	250	250	225	225	213	213
7600-3	149	149	244	244	225	225	217	217
7600-4	149	149	248	248	225	225	215	215
7600-5	149	149	244	244	225	225	215	215
7600-6	149	149	244	244	225	225	215	215
7600-7	149	149	244	244	225	225	215	215
7600-8	149	149	246	246	225	225	211	211
7600-9	149	149	246	246	225	225	211	211
7602-10	149	149	248	248	233	233	213	213
7602-11	149	149	244	244	233	233	209	209
7602-12	149	149	244	244	233	233	209	209
7602-2	149	149	248	248	233	233	213	213
7602-4	151	151	246	246	233	233	213	213
7602-5	147	147	246	246	233	233	213	213
7602-6	149	149	246	246	233	233	213	213
7602-7	149	149	246	246	233	233	213	213
7602-9	149	149	244	244	233	233	209	209
7604-1	149	149	250	250	225	225	213	213
7604-10	149	149	248	248	233	233	213	213
7604-11	149	149	248	248	225	225	213	213
7604-12	149	149	248	248	233	233	209	209
7604-13	149	149	248	248	225	225	213	213
7604-2	149	149	248	248	233	233	211	211
7604-3	149	149	248	248	225	225	209	209
7604-4	149	149	248	248	233	233	209	209
7604-6	149	149	248	248	233	233	211	211
7604-7	149	149	250	250	225	233	215	215
7604-8	149	149	248	248	233	233	213	213
7604-9	149	149	248	248	225	225	213	213
7606-1	147	147	244	244	225	225	215	215
7606-10	149	149	244	244	233	233	215	215
7606-11	149	149	246	246	225	225	213	213
7606-12	149	149	244	244	225	225	215	215
7606-3	147	147	244	244	225	225	213	213
7606-4	147	147	244	244	227	227	211	211
7606-5	147	147	244	244	227	227	215	215
7606-6	147	147	244	244	227	227	215	215
7606-7	147	147	248	248	225	225	213	213
7606-8	149	149	246	246	233	233	213	213
7606-9	149	149	244	244	225	225	215	215
7610-1	149	149	250	250	227	227	213	213
7610-10	149	149	248	248	225	225	213	213
7610-11	149	149	246	246	225	225	209	209
7610-12	149	149	248	248	225	225		
7610-2	149	149	248	248	225	225	215	215
7610-3	149	149	246	246	225	225	209	209
7610-4	149	149	248	248	225	225	213	213
7610-5	149	149	248	248	225	225	213	213

7610-6	149	149	246	246	225	225	213	213
7610-7	149	149	246	246	225	225	213	213
7610-8	149	149	246	246	225	225	213	213
7610-9	149	149	248	248	227	227	205	205
7612-1	149	149	248	248	233	233	213	213
7612-10	149	149	248	248	233	233	213	213
7612-11	151	151	248	248	233	233	213	213
7612-12	149	149	246	246	233	233	213	213
7612-13	149	149	248	248	233	233	213	213
7612-2	149	149	246	246	225	225	207	207
7612-3	149	149	248	248	233	233	213	213
7612-4	149	149	246	246	233	233	213	213
7612-5	149	149	248	248	233	233	215	215
7612-6	149	149	248	248	233	233	213	213
7612-8	151	151	246	246	233	233	207	207
7612-9	149	149	248	248	233	233	213	213
7616-1	149	149	244	244	233	233	213	213
7616-10	149	149	246	246	233	233	213	213
7616-13	149	149	246	246	233	233	213	213
7616-14	147	147	246	246	233	233	211	211
7616-1ii	149	149	248	248	225	225	211	211
7616-2	149	149	246	246	233	233	213	213
7616-3	149	149	246	246	233	233	213	213
7616-4	149	149	244	244	233	233	213	213
7616-5	149	149	246	246	233	233	213	213
7616-6	149	149	246	246	233	233	213	213
7616-7	149	149	246	246	233	233	213	213
7616-8	149	149	246	246	225	225	213	213
7616-9i	149	149	244	244	233	233	215	215
7625-1	149	149	244	244	225	225	211	211
7625-10	149	149	244	244	225	225	211	211
7625-11	149	149	244	244	231	231	211	211
7625-2	149	149	246	246	225	225	211	211
7625-4	149	149	244	244	225	225	209	209
7625-5	149	149	244	244	225	225	209	209
7625-7	149	149	244	244	225	225	209	209
7625-8	149	149	244	244	225	225	211	211
7628-1	147	147	246	246	225	225	211	211
7628-10	147	147	244	244	225	225	209	209
7628-11	147	147	244	244	225	225	209	209
7628-12	149	149	244	244	227	227	211	211
7628-2	149	149	244	244	229	229	209	209
7628-3	149	149	244	244	229	229	209	209
7628-4	149	149	244	244	229	229	211	211
7628-5	147	147	244	244	225	225	211	211
7628-6	149	149	244	244	227	227	211	211
7628-7	149	149	244	244	225	225	211	211
7628-8	147	147	244	244	225	225	211	211
7628-9	147	147	244	244	225	225	209	209
7640-1	149	149	246	246	225	225	213	213
7640-11	147	147	246	246	225	225	213	213
7640-12	149	149	246	246	225	225	209	209
7640-2	149	149	244	244	225	225		
7640-3	149	149	246	246	225	225	209	209
7640-4	149	149	248	248	225	225	209	209
7640-5	149	149	246	246	225	225		
7640-6	147	147	244	244	225	225	213	213
7640-7	147	147	246	246	225	225	209	209
7640-8	149	149	246	246	225	225	209	209
7640-9	149	149	246	246	225	225	213	213

Individual	GBM1413	GBM1413	GBM1060	GBM1060	GBM1021	GBM1021	GBM1075	GBM1075
1-2	158	158	209	209	256	256	300	300
1-3	158	158	209	209	245	245	300	300
1-4	158	158	209	209	254	254	300	300
1-6	158	158	209	209	245	245	300	300
1-7	158	158	209	209	258	258	300	300
1-8	158	158	209	209	264	264	300	300
1-9	148	148	209	209	247	247	300	300
1-10	148	148	209	209	258	258	300	300
1-11	158	158	206	206	254	254	300	300
1-12	158	158	209	209	252	252	300	300
1-13	158	158	209	209	252	252	300	300
4-1	164	164	209	209	252	252	300	300
4-3	164	164	209	209	250	250	300	300
4-4	153	153	209	209	268	268	300	300
4-5	153	153	209	209	266	266	300	300
4-6	164	164	209	209	252	252	300	300
4-7	164	164	209	209	250	250	300	300
4-8	153	153	209	209	268	268	300	300
4-9	164	164	209	209	266	266	300	300
4-10	164	164	209	209	266	266	300	300
4-11	164	164	209	209	266	266	300	300
4-12	164	164	209	209	266	266	300	300
4-13	164	164	209	209	266	266	300	300
7-1	164	164	209	209	266	266	300	300
7-2	164	164	209	209	268	268	300	300
7-3	164	164	209	209	268	268	300	300
7-4	164	164	209	209	268	268	300	300
7-5	164	164	209	209	264	264	300	300
7-6	164	164	209	209	266	266	300	300
7-8	164	164	209	209	268	268	300	300
7-9	164	164	209	209	266	266	300	300
7-10	164	164	209	209	268	268	300	300
7-11	164	164	209	209	268	268	300	300
7-12	164	164	209	209	266	266	300	300
9-1	158	158	206	206	252	252	300	300
9-2	164	164	209	209	264	264	300	300
9-3	158	158	209	209	254	254	300	300
9-4	153	153	209	209	254	254	300	300
9-5	158	158	209	209	254	254	300	300
9-6	164	164	209	209	252	252	300	300
9-7	158	158	209	209	254	254	296	296
9-8	164	164	209	209	258	258	296	296
9-9	164	164	209	209	252	252	300	300
9-10	158	158	209	209	252	252	300	300
9-11	158	158	209	209	256	256	296	296
9-12	158	158	209	209			300	300
10-1	158	158	209	209	258	258	300	300
10-2	164	164	209	209	252	252	300	300
10-3	164	164	209	209	254	254	300	300
10-4	164	164	209	209	258	258	300	300
10-5	158	158	209	209	256	256	300	300
10-6	164	164	209	209	252	252	300	300
10-7	158	158	209	209	252	252	300	300
10-8	164	164	209	209	258	258	300	300
10-9	164	164	206	206	254	254	292	292
10-10	164	164	209	209	258	258	300	300
10-11	164	164	206	206	254	254	300	300

10-12	164	164	206	206	252	252	300	300
10-13	164	164	206	206	268	268	300	300
7561-10b	164	164	206	206	268	268	300	300
7561-11b	164	164	206	206	270	270	300	300
7561-12b	164	164	206	206	268	268	300	300
7561-1a	164	164	212	212	258	258	304	304
7561-2a	164	164	212	212	258	258	302	302
7561-3a	164	164	212	212	258	258	302	302
7561-4a	164	164	212	212	258	258	302	302
7561-5a	164	164	212	212	258	258	302	302
7561-6a	164	164	212	212	258	258	302	302
7561-7b	164	164	206	206	270	270	300	300
7561-8b	164	164	206	206	268	268	300	300
7561-9b	164	164	206	206	264	264	304	304
7566-1	164	164	212	212	266	266	292	292
7566-10	164	164	206	206	252	252	300	300
7566-2	164	164	212	212	264	264	292	292
7566-3	164	164	212	212	264	264	292	292
7566-4	164	164	206	206	273	273	300	300
7566-5	164	164	206	206	273	273	300	300
7566-6	164	164	212	212	254	254	300	300
7566-8	164	164	206	206	252	252	300	300
7566a-11	164	164	209	209	254	254	302	302
7566a-12	164	164	212	212	266	266	292	292
7566a-7	164	164	206	206	252	252	300	300
7566a-9	164	164	206	206	254	254	300	300
7568-10	158	158	209	209	243	243	300	300
7568-11	158	158	209	209	262	262	300	300
7568-12	158	158	209	209	262	262	300	300
7568-13	158	158	209	209	264	264	300	300
7568-1i	158	158	209	209	262	262	300	300
7568-1ii	158	158	206	206	262	262	302	302
7568-2	158	158	206	206	260	260	302	302
7568-3	164	164	209	209	266	266	300	300
7568-4	158	158	209	209	262	262	300	300
7568-5	164	164	212	212	256	256	300	300
7568-6	158	158	206	206	256	256	300	300
7568-7	158	158	209	209	262	262	300	300
7568-8	164	164	209	209	262	262	300	300
7568-9	158	158	209	209	262	262	300	300
7569-1	164	164	212	212	254	254	302	302
7569-10	164	164	212	212	252	252	300	300
7569-11	164	164	212	212			300	300
7569-12	164	164	212	212	254	254	300	300
7569-13	164	164	212	212	252	252	300	300
7569-2	164	164	212	212			300	300
**7569-3**	**158**	**158**	**206**	**206**	**256**	**256**	**300**	**300**
7569-4	164	164	212	212	252	252	300	300
7569-5	164	164	212	212	252	252	300	300
7569-6	164	164	212	212	252	252	300	300
7569-8	164	164	212	212	252	252	300	300
7569-9	164	164	212	212	252	252	300	300
7570-1	158	158	206	206	266	266	300	300
7570-10	158	158	206	206	256	256	300	300
7570-11	158	164	206	206	254	254	300	300
7570-12	158	158	206	206	254	254	300	300
7570-13	158	158	209	209	262	262	300	300
7570-2	158	158	206	206	256	256	300	300
7570-3	158	158	206	206	254	254	300	300
7570-4	158	158	206	206	262	262	300	300
7570-5	158	158	209	209	266	266	300	300
7570-6	158	158	206	206	256	256	300	300
7570-7	158	158	206	206	262	262	300	300
7570-8	158	158	209	209	254	254	300	300

7570-9	158	158	206	206	254	254	300	300
7571-10	158	158	209	209	254	254	300	300
7571-12	158	158	209	209	254	254	300	300
7571-13	169	169	209	209	262	262	292	292
7571-1a	158	158	206	206	273	273	292	292
7571-2	158	158	209	209	254	254	300	300
7571-3	169	169	209	209	262	262	292	292
7571-4	158	158	206	206	273	273	292	292
7571-5	158	158	206	206	268	268	300	300
7571-6	158	158	209	209	254	254	292	292
7571-7	158	158	209	209	266	266	300	300
7571-9	169	169	209	209	252	252	292	292
7575-1	158	158	209	209	252	252	300	300
7575-2	158	158	209	209	252	252	300	300
7575-9	158	158	209	209	252	252	300	300
7575a-10	158	158	209	209	254	254	300	300
7575a-11	158	158	209	209	254	254	300	300
7575a-12	158	158	209	209	252	252	300	300
7575a-13	158	158	209	209	252	252	300	300
7575a-3	158	158	209	209	254	254	302	302
7575a-4	158	158	209	209	252	252	300	300
7575a-5	158	158	209	209	252	252	300	300
7575a-6	158	158	209	209	252	252	300	300
7575a-7	158	158	209	209	252	252	300	300
7577-1	164	164	206	206	260	260	292	292
7577-10	164	164	206	206	258	258	292	292
7577-11	158	158	209	209	252	252	300	300
7577-2	164	164	206	206	258	258	292	292
7577-3	164	164	206	206	258	258	292	292
7577-4	164	164	209	209	254	254	300	300
7577-5	164	164	206	206	258	258	292	292
7577-6	164	164	206	206	258	258	300	300
7577-7	164	164	206	206	258	258	292	292
7577-8	164	164	206	206	258	258	292	292
7577-9	164	164	206	206	260	260	300	300
7581-10	164	164	209	209	258	258	300	300
7581-11	158	158	209	209	254	254	302	302
7581-12	158	158	209	209	254	254	302	302
7581-2	158	158	209	209	252	252	300	300
7581-3	153	153	209	209	262	262	300	300
7581-4	153	153	206	206	258	258	292	292
7581-5	153	153	206	206	247	247	300	300
7581-6	153	153	209	209	256	256	300	300
7581-7	153	153	209	209	256	256	300	300
7581-8	153	153	206	206	252	252	300	300
7581-9	153	153	209	209	252	252	300	300
7583-1	158	158	209	209	254	254	300	300
7583-10	169	169	209	209	252	252	292	292
7583-11	169	169	209	209	252	252	292	292
7583-12	158	158	209	209	266	266	300	300
7583-2	158	158	209	209	254	254	300	300
7583-3	158	158	209	209	262	262	300	300
7583-4	169	169	209	209	252	252	292	292
7583-5	169	169	209	209	250	250	292	292
7583-6	169	169	209	209	250	250	292	292
7583-7	169	169	209	209	250	250	292	292
7583-8	169	169	209	209	250	250	292	292
7583-9	169	169	209	209	250	250	292	292
7584-1	164	164	209	209	252	252	292	292
7584-10	164	164	206	206	254	254	300	300
7584-11	158	158	209	209	260	260	300	300
7584-12	158	158	209	209	252	252	300	300
7584-2	158	158	209	209	252	252	300	300
7584-3	158	158	209	209	245	245	300	300

7584-4	158	158	209	209	264	264	300	300
7584-5	164	164	209	209	252	252	300	300
7584-6	158	158	209	209	254	254	300	300
7584-7	158	158	209	209	254	254	300	300
7584-8	164	164	209	209	252	252	300	300
7584-9	158	158	209	209	268	268	300	300
7586-1	158	158						
7586-10	153	153	206	206	258	258	300	300
7586-11	158	158	206	206	258	258	300	300
7586-12	158	158	206	206	256	256	300	300
7586-2	158	158	212	212	247	247	300	300
7586-3	158	158	209	209	252	252	300	300
7586-4	158	158	206	206	256	256	300	300
7586-5	158	158	209	209	264	264	300	300
7586-6	158	158	209	209	266	266	302	302
7586-7	158	158	209	209	266	266	302	302
7586-8	158	158	206	206	256	256	300	300
7586-9	158	158	206	206	258	258	300	300
7588-10	158	158	209	209	258	258	300	300
7588-11	158	158	212	212	266	266	300	300
7588-12	158	158	212	212	256	256	300	300
7588-14	158	158	206	206	256	256	300	300
7588-2	158	158	209	209	258	258	300	300
7588-3	158	158	212	212	252	252	300	300
7588-4	158	158	206	206	256	256	300	300
7588-5	158	158	206	206	256	256	300	300
7588-6	158	158	206	206	256	256	300	300
7588-7	158	158	206	206	256	256	300	300
7588-8	158	158	212	212	256	256	292	292
7588-9	158	158	209	209	258	258	300	300
7594-1	158	158	209	209	262	262	300	300
7594-10	148	148	206	206	252	252	292	292
7594-11	158	158	209	209	254	254	300	300
7594-12	158	158	209	209	256	256	300	300
7594-2	158	158	209	209	245	245	300	300
7594-3	158	158	209	209	254	254	300	300
7594-4	164	164	206	206	252	252	300	300
7594-5	164	164	209	209	252	252	300	300
7594-6	164	164	209	209	252	252	300	300
7594-7	164	164	209	209	252	252	300	300
7594-8	158	158	209	209	254	254	300	300
7594-9	158	158	209	209	258	258	302	302
7595-1	158	158	209	209	254	254	302	302
7595-10	158	158	209	209	256	256	300	300
7595-11	164	164	209	209	258	258	300	300
7595-12	164	164	209	209	258	258	300	300
7595-13	158	158	209	209	258	258	300	300
7595-3	158	158	209	209	252	252	300	300
7595-4	158	158	209	209	264	264	300	300
7595-5	158	158	209	209	266	266	300	300
7595-6	158	158	209	209	252	252	300	300
7595-7	164	164	209	209	264	264	300	300
7595-8	164	164	209	209	266	266	300	300
7595-9	164	164	209	209	268	268	302	302
7596-1	158	158	206	206	254	254	300	300
7596-10	158	158	209	209	256	256	300	300
7596-11	158	158	206	206	247	247	300	300
7596-12	158	158	209	209	256	256	302	302
7596-2	158	158						
7596-3	164	164	212	212	247	247	300	300
7596-4	158	158	212	212	262	262	300	300
7596-5	158	158	206	206	252	252	300	300
7596-7	158	158	206	206	262	262	300	300
7596-8	158	158	209	209	262	262	300	300

7596-9	158	158						
7599-1	164	164	209	209	254	254	302	302
7599-10	164	164	206	206	262	262	300	300
7599-11	158	158	209	209	268	268	300	300
7599-12	164	164	209	209	256	256	300	300
7599-13	164	164	209	209	256	256	300	300
7599-3	164	164	209	209	256	256	300	300
7599-4	158	158	209	209	252	252	300	300
7599-5	164	164	209	209	273	273	300	300
7599-6	158	158	209	209	258	258	300	300
7599-7	158	158	209	209	268	268	302	302
7599-8	164	164	206	206	266	266	300	300
7599-9	164	164	206	206	266	266	300	300
7600-1	164	164	206	206	266	266	300	300
7600-10	158	158	206	206	262	262	300	300
7600-11	164	164	212	212	256	256	300	300
7600-2	164	164	206	206	252	252	300	300
7600-3	158	158	206	206	252	252	292	292
7600-4	164	164	206	206	252	252	300	300
7600-5	158	158	212	212	247	247	302	302
7600-6	158	158	212	212	247	247	302	302
7600-7	158	158	212	212	247	247	302	302
7600-8	158	158	206	206	262	262	300	300
7600-9	158	158	206	206	262	262	300	300
7602-10	158	158	209	209	268	268	300	300
7602-11	164	164	212	212	252	252	300	300
7602-12	164	164	212	212	254	254	300	300
7602-2	169	169	206	206	254	254	302	302
7602-4	164	164	212	212	252	252	292	292
7602-5	169	169	212	212	268	268	300	300
7602-6	164	164	212	212	256	256	300	300
7602-7	169	169	206	206	252	252	300	300
7602-9	164	164	212	212	268	268	300	300
7604-1	164	164	212	212	258	258	304	304
7604-10	164	164	212	212	252	252	304	304
7604-11	164	164	212	212	256	256	300	300
7604-12	164	164	212	212	258	258	300	300
7604-13	164	164	212	212	256	256	300	300
7604-2	164	164	212	212	256	256	300	300
7604-3	164	164	212	212	258	258	302	302
7604-4	164	164	212	212	256	256	300	300
7604-6	164	164	212	212	256	256	300	300
7604-7	164	169	212	212	264	264	304	304
7604-8	164	164	212	212	252	252	292	292
7604-9	164	164	212	212	258	258	302	302
7606-1	158	158	209	209	252	252	300	300
7606-10	164	164	212	212	252	252	300	300
7606-11	158	158	209	209	245	245	300	300
7606-12	158	158	209	209	245	245	300	300
7606-3	158	158	199	199	245	245	300	300
7606-4	158	158	209	209	247	247	300	300
7606-5	158	158	209	209	252	252	300	300
7606-6	158	158	209	209	252	252	300	300
7606-7	164	164	212	212	256	256	300	300
7606-8	164	164	212	212	256	256	300	300
7606-9	158	158	209	209	245	245	300	300
7610-1	158	158	206	206	247	247	300	300
7610-10	164	164	209	209	262	262	300	300
7610-11	164	164	209	209	264	264	300	300
7610-12	164	164	209	209	262	262	300	300
7610-2	148	148	209	209	247	247	300	300
7610-3	158	158	206	206	262	262	300	300
7610-4	158	158	209	209	262	262	300	300
7610-5	158	158	209	209	256	256	300	300

7610-6	158	158	209	209	256	256	300	300
7610-7	158	158	209	209	256	256	300	300
7610-8	158	158	209	209	256	256	300	300
7610-9	158	158	209	209	254	254	300	300
7612-1	164	164	212	212	254	254	300	300
7612-10	164	164	212	212	256	256	300	300
7612-11	164	164	212	212	254	254	302	302
7612-12	164	164	212	212	256	256	300	300
7612-13	164	164	212	212	254	254	300	300
7612-2	169	169	206	206	252	252	302	302
7612-3	164	164	212	212	256	256	300	300
7612-4	164	164	209	209	256	256	302	302
7612-5	169	169	212	212	268	268	300	300
7612-6	169	169	212	212	268	268	300	300
7612-8	158	158	206	206	252	252	300	300
7612-9	164	164	212	212	256	256	300	300
7616-1	158	158	212	212	254	254	300	300
7616-10	158	158	212	212	252	252	300	300
7616-13	158	158	212	212	252	252	300	300
7616-14	164	164	212	212	254	254	300	300
7616-1ii	164	164	209	209	266	266	300	300
7616-2	158	158	212	212	254	254	300	300
7616-3	158	158	209	209	258	258	292	292
7616-4	158	158	212	212	254	254	300	300
7616-5	158	158	212	212	254	254	300	300
7616-6	158	158	212	212	254	254	300	300
7616-7	164	164	212	212	254	254	302	302
7616-8	158	158	209	209	254	254	292	292
7616-9i	158	158	212	212	254	254	300	300
7625-1	164	164	209	209	247	247	300	300
7625-10	164	164	209	209	268	268	300	300
7625-11	164	164	209	209	268	268	300	300
7625-2	164	164	209	209	268	268	302	302
7625-4	164	164	209	209	268	268	300	300
7625-5	164	164	209	209	268	268	302	302
7625-7	164	164	209	209	268	268	302	302
7625-8	164	164	209	209	268	268	300	300
7628-1	153	153	209	209	268	268	300	300
7628-10	153	153	209	209	268	268	300	300
7628-11	153	153	209	209	268	268	300	300
7628-12	164	164	209	209	266	266	300	300
7628-2	164	164	209	209	266	266	300	300
7628-3	164	164	209	209	266	266	300	300
7628-4	164	164	209	209	266	266	300	300
7628-5	153	153	209	209	268	268	300	300
7628-6	164	164	209	209	252	252	300	300
7628-7	148	148	209	209	264	264	300	300
7628-8	153	153	209	209	268	268	300	300
7628-9	153	153	209	209	268	268	300	300
7640-1	158	158	206	206	260	260	302	302
7640-11	158	158	199	199	252	252	300	300
7640-12	158	158	206	206	252	252	300	300
7640-2	158	158	209	209	273	273	300	300
7640-3	158	158	209	209	252	252	300	300
7640-4	158	158	206	206	258	258	300	300
7640-5			209	209	258	258	302	302
7640-6	158	158	209	209	258	258	300	300
7640-7	158	158	206	206	254	254	300	300
7640-8	158	158	206	206	273	273	300	300
7640-9	158	158	206	209	273	273	300	300

Individual	GBM1516	GBM1516	GBM1405	GBM1405	GBM1063	GBM1063	GBM1280	GBM1280
1-2	95	95	290	290	204	204	277	277
1-3	95	95	290	290	216	216	277	277
1-4	95	95	284	284	220	220	277	277
1-6	95	95	290	290	216	216	277	277
1-7	95	95	276	276	220	220	277	277
1-8	95	95	290	290	224	224	277	277
1-9	95	95	284	284	212	212	286	286
1-10	95	95	290	290	216	216	277	277
1-11	95	95	288	288	200	200	286	286
1-12	97	97	286	286	220	220	277	277
1-13	95	95	286	286	212	212	277	277
4-1	95	95	276	276	212	212	277	277
4-3	95	95	276	276	212	212	277	277
4-4	95	95	288	288	212	212	277	277
4-5	95	95	288	288	212	212	277	277
4-6	95	95	280	280	212	212	277	277
4-7	95	95	286	286	212	212	277	277
4-8	95	95	288	288	212	212	277	277
4-9	95	95	288	288	212	212	277	277
4-10	95	95	276	276	212	212	277	277
4-11	95	95	284	284	212	212	277	277
4-12	97	97	276	276	212	212	277	277
4-13	95	95	284	284	212	212	277	277
7-1	95	95	288	288	212	212	277	277
7-2	93	93	284	284	212	212	277	277
7-3	93	93	286	286	212	212	277	277
7-4	93	93	286	286	212	212	277	277
7-5	93	93	286	286	212	212	280	280
7-6	93	93	286	286	212	212	280	280
7-8	93	93	286	286	212	212	277	277
7-9	93	93	288	288	212	212	277	277
7-10	93	93	288	288	212	212	277	277
7-11	93	93	284	284	212	212	277	277
7-12	93	93	284	284	212	212	277	277
9-1	93	93	286	286	208	208	283	283
9-2	93	93	286	286	208	208	289	289
9-3	93	93	288	288	208	208	280	280
9-4	93	93	284	284	204	204	289	289
9-5	93	93	284	284	208	208	280	280
9-6	93	93	284	284	208	208	289	289
9-7	93	93	284	284	208	208	283	283
9-8	93	93	284	284	200	200	277	277
9-9	93	93	286	286	204	204	280	280
9-10	93	93	288	288	208	208	289	289
9-11	93	93	288	288	208	208	283	283
9-12	93	93	284	284	208	208	280	280
10-1	93	93	284	284	196	196	283	283
10-2	95	95	288	288	208	208	289	289
10-3	95	95	288	288	220	220	277	277
10-4	95	95	288	288	208	208	283	283
10-5	95	95	290	290	208	208	283	283
10-6	95	95	288	288	200	200	283	283
10-7	95	95	290	290	208	208	283	283
10-8	95	95	288	288	208	208	289	289
10-9	95	95	288	288	204	204	283	283
10-10	95	95	290	290	208	208	289	289
10-11	95	95	288	288	204	204	283	283

10-12	95	95	286	286	212	212	289	289
10-13	95	95	286	286	208	208	289	289
7561-10b	95	95	288	288	220	220	283	283
7561-11b	99	99	288	288	220	220	283	283
7561-12b	95	95	288	288	220	220	283	283
7561-1a	95	95	288	288	212	212	283	283
7561-2a	95	95	290	290	212	212	283	283
7561-3a	95	95	288	288	212	212	283	283
7561-4a	95	95	288	288	212	212	283	283
7561-5a	99	99	290	290	212	212	283	283
7561-6a	95	95	290	290	212	212	283	283
7561-7b	95	95	288	288	204	204	283	283
7561-8b	99	99	292	292	220	220	283	283
7561-9b	99	99	288	288	204	220	283	283
7566-1	97	97	296	296	204	204	283	283
7566-10	97	97	296	296	212	212	289	289
7566-2	97	97	296	296	220	220	283	283
7566-3	103	103	296	296	220	220	283	283
7566-4	97	97	296	296	212	212	277	277
7566-5	97	97	284	284	212	212	277	277
7566-6	97	97	280	280	212	212	277	277
7566-8	93	93	284	284	212	212	289	289
7566a-11	97	97	296	296	212	212	283	283
7566a-12	95	95	288	288	220	220	283	283
7566a-7	97	97	288	288	212	212	289	289
7566a-9	97	97	296	296	212	212	289	289
7568-10	97	97	296	296	212	212	289	289
7568-11	97	97	296	296	212	212	289	289
7568-12	99	99	290	290	212	212	289	289
7568-13	99	99	288	288	204	204	277	277
7568-1i	99	99	290	290	212	212	289	289
7568-1ii	99	99	290	290	204	204	277	277
7568-2	99	99	288	288	204	204	277	277
7568-3	99	99	284	284	212	212	277	277
7568-4	97	97	284	284	212	212	289	289
7568-5	99	99	288	288	212	212	283	283
7568-6	99	99	290	290	212	212	289	289
7568-7	99	99	288	288	212	212	289	289
7568-8	99	99	290	290	212	212	289	289
7568-9	99	99	288	288	212	212	289	289
7569-1	95	95	284	284	208	208	277	277
7569-10	97	97	284	284	208	208	277	277
7569-11	99	99	290	290	208	208	277	277
7569-12	97	97	284	284	208	208	277	277
7569-13	97	97	284	284	208	208	277	277
7569-2	103	103	284	284	208	208	277	277
**7569-3**	97	97	284	284	**208**	**208**	**277**	**277**
7569-4	97	97	290	290	208	208	277	277
7569-5	95	95	284	284	208	208	277	277
7569-6	97	97	284	284	208	208	277	277
7569-8	97	97	290	290	208	208	277	277
7569-9	97	97	284	284	208	208	277	277
7570-1	97	97	284	284	208	208	277	277
7570-10	95	95	284	284	208	208	289	289
7570-11	95	95	284	284	208	212	289	289
7570-12	95	95	276	276	208	208	289	289
7570-13	95	95	276	276	212	212	277	277
7570-2	95	95	288	288	196	196	289	289
7570-3	95	95	276	276	208	208	289	289
7570-4	95	95	276	276	208	208	277	277
7570-5	97	97	288	288	208	208	289	289
7570-6	95	95	284	284	208	208	289	289
7570-7	97	97	288	288	208	208	277	277
7570-8	95	95	276	276	208	208	289	289

7570-9	95	95	276	276	208	208	289	289
7571-10	95	95	276	276	208	208	277	277
7571-12	95	95	276	276	196	208	289	289
7571-13	95	95	276	276	227	227	289	289
7571-1a	95	95	276	276	200	200	289	289
7571-2	95	95	276	276	208	208	289	289
7571-3	95	95	276	276	227	227	289	289
7571-4	95	95	276	276	200	200	289	289
7571-5	95	95	288	288	204	204	277	277
7571-6	95	95	276	276	208	208	289	289
7571-7	95	95	276	276	208	208	289	289
7571-9	95	95	276	276	227	227	289	289
7575-1	95	95	284	284	212	212	283	283
7575-2	95	95	284	284	212	212	283	283
7575-9	95	95	276	276	212	212	283	283
7575a-10	95	95	284	284	212	212	283	283
7575a-11	95	95	284	284	212	212	283	283
7575a-12	95	95	284	284	212	212	283	283
7575a-13	95	95	284	284	212	212	283	283
7575a-3	95	95	288	288	212	212	283	283
7575a-4	95	95	284	284	212	212	283	283
7575a-5	95	95	284	284	212	212	283	283
7575a-6	95	95	284	284	212	212	283	283
7575a-7	99	99	276	276	212	212	283	283
7577-1	99	99	288	288	200	200	280	280
7577-10	99	99	288	288	200	200	280	280
7577-11	95	95	276	276	220	220	289	289
7577-2	95	95	276	276	200	200	280	280
7577-3	97	97	288	288	200	200	280	280
7577-4	95	95	288	288	204	204	289	289
7577-5	95	95	292	292	200	200	280	280
7577-6	97	97	288	288	216	216	289	289
7577-7	95	95	288	288	200	200	280	280
7577-8	95	95	284	284	200	200	280	280
7577-9	95	95	288	288	204	204	280	280
7581-10	95	95	290	290	200	200	289	289
7581-11	101	101	288	288	208	208	289	289
7581-12	95	95	284	284	208	208	289	289
7581-2	95	95	288	288	200	200	289	289
7581-3	95	95	296	296	200	200	283	283
7581-4	101	101	288	288	196	196	289	289
7581-5	101	101	288	288	196	196	289	289
7581-6	101	101	288	288	208	208	289	289
7581-7	101	101	290	290	216	216	283	283
7581-8	95	95	288	288	196	196	289	289
7581-9	101	101	288	288	208	208	289	289
7583-1	95	95	284	284	200	200	289	289
7583-10	95	95	280	280	208	208	286	286
7583-11	103	103	288	288	208	208	286	286
7583-12	93	93	290	290	208	208	289	289
7583-2	95	95	288	288	200	200	289	289
7583-3	95	95	292	292	216	216	289	289
7583-4	95	95	290	290	208	208	286	286
7583-5	95	95	288	288	208	208	286	286
7583-6	93	93	290	290	208	208	286	286
7583-7	95	95	276	276	208	208	286	286
7583-8	95	95	288	288	208	208	286	286
7583-9	95	95	292	292	208	208	286	286
7584-1	95	95	284	284	208	208	283	283
7584-10	95	95	284	284	208	208	289	289
7584-11	95	95	284	284	196	196	277	277
7584-12	95	95	284	284	196	196	277	277
7584-2	95	95	284	284	204	204	277	277
7584-3	101	101	284	284	204	204		

7584-4	95	95	284	284	200	200	280	280
7584-5	95	95	296	296	200	200	283	283
7584-6	95	95	284	284	196	196	277	277
7584-7	95	95	288	288	208	208	283	283
7584-8	95	95	284	284	200	200	283	283
7584-9	95	95	284	284	204	204	277	277
7586-1	99	99	276	276	204	204	280	280
7586-10	99	99	284	284	204	204	289	289
7586-11	99	99	284	284	204	204	280	280
7586-12	97	97	284	284	204	204	280	280
7586-2	99	99	276	276	200	200	280	280
7586-3	99	99	284	284	200	200	289	289
7586-4	97	97	288	288	204	204	280	280
7586-5	97	97	288	288	200	200	289	289
7586-6	97	97	288	288	200	200	289	289
7586-7	97	97	284	284	200	200	289	289
7586-8	99	99	292	292	204	204	280	280
7586-9	99	99	276	276	204	204	280	280
7588-10	99	99	288	288	208	208	289	289
7588-11	95	95	284	284	208	208	289	289
7588-12	95	95	296	296	208	208	289	289
7588-14	97	97	286	286	200	200	289	289
7588-2	95	95	296	296	208	208	289	289
7588-3	97	97	284	284	204	204	289	289
7588-4	97	97	286	286	208	208	286	286
7588-5	95	95	284	284	208	208	286	286
7588-6	95	95	284	284	208	208	286	286
7588-7	95	95	286	286	200	200	289	289
7588-8	97	97	286	286	216	216	286	286
7588-9	95	95	296	296	208	208	289	289
7594-1	95	95	292	292	216	216	277	277
7594-10	95	95	292	292	200	200	277	277
7594-11	95	95	276	276	208	208	277	277
7594-12	95	95	276	276	216	216	289	289
7594-2	95	95	292	292	204	204	277	277
7594-3	95	95	290	290	216	216	289	289
7594-4	95	95	292	292	204	204	289	289
7594-5	93	93	296	296	212	212	277	277
7594-6	95	95	292	292	212	212	277	277
7594-7	95	95	292	292	212	212	277	277
7594-8	93	93	276	276	216	216	289	289
7594-9	95	95	280	280	204	208	277	277
7595-1	99	99	296	296	212	212	277	277
7595-10	97	97	288	288	212	212	277	277
7595-11	99	99	288	288	216	216	289	289
7595-12	95	95	288	288	216	216	289	289
7595-13	97	97	290	290	212	212	277	277
7595-3	95	95	284	284	212	212	277	277
7595-4	99	99	296	296	212	212	277	277
7595-5	99	99	284	284	212	212	277	277
7595-6	97	97	290	290	212	212	277	277
7595-7	95	95	286	286	208	208	280	280
7595-8	99	99	284	284	212	212	277	277
7595-9	95	95	288	288	208	208	277	277
7596-1	97	97	284	284	208	208	289	289
7596-10	95	95	284	284	212	212	277	277
7596-11	95	95	284	284	208	208	289	289
7596-12	97	97	284	284	212	212	280	280
7596-2	95	95	284	284	212	212	283	283
7596-3	95	95	284	284	212	212	277	277
7596-4	95	95	284	284	208	208	277	277
7596-5	97	97	284	284	216	216	280	280
7596-7	95	95	286	286	200	200	277	277
7596-8	97	97	284	284	212	212	280	280

7596-9	95	95	284	284	200	200	289	289
7599-1	95	95	276	276	200	200	277	277
7599-10	97	97	296	296	200	200	277	277
7599-11	95	95	290	290	200	200	277	277
7599-12	95	95	296	296	200	200	277	277
7599-13	97	97	290	290	208	208	277	277
7599-3	95	95	296	296	200	200	277	277
7599-4	97	97	290	290	200	200	277	277
7599-5	97	97	290	290	200	200	277	277
7599-6	97	97	290	290	200	200	277	277
7599-7	97	97	296	296	200	200	277	277
7599-8	97	97	296	296	200	200	277	277
7599-9	95	95	276	276	200	200	277	277
7600-1	95	95	288	288	208	208	277	277
7600-10	95	95	288	288	208	208	283	283
7600-11	95	95	288	288	212	212	283	283
7600-2	95	95	288	288	204	204	277	277
7600-3	95	95	288	288	208	208	289	289
7600-4	95	95	288	288	204	204	277	277
7600-5	95	95	276	276	208	208	289	289
7600-6	95	95	276	276	208	208	289	289
7600-7	95	95	290	290	208	208	289	289
7600-8	95	95	288	288	208	208	283	283
7600-9	95	95	290	290	208	208	283	283
7602-10	95	95	276	276	212	212	283	283
7602-11	95	95	288	288	208	208	277	277
7602-12	95	95	288	288	208	208	277	277
7602-2	95	95	288	288	212	212	283	283
7602-4	95	95	290	290	220	220	289	289
7602-5	95	95	288	288	212	212	283	283
7602-6	95	95	286	286	212	212	283	283
7602-7	95	95	290	290	212	212	283	283
7602-9	95	95	290	290	220	220	277	277
7604-1	95	95	292	292	208	208	289	289
7604-10	95	95	290	290	220	220	289	289
7604-11	93	93	288	288	212	212	283	283
7604-12	93	93	290	290	212	212	283	283
7604-13	95	95	280	280	212	212	283	283
7604-2	103	103	286	286	212	212	283	283
7604-3	95	95	286	286	212	212	283	283
7604-4	93	93	288	288	212	212	283	283
7604-6	95	95	290	290	212	212	283	283
7604-7	95	95	288	288	220	220	289	289
7604-8	93	93	290	290	220	220	289	289
7604-9	95	95	284	284	212	212	283	283
7606-1	97	97	292	292	204	204	277	277
7606-10	95	95	288	288	208	208	289	289
7606-11	97	97	290	290	204	204	277	277
7606-12	97	101	280	280	204	204	277	277
7606-3	95	95	292	292	208	208	277	277
7606-4	95	95	292	292	212	212	277	277
7606-5	97	97	280	280	212	212	277	277
7606-6	97	97	290	290	212	212	277	277
7606-7	97	97	290	290	212	212	283	283
7606-8	97	97	290	290	212	212	283	283
7606-9	95	99	284	284	204	204	277	277
7610-1	95	95	288	288	200	200	277	277
7610-10	95	95	288	288	200	200	277	277
7610-11	95	95	288	288	208	208	277	277
7610-12	95	95	288	288	200	200	277	277
7610-2	95	95	288	288	208	208	277	277
7610-3	95	95	276	276	200	200	277	277
7610-4	95	95	288	288	200	200	277	277
7610-5	95	95	280	280	208	208	277	277

7610-6	95	95	276	276	208	208	277	277
7610-7	95	95	288	288	208	208	277	277
7610-8	95	95	290	290	208	208	277	277
7610-9	95	95	288	288	200	200	277	277
7612-1	95	95	288	288	204	204	283	283
7612-10	95	95	288	288	212	212	283	283
7612-11	95	95	288	288	220	220	289	289
7612-12	95	95	280	280	216	216	283	283
7612-13	95	95	276	276	204	204	283	283
7612-2	95	95	288	288	220	220	283	283
7612-3	99	99	288	288	204	204	283	283
7612-4	95	95	290	290	220	220	289	289
7612-5	95	95	288	288	220	220	277	277
7612-6	95	95	288	288	212	212	283	283
7612-8	95	95	290	290	212	212	277	277
7612-9	95	95	280	280	212	212	283	283
7616-1	95	95	288	288	208	208	289	289
7616-10	93	93	288	288	208	208	289	289
7616-13	93	93	286	286	208	208	289	289
7616-14	93	93	286	286	220	220	280	280
7616-1ii	93	93	286	286	208	208	277	277
7616-2	93	93	288	288	208	208	289	289
7616-3	93	93	288	288	208	208	289	289
7616-4	93	93	288	288	208	208	289	289
7616-5	93	93	286	286	208	208	289	289
7616-6	93	93	290	290	208	208	289	289
7616-7	93	93			212	212	283	283
7616-8	93	93	286	286	220	220	289	289
7616-9i	93	93	286	286	208	208	289	289
7625-1	93	93	284	284	212	212	277	277
7625-10	93	93	286	286	212	212	277	277
7625-11	93	93	286	286	212	212	277	277
7625-2	93	93	284	284	212	212	277	277
7625-4	93	93	284	284	212	212	277	277
7625-5	97	97	284	284	212	212	277	277
7625-7	93	93	284	284	212	212	277	277
7625-8	93	93	284	284	212	212	277	277
7628-1	99	99	284	284	212	212	277	277
7628-10	93	93	276	276	212	212	277	277
7628-11	97	97	276	276	212	212	277	277
7628-12	97	97	284	284	222	222	277	277
7628-2	97	97	276	276	212	212	277	277
7628-3	99	99	290	290	212	212	277	277
7628-4	103	103	284	284	212	212	277	277
7628-5	99	99	284	284	212	212	277	277
7628-6	97	97	284	284	212	212	277	277
7628-7	97	97	284	284	196	196	277	277
7628-8	103	103	284	284	212	212	277	277
7628-9	95	95	280	280	212	212	277	277
7640-1	95	95	276	276	216	216	289	289
7640-11	97	97	284	284	208	208	277	277
7640-12	95	95	276	276	204	204	289	289
7640-2	95	95	276	276	204	204	289	289
7640-3	95	95	276	276	200	200	277	277
7640-4	95	95	284	284	200	200	280	280
7640-5	95	95	276	276	216	216	289	289
7640-6	95	95	276	276	216	216	289	289
7640-7	97	97	284	284	216	216	289	289
7640-8	95	95	280	280	204	204	289	289
7640-9	95	95	276	276	204	204	289	289

Individual	GBM1323	GBM1323	GBM1464	GBM1464	GBM1002	GBM1002	GBM1026	GBM1026
1-2	122	122	172	172	269	269	212	212
1-3	114	114	172	172	274	274	212	212
1-4	122	122	172	172	280	280	212	212
1-6	114	114	172	172	277	277	212	212
1-7	122	122	168	168	280	280	212	212
1-8	122	122	168	168	274	274	214	214
1-9	122	122	168	168	269	269	212	212
1-10	122	122	172	172	277	277	212	212
1-11	122	122	172	172	280	280	212	212
1-12	122	122	170	170	269	269	212	212
1-13	122	122	168	168	269	269	212	212
4-1	122	122	170	170	277	277	212	212
4-3	122	122	168	168	274	274	212	212
4-4	122	122	160	160	274	274	210	210
4-5	122	122	160	160	274	274	212	212
4-6	122	122	170	170	277	277	212	212
4-7	122	122	168	168	274	274	212	212
4-8	122	122	160	160	277	277	210	210
4-9	122	122	160	160	274	274	212	212
4-10	122	122	160	160	280	280	212	212
4-11	122	122	160	160	274	274	210	210
4-12	122	122	160	160	274	274	212	212
4-13	122	122	160	160	274	274	212	212
7-1	114	114	164	164	344	344	212	212
7-2	114	114	164	164	344	344	212	212
7-3	114	114	164	164	344	344	210	210
7-4	114	114	164	164	344	344	210	210
7-5	122	122	172	172	277	277	210	210
7-6	122	122	172	172	274	274	210	210
7-8	122	122	164	164	277	277	210	210
7-9	114	114	164	164	274	274	210	210
7-10	122	122	164	164	274	274	212	212
7-11	122	122	164	164	274	274	212	212
7-12	122	122	164	164	274	274	210	210
9-1	118	118	160	160	277	277	210	210
9-2	122	122	170	170	269	269	212	212
9-3	122	122	160	160	277	277	210	210
9-4	122	122	170	170	277	277	210	210
9-5	122	122	164	164	277	277	210	210
9-6	128	128	124	124	277	277	212	212
9-7	112	112	160	160	277	277	212	212
9-8	122	122	160	160	274	274	212	212
9-9	118	118	168	168			210	210
9-10	128	128	160	160	277	277	210	210
9-11	112	112	170	170	277	277	210	210
9-12	122	122	170	170	277	277	212	212
10-1	118	118	168	168	345	345	210	210
10-2	118	118	124	124	274	274	212	212
10-3	124	124	148	148	269	269	212	212
10-4	118	118	160	160	345	345	212	212
10-5	124	124	124	124	274	274	212	212
10-6	122	122	148	148	277	277	212	212
10-7	124	124	160	160	274	274	212	212
10-8	118	118	148	148	274	274	212	212
10-9	122	122	160	160	271	271	210	210
10-10	122	122	148	148	277	277	210	210
10-11	122	122	160	160	271	271	212	212

10-12	118	118	148	148	277	277	210	210
10-13	122	122	160	160	345	345	210	210
7561-10b	122	122	160	160	274	274	210	210
7561-11b	122	122	160	160	274	274	210	210
7561-12b	122	122	160	160	274	274	210	210
7561-1a	128	128	160	160	274	274	210	210
7561-2a	128	128	160	160	274	274	210	210
7561-3a	128	128	160	160	274	274	210	210
7561-4a	128	128	160	160	274	274	210	210
7561-5a	128	128	160	160	274	274	210	210
7561-6a	128	128					210	210
7561-7b	122	122	160	160	274	274	210	210
7561-8b	122	122	160	160	274	274	210	210
7561-9b	122	122	205	205	277	277	212	212
7566-1	122	122	148	148	274	274	210	210
7566-10	122	122	148	148	286	286	212	212
7566-2	122	122	148	148	274	274	210	210
7566-3	122	122	148	148	274	274	210	210
7566-4	122	122	128	128	274	274	210	210
7566-5	122	122	128	128	274	274	210	210
7566-6	122	122	148	148	274	274	210	210
7566-8	122	122	148	148	286	286	212	212
7566a-11	128	128	142	142	274	274	210	210
7566a-12	122	122	148	148	274	274	210	210
7566a-7	122	122	148	148	286	286	212	212
7566a-9	122	122	148	148	286	286	212	212
7568-10	114	114	170	170	274	274	210	210
7568-11	114	114	170	170	274	274	210	210
7568-12	114	114	170	170	274	274	210	210
7568-13	122	122	164	164	283	283	210	210
7568-1i	114	114	170	170	277	277	210	210
7568-1ii	128	128	160	160	283	283	210	210
7568-2	128	128	160	160	280	280	210	210
7568-3	114	114	170	170	274	274		
7568-4	114	114	170	170	274	274	210	210
7568-5	122	122	164	164	344	344	210	210
7568-6	118	118	168	168	280	280	212	212
7568-7	114	114	170	170	277	277	210	210
7568-8	114	114	170	170	274	274	210	210
7568-9	114	114	170	170	274	274	210	210
7569-1	122	122	142	142	269	269	210	210
7569-10	122	122	142	142	269	269	210	210
7569-11	122	122	142	142	269	269	210	210
7569-12	122	122	142	142	269	269	210	210
7569-13	122	122	142	142	269	269	210	210
7569-2	122	122	142	142	269	269	210	210
**7569-3**	**130**	**130**	**162**	**162**	**274**	**274**	**210**	**210**
7569-4	122	122	142	142	269	269	210	210
7569-5	122	122	142	142	269	269	210	210
7569-6	122	122	142	142	269	269	210	210
7569-8	122	122	142	142	274	274	210	210
7569-9	122	122	142	142	269	269	210	210
7570-1	122	122	160	160	271	271	212	212
7570-10	128	128	170	170	274	274	210	210
7570-11	128	128	160	168	274	274	210	212
7570-12	128	128	170	170	274	274	210	210
7570-13	122	122	170	170	271	271	208	208
7570-2	124	124	134	205	274	274	212	212
7570-3	128	128	168	168	274	274	210	210
7570-4	118	118	170	170	274	274	210	210
7570-5	124	124	164	164	277	277	212	212
7570-6	128	128	168	168	274	274	210	210
7570-7	118	118	170	170	271	271	210	210
7570-8	128	128	170	170	353	353	210	210

7570-9	128	128	170	170	274	274	210	210
7571-10	122	122	162	162	277	277	210	210
7571-12	128	128	162	162	283	283	212	212
7571-13	122	122	164	164	274	274	212	212
7571-1a	122	122	142	142	277	277	212	212
7571-2	130	130	164	164	277	277	210	210
7571-3	122	122	164	164	271	271	212	212
7571-4	122	122	142	142	274	274	212	212
7571-5	122	122	142	142	269	269	210	210
7571-6	128	128					212	212
7571-7	130	130	164	164	269	269	210	210
7571-9	122	122	164	164	269	269	212	212
7575-1	122	122	148	148	274	274	212	212
7575-2	122	122	148	148	277	277	210	210
7575-9	122	122	148	148	274	274	210	210
7575a-10	122	122	148	148	277	277	210	210
7575a-11	122	122	148	148	280	280	210	210
7575a-12	122	122	148	148	277	277	210	210
7575a-13	122	122	148	148	274	274	212	212
7575a-3	122	122	148	148	277	277	212	212
7575a-4	122	122	148	148	280	280	212	212
7575a-5	122	122	148	148	277	277	210	210
7575a-6	122	122	148	148	274	274	212	212
7575a-7	122	122	148	148	277	277	210	210
7577-1	128	128	124	124	277	277	212	212
7577-10	128	128	124	124	280	280	212	212
7577-11	122	122	124	124	277	277	210	210
7577-2	128	128	124	124	274	274	212	212
7577-3	128	128	124	124	274	274	210	210
7577-4	124	124	124	124	277	277	212	212
7577-5	128	128	124	124	274	274	210	210
7577-6	128	128	148	148	274	274	210	210
7577-7	128	128	124	124	274	274	212	212
7577-8	128	128	124	124	274	274	210	210
7577-9	128	128	124	124	269	269	212	212
7581-10	124	124	160	160	265	265	210	210
7581-11	124	124			277	277	210	210
7581-12	124	124	148	148	265	265	210	210
7581-2	124	124	154	154	274	274	212	212
7581-3	128	128	158	158	280	280	212	212
7581-4	122	122	156	156	269	269	210	210
7581-5	122	122	156	156	269	269	212	212
7581-6	112	112	160	160	274	274	210	210
7581-7	122	122	156	156	255	255	210	210
7581-8	122	122	156	156	269	269	212	212
7581-9	122	122	154	154	274	274	212	212
7583-1	122	122	148	148	271	271	208	208
7583-10	128	128	148	148	277	277	210	210
7583-11	128	128	148	148	277	277	212	212
7583-12	122	122	148	148	274	274	210	210
7583-2	122	122	148	148	271	271	208	208
7583-3	122	122	136	136	277	277	210	210
7583-4	128	128	148	148	277	277	210	210
7583-5	128	128	148	148	277	277	210	210
7583-6	128	128	148	148	277	277	212	212
7583-7	128	128	148	148	277	277	210	210
7583-8	128	128	148	148	277	277	210	210
7583-9	128	128	148	148	277	277	212	212
7584-1	122	122	168	168	274	274	208	208
7584-10	122	122	150	150	280	280	210	210
7584-11	122	122	148	148	277	277	210	210
7584-12	122	122	162	162	274	286	210	210
7584-2	122	122	148	148	271	271	210	210
7584-3	128	128	142	142	286	286	210	210

7584-4	128	128	148	148	277	277	210	210
7584-5	122	122	148	148	271	271	210	210
7584-6	122	122	162	162	274	274	210	210
7584-7	130	130	162	162	274	274	208	208
7584-8	122	122	148	148	274	274	210	210
7584-9	128	128	142	142	286	286	210	210
7586-1	118	118	126	126	271	271	210	210
7586-10	118	118	136	136	283	283	212	212
7586-11	118	118	136	136	286	286	210	210
7586-12	118	118	136	136	283	283	210	210
7586-2	118	118	160	160	283	283	210	210
7586-3	128	128	164	164	280	280	210	210
7586-4	118	118	136	136	286	286	210	210
7586-5	118	118	164	164	280	280	212	212
7586-6	118	118	136	136	277	277	210	210
7586-7	122	122	160	160	271	271	212	212
7586-8	118	118	136	136	283	283	210	210
7586-9	118	118	136	136	283	283	210	210
7588-10	122	122	142	142	277	277	212	212
7588-11	124	124	148	148	271	271	212	212
7588-12	124	124	148	148	271	271	212	212
7588-14	124	124	154	154	277	277	210	210
7588-2	122	122	142	142	277	277	210	210
7588-3	124	124	154	154	271	271	210	210
7588-4	124	124	154	154	271	271	210	210
7588-5	124	124	154	154	271	271	212	212
7588-6	124	124	154	154	271	271	212	212
7588-7	124	124	156	156	283	283	210	210
7588-8	124	124	160	160	271	271	210	210
7588-9	122	122	148	148	277	277	212	212
7594-1	122	122	170	170	277	277	210	210
7594-10	122	122	148	148	274	274	212	212
7594-11	112	112	164	164	271	271	210	210
7594-12	112	112	148	148	269	269	212	212
7594-2	128	128	172	172	274	274	208	208
7594-3	112	112	148	148	269	269	212	212
7594-4	124	124	170	170	274	274	210	210
7594-5	114	114	152	152	274	274	212	212
7594-6	114	114	150	150	271	271	212	212
7594-7	114	114	150	150	271	271	212	212
7594-8	112	112	148	148	269	269	212	212
7594-9	122	122	154	154	274	274	212	212
7595-1	114	114	162	162	271	271	210	210
7595-10	114	114	164	164	271	271	210	210
7595-11	114	114	162	162	269	269	210	210
7595-12	114	114	164	164	269	269	210	210
7595-13	114	114					210	210
7595-3	114	114	164	164	269	269	210	210
7595-4	114	114	162	162	271	271	210	210
7595-5	130	130	160	160	271	271	210	210
7595-6	114	114	164	164	269	269	210	210
7595-7	122	122	162	162	271	271	210	210
7595-8	122	122	164	164	269	269	210	210
7595-9	112	112	164	164	271	271	210	210
7596-1			160	160	280	280	210	210
7596-10	122	122	172	172	274	274	210	210
7596-11	112	112	142	142	271	271	214	214
7596-12	112	112	172	172	274	274	210	210
7596-2	112	112	148	148	274	274	210	210
7596-3	122	122	170	170	277	277	214	214
7596-4	112	112	168	168	280	280	210	210
7596-5	122	122	170	170	277	277	214	214
7596-7	122	122	160	160	277	277	210	210
7596-8	122	122			271	271	212	212

7596-9	122	122	158	158	277	277	214	214
7599-1	128	128	148	148	286	286	210	210
7599-10	122	122	168	168	271	271	210	210
7599-11	122	122	160	160	274	274	212	212
7599-12	128	128	150	150	283	283	210	210
7599-13	118	118	154	154	280	280	212	212
7599-3	128	128	150	150	283	283	210	210
7599-4	122	122	174	174	274	274	212	212
7599-5	128	128	150	150	271	271	210	210
7599-6	118	118					212	212
7599-7	122	122	160	160	277	277	212	212
7599-8	122	122	160	160	274	274	212	212
7599-9	122	122	162	162	274	274	212	212
7600-1	122	122	164	164	271	271	210	210
7600-10	128	128	162	162	277	277	212	212
7600-11	128	128	160	160	274	274	210	210
7600-2	114	114	164	164	274	274	212	212
7600-3	122	122	172	172	271	271	212	212
7600-4	114	114	164	164	274	274	212	212
7600-5	122	122	172	172	274	274	212	212
7600-6	122	122	172	172	274	274	212	212
7600-7	122	122	172	172	274	274	212	212
7600-8	128	128	162	162	277	277	212	212
7600-9	128	128	162	162	277	277	212	212
7602-10	128	128			277	277	210	210
7602-11	128	128	128	128	274	274	210	210
7602-12	128	128	128	128	274	274	210	210
7602-2	128	128	164	164	277	277	210	210
7602-4	122	122	148	148	274	274	210	210
7602-5	128	128	162	162	274	274	210	210
7602-6	128	128	160	160	274	274	212	212
7602-7	128	128	162	162	274	274	210	210
7602-9	128	128	162	162	274	274	212	212
7604-1	128	128	160	160	277	277	210	210
7604-10	122	122	162	162	274	274	210	210
7604-11	128	128	172	172	277	277	212	212
7604-12	122	122	172	172	283	283	210	210
7604-13	128	128	158	158	274	274	212	212
7604-2	128	128	160	160	274	274	212	212
7604-3	128	128	160	160	274	274	212	212
7604-4	122	122	160	160	274	274	210	210
7604-6	122	122	160	160	274	274	210	210
7604-7	128	128	148	148	277	277	212	212
7604-8	122	122			274	274	210	210
7604-9	128	128	160	160	277	277	212	212
7606-1	122	122	172	172	280	280	210	210
7606-10	128	128	164	164	274	274	210	210
7606-11	124	124	172	172	277	277	212	212
7606-12	124	124	172	172	280	280	210	210
7606-3	124	124	160	160			212	212
7606-4	122	122	160	160	274	274	212	212
7606-5	122	122	160	160	271	271	210	210
7606-6	122	122	160	160	274	274	210	210
7606-7	128	128	160	160	274	274	210	210
7606-8	128	128	160	160	274	274	210	210
7606-9	124	124	172	172	277	277	210	210
7610-1	122	122	164	164	277	277	212	212
7610-10	122	122	170	170	271	271	212	212
7610-11	122	122	154	154	274	274	210	210
7610-12	122	122	172	172	271	271	212	212
7610-2	122	122	162	162	344	344	210	210
7610-3	122	122	154	154	274	274	210	210
7610-4	122	122	160	160	269	269	210	210
7610-5	122	122	158	158	269	269	212	212

7610-6	122	122	160	160	269	269	210	210
7610-7	122	122	158	158	269	269	210	210
7610-8	122	122	160	160	269	269	210	210
7610-9	128	128	172	172	277	277	212	212
7612-1	122	122	164	164	280	280	212	212
7612-10	122	122	164	164	280	280	212	212
7612-11	128	128	164	164	274	274	210	210
7612-12	128	128	160	160	274	274	212	212
7612-13	122	122	162	162	274	274	212	212
7612-2	122	122	164	164	274	274	210	210
7612-3	122	122	162	162	274	274	212	212
7612-4	122	122	164	164	274	274	210	210
7612-5	122	122	164	164	274	274	210	210
7612-6	128	128	164	164	274	274	210	210
7612-8	128	128	160	160	274	274	212	212
7612-9	128	128	164	164	277	277	212	212
7616-1	128	128	164	164	277	277	212	212
7616-10	128	128	164	164	271	271	212	212
7616-13	128	128	148	148	277	277	212	212
7616-14	128	128	164	164	274	274	212	212
7616-1ii	122	122	164	164	271	271	210	210
7616-2	128	128	164	164	277	277	210	210
7616-3	128	128	170	170	274	274	212	212
7616-4	128	128	166	166	274	274	210	210
7616-5	128	128	150	150	280	280	212	212
7616-6	128	128	164	164	274	274	212	212
7616-7	122	122	160	160	274	274	210	210
7616-8	124	124	162	162	353	353	210	212
7616-9i	128	128	170	170	277	277	212	212
7625-1	122	122	170	170	277	277	212	212
7625-10	122	122	164	164	271	271	212	212
7625-11	114	114	170	170	345	345	210	210
7625-2	122	122	170	170	277	277	212	212
7625-4	122	122	170	170	274	274	212	212
7625-5	122	122	170	170	277	277	212	212
7625-7	122	122	170	170	274	274	210	210
7625-8	122	122	170	170	274	274	210	210
7628-1	122	122	168	168	274	274	212	212
7628-10	122	122	170	170	274	274	212	212
7628-11	122	122	168	168	274	274	212	212
7628-12	124	124	168	168	274	274	212	212
7628-2	114	114	168	168	274	274	212	212
7628-3	124	124	170	170	274	274	212	212
7628-4	124	124	170	170	277	277	212	212
7628-5	122	122	168	168	274	274	212	212
7628-6	114	114	170	170	274	274	212	212
7628-7	128	128	205	205	277	277	210	210
7628-8	122	122	170	170	274	274	212	212
7628-9	122	122	170	170	274	274	212	212
7640-1			205	205	277	277	212	212
7640-11	122	122	160	160	269	269	212	212
7640-12	122	122	160	160	280	280	210	210
7640-2	122	122	170	170	277	277	210	210
7640-3	122	122	170	170	277	277	210	210
7640-4	122	122	152	152	255	255	210	210
7640-5	124	124	146	146	280	280	210	210
7640-6	124	124	205	205	274	274	210	210
7640-7	124	124	205	205	283	283	210	210
7640-8	122	122	150	150	277	277	210	210
7640-9	122	124	124	124	277	277	210	210

Individual	GBM1459	GBM1459	GBM1419	GBM1419	GBM1018	GBM1018	GBM1208	GBM1208
1-2	160	160	115	115	264	264	146	146
1-3	160	160	100	100	264	264	146	146
1-4	160	160	100	100	264	264	146	146
1-6	160	160	100	100	264	264	146	146
1-7	160	160	105	105	264	264	146	146
1-8	160	160	130	130	260	260	146	146
1-9	160	160	100	100	264	264	146	146
1-10	158	158	130	130	255	255	146	146
1-11	158	158	130	130	264	264	144	144
1-12	160	160	130	130	260	260	146	146
1-13	160	160	100	100	266	266	146	146
4-1	160	160	100	100	268	268	144	144
4-3	160	160	100	100	268	268	144	144
4-4	160	160	100	100	264	264	146	146
4-5	160	160	100	100	264	264	146	146
4-6	160	160	100	100	268	268	144	144
4-7	160	160	100	100	268	268	144	144
4-8	160	160	100	100	264	264	146	146
4-9	160	160	95	95	262	262	146	146
4-10	160	160	95	95	264	264	146	146
4-11	160	160	95	95	264	264	146	146
4-12	160	160	95	95	264	264	146	146
4-13	160	160	95	95	272	272	146	146
7-1	160	160	100	100	272	272	146	146
7-2	160	160	100	100	272	272	146	146
7-3	158	158	100	100			146	146
7-4	160	160	100	100	272	272	146	146
7-5	158	158	100	100	264	264	144	144
7-6	160	160	100	100	264	264	144	144
7-8	160	160	100	100	272	272	146	146
7-9	160	160	100	100	272	272	144	144
7-10	160	160	100	100	272	272	146	146
7-11	160	160	100	100	272	272	146	146
7-12	160	160	100	100	272	272	146	146
9-1	160	160	100	100	260	260	144	144
9-2	158	162	100	100	260	260	150	150
9-3	164	164	100	100	260	260	144	144
9-4	156	156	105	105	260	260	144	144
9-5	164	164	100	100	260	260	144	144
9-6	162	162	90	90	260	260	150	150
9-7	162	162	90	90	260	260	148	148
9-8	160	160	90	90	260	260	144	144
9-9	162	162	95	95	255	255	150	150
9-10	164	164	90	90	260	260	150	150
9-11	162	162	90	90	260	260	148	148
9-12	164	164	100	100	260	260	144	144
10-1	160	160	130	130	260	260	148	148
10-2	158	158	90	90	260	260	148	148
10-3	160	160	100	100	260	260	150	150
10-4	160	160	130	130	260	260	144	144
10-5	164	164	100	100	260	260	144	144
10-6	158	158	100	100	260	260	146	146
10-7	158	158	100	100	260	260	150	150
10-8	158	158	100	100	260	260	150	150
10-9	158	158	100	100	260	260	144	144
10-10	158	158	100	100	260	260	150	150
10-11	158	158	100	100	260	260	144	144

10-12	158	158	100	100	260	260	150	150
10-13	158	158	90	90	260	260	144	144
7561-10b	160	160	90	90	260	260	146	146
7561-11b	160	160	90	90	264	264	146	146
7561-12b	160	160	90	90	260	260	146	146
7561-1a	160	160	130	130	260	260	144	144
7561-2a	160	160	130	130	260	260	144	144
7561-3a	160	160	130	130	260	260	144	144
7561-4a	160	160	100	100	260	260	144	144
7561-5a	160	160	130	130	260	260	144	144
7561-6a	160	160	130	130	260	260	144	144
7561-7b	160	160	90	90	260	260	146	146
7561-8b	160	160	90	90	260	260	146	146
7561-9b	158	158	90	90	260	260	154	154
7566-1	160	160	95	95	260	260	146	146
7566-10	158	158	90	90	260	260	146	146
7566-2	160	160	95	95	260	260	146	146
7566-3	160	160	95	95				
7566-4	160	160	105	105	260	260	152	152
7566-5	160	160	105	105	260	260	152	152
7566-6	162	162	90	90	260	260	144	144
7566-8	158	158	90	90	260	260	146	146
7566a-11	158	158	90	90	260	260	144	144
7566a-12	160	160	95	95	260	260	146	146
7566a-7	158	158	90	90	260	260	146	146
7566a-9	158	158	90	90	260	260	146	146
7568-10	160	160	130	130	260	260	146	146
7568-11	158	158	130	130	260	260	146	146
7568-12	160	160	130	130	260	260	146	146
7568-13	160	160	100	100	268	268	146	146
7568-1i	160	160	130	130	260	260	146	146
7568-1ii	160	160	100	100	264	264	144	144
7568-2	158	158	100	100	264	264	144	144
7568-3	160	160	95	95			144	144
7568-4	160	160	130	130	260	260	146	146
7568-5	160	160	130	130	260	260	144	144
7568-6	160	160	130	130	260	260	144	144
7568-7	160	160	130	130	260	260	146	146
7568-8	160	160	130	130	264	264	146	146
7568-9	160	160	130	130	260	260	146	146
7569-1	160	160	130	130	260	260	144	144
7569-10	160	160	130	130	260	260	144	144
7569-11	160	160	130	130	260	260	144	144
7569-12	160	160	130	130	260	260	144	144
7569-13	160	160	130	130	260	260	144	144
7569-2	160	160	130	130	260	260	144	144
**7569-3**	**160**	**160**	**115**	**115**	**264**	**264**	**146**	**146**
7569-4	160	160	130	130	260	260	144	144
7569-5	160	160	130	130	264	264	144	144
7569-6	160	160	130	130	260	260	144	144
7569-8	160	160	130	130	260	260	144	144
7569-9	160	160	130	130	260	260	144	144
7570-1	158	158	100	100	255	255	146	146
7570-10	166	166	130	130	260	260	152	152
7570-11	166	166	120	130	255	255	152	152
7570-12	166	166	130	130	260	260	152	152
7570-13	160	160	100	100	264	264	148	148
7570-2	162	162	130	130	264	264	146	146
7570-3	166	166	130	130	260	260	152	152
7570-4	164	164	100	100	255	255	144	144
7570-5	158	158	100	100	264	264	152	152
7570-6	166	166	130	130	260	260	152	152
7570-7	164	164	100	100	255	255	144	144
7570-8	158	158	100	100	260	260	150	150

7570-9	166	166	130	130	260	260	152	152
7571-10	158	158	100	100	260	260	146	146
7571-12	158	158	100	100	260	260	144	144
7571-13	158	158	100	100	260	260	144	144
7571-1a	158	158	90	90	264	264	144	144
7571-2	160	160	100	100	260	260	146	146
7571-3	158	158	100	100	260	260	144	144
7571-4	158	158	90	90	264	264	144	144
7571-5	158	158	130	130	260	260	148	148
7571-6	158	158	100	100	260	260	144	144
7571-7	162	162	100	100	268	268	148	148
7571-9	158	158	100	100	260	260	144	144
7575-1	160	160	90	90	260	260	144	144
7575-2	160	160	90	90	260	260	144	144
7575-9	160	160	90	90	260	260	144	144
7575a-10	160	160	90	90	260	260	144	144
7575a-11	160	160	90	90	260	260	144	144
7575a-12	160	160	90	90	260	260	144	144
7575a-13	160	160	90	90	260	260	144	144
7575a-3	160	160	90	90	260	260	144	144
7575a-4	160	160	90	90	260	260	144	144
7575a-5	160	160	90	90	260	260	144	144
7575a-6	160	160	90	90	260	260	144	144
7575a-7	160	160	90	90	260	260	144	144
7577-1	162	162	100	100	260	260	144	144
7577-10	162	162	100	100	260	260	144	144
7577-11	162	162	100	100	260	260	144	144
7577-2	162	162	100	100	260	260	144	144
7577-3	162	162	100	100	260	260	144	144
7577-4	156	156	95	95	260	260	144	144
7577-5	162	162	100	100	260	260	144	144
7577-6	158	158	90	90	260	260	144	144
7577-7	162	162	100	100	260	260	144	144
7577-8	162	162	100	100	260	260	144	144
7577-9	158	158	105	105	260	260	154	154
7581-10	160	160	105	105	262	262	150	150
7581-11	156	156	130	130	260	260	144	144
7581-12	156	156	130	130	260	260	144	144
7581-2	158	158	105	105	255	255	152	152
7581-3	158	158	90	90	255	255	150	150
7581-4	158	158	105	105	260	260	144	144
7581-5	158	158	105	105	260	260	144	144
7581-6	158	158	100	100			148	148
7581-7	158	158	90	90	252	252	148	148
7581-8	158	158	105	105			144	144
7581-9	158	158	100	100			152	152
7583-1	158	158	110	110	260	260	144	144
7583-10	162	162	100	100	264	264	146	146
7583-11	162	162	100	100	264	264	146	146
7583-12	162	162	100	100				
7583-2	158	158	110	110	260	260	144	144
7583-3	166	166	105	105	260	260	146	146
7583-4	162	162	100	100	264	264	146	146
7583-5	162	162	100	100	264	264	146	146
7583-6	162	162	100	100	264	264	146	146
7583-7	162	162	100	100			146	146
7583-8	162	162	100	100	264	264	146	146
7583-9	162	162	100	100	264	264	146	146
7584-1	162	162	95	95	260	260	148	148
7584-10	158	158	105	105	260	260	146	146
7584-11	158	158	90	90	260	260	146	146
7584-12	158	158	90	90	260	260	144	144
7584-2	164	164	100	100	260	260	146	146
7584-3	158	158	130	130	260	260	144	144

7584-4	164	164	100	100	260	260	146	146
7584-5	158	158	100	100	268	268	150	150
7584-6	158	158	90	90			144	144
7584-7	158	158	105	105	264	264	144	144
7584-8	158	158	100	100	268	268	150	150
7584-9	158	158	130	130	260	260	144	144
7586-1	158	158	130	130	260	260	146	146
7586-10	158	158	130	130	260	260	146	146
7586-11	158	158	130	130	260	260	146	146
7586-12	158	158	130	130	260	260	146	146
7586-2	158	158	130	130	260	260	146	146
7586-3	162	162	105	105	260	260	148	148
7586-4	158	158	130	130	260	260	146	146
7586-5	162	162	130	130	260	260	150	150
7586-6	162	162	130	130	260	260	150	150
7586-7	158	158	100	100	264	264	146	146
7586-8	158	158	130	130	260	260	146	146
7586-9	158	158	130	130				
7588-10	160	160	130	130	260	260	146	146
7588-11	158	158	130	130	260	260	144	144
7588-12	158	158	130	130	260	260	144	144
7588-14	160	160	100	100	260	260	144	144
7588-2	160	160	130	130	260	260	146	146
7588-3	158	158	130	130	260	260	144	144
7588-4	160	160	100	100	260	260	144	144
7588-5	160	160	100	100	260	260	144	144
7588-6	160	160	100	100	260	260	144	144
7588-7	160	160	100	100	260	260	144	144
7588-8	160	160	100	100	260	260	152	152
7588-9	160	160	130	130	260	260	146	146
7594-1	156	156	100	100	255	255	146	146
7594-10	158	158	130	130	255	255	146	146
7594-11	158	158	100	100	260	260	144	144
7594-12	160	160	130	130	260	260	148	148
7594-2	158	158	115	115	255	255	146	146
7594-3	160	160	130	130	260	260	148	148
7594-4	160	160	100	100	252	252	144	144
7594-5	162	162	100	100	260	260	144	144
7594-6	162	162	100	100	260	260	144	144
7594-7	162	162	100	100	260	260	144	144
7594-8	158	158	130	130	260	260	148	148
7594-9	158	158	100	100	264	264	146	146
7595-1	156	156	100	100	260	260	144	144
7595-10	156	156	100	100	264	264	144	144
7595-11	156	156	100	100	264	264	144	144
7595-12	156	156	100	100	264	264	144	144
7595-13	156	156	100	100	262	262	144	144
7595-3	156	156	100	100			144	144
7595-4	156	156	100	100	262	262	144	144
7595-5	156	156	100	100	260	260	146	146
7595-6	156	156	100	100	262	262	144	144
7595-7	162	162	130	130	268	268	144	144
7595-8	160	160	130	130	268	268	144	144
7595-9	160	160	100	100	268	268	146	146
7596-1	160	160	90	90	260	260	148	148
7596-10	158	158	100	100	255	255	146	146
7596-11	160	160	120	120	260	260	144	144
7596-12	162	162	100	100	264	264	146	146
7596-2	160	160	100	100	255	255	146	146
7596-3	160	160	100	100	255	255	146	146
7596-4	158	158	100	100	260	260	146	146
7596-5	160	160	130	130	252	252	146	146
7596-7	160	160	115	115	264	264	146	146
7596-8	160	160	100	100	264	264	144	144

7596-9	158	158	100	100	252	252	146	146
7599-1	160	160	100	100	264	264	146	146
7599-10	166	166	130	130	264	264	148	148
7599-11	160	160	100	100	264	264	146	146
7599-12	160	160	100	100	264	264	146	146
7599-13	166	166	100	100	260	260	146	146
7599-3	160	160	100	100	264	264	146	146
7599-4	160	160	100	100	264	264	146	146
7599-5	160	160	100	100	260	260	146	146
7599-6	160	160	100	100	260	260	139	139
7599-7	160	160	100	100	264	264	146	146
7599-8	160	160	130	130	264	264	148	148
7599-9	158	158	100	100	264	264	152	152
7600-1	160	160	130	130	268	268	144	144
7600-10	158	158	100	100	268	268	144	144
7600-11	160	160	130	130	260	260	144	144
7600-2	158	158	100	100	255	255	146	146
7600-3	158	160	130	130	260	260	144	144
7600-4	158	158	100	100	255	255	146	146
7600-5	160	160	130	130	260	260	154	154
7600-6	160	160	130	130	260	260	154	154
7600-7	160	160	130	130	260	260	154	154
7600-8	158	158	100	100	268	268	144	144
7600-9	158	158	100	100	268	268	144	144
7602-10	160	160	90	90			144	144
7602-11	160	160	105	105			144	144
7602-12	160	160	105	105	264	264	144	144
7602-2	160	160	105	105	264	264	144	144
7602-4	160	160	90	90			146	146
7602-5	160	160	130	130				
7602-6	160	160	130	130			144	144
7602-7	160	160	105	105				
7602-9	160	160	90	90	260	260	144	144
7604-1	160	160	100	100	260	260	144	144
7604-10	160	160	100	100	260	260	144	144
7604-11	160	160	130	130	260	260	144	144
7604-12	160	160	130	130	260	260	144	144
7604-13	160	160	130	130	260	260	144	144
7604-2	160	160	130	130	260	260	144	144
7604-3	160	160	130	130	260	260	144	144
7604-4	160	160	130	130	260	260	144	144
7604-6	160	160	130	130	260	260	144	144
7604-7	160	160	100	100	260	260	146	146
7604-8	160	160	100	100	260	260	144	144
7604-9	160	160	130	130	260	260	144	144
7606-1	158	158	100	100	260	260	148	148
7606-10	158	158	90	90	260	260	144	144
7606-11	164	164	100	100	260	260	146	146
7606-12	164	164	100	100	260	260	146	146
7606-3	164	164	130	130	266	266	144	144
7606-4	160	160	100	100	264	264	152	152
7606-5	160	160	130	130	264	264	144	144
7606-6	158	158	130	130	264	264	144	144
7606-7	160	160	130	130	260	260	144	144
7606-8	160	160	130	130	260	260	144	144
7606-9	164	164	100	100	260	260	146	146
7610-1	160	160	100	100	264	264	144	144
7610-10	158	158	115	115	260	260	146	146
7610-11	160	160	115	115	264	264	144	144
7610-12	158	158	115	115	260	260	146	146
7610-2	160	160	100	100	260	260	150	150
7610-3	160	160	100	100			144	144
7610-4	158	158	115	115	255	255	144	144
7610-5	160	160	130	130	255	255	144	144

7610-6	160	160	130	130	255	255	144	144
7610-7	162	162	90	90	264	264	146	146
7610-8	162	162	90	90	264	264	146	146
7610-9	156	156	100	100	255	255	144	144
7612-1	160	160	90	90	260	260	146	146
7612-10	160	160	90	90	260	260	146	146
7612-11	160	160	90	90	260	260	144	144
7612-12	160	160	130	130	260	260	144	144
7612-13	160	160	90	90			146	146
7612-2	160	160	90	90	260	260	144	144
7612-3	160	160	90	90	260	260	146	146
7612-4	160	160	100	100	260	260	146	146
7612-5	160	160	90	90	260	260	146	146
7612-6	160	160	130	130	260	260	144	144
7612-8	160	160	100	100	260	260	144	144
7612-9	160	160	130	130	264	264	144	144
7616-1	160	160	90	90	260	260	144	144
7616-10	160	160	90	90	260	260	144	144
7616-13	160	160	90	90	260	260	144	144
7616-14	160	160	100	100	260	260	144	144
7616-1ii	160	160	130	130	268	268	144	144
7616-2	160	160	90	90	260	260	144	144
7616-3	160	160	100	100	260	260	146	146
7616-4	160	160	90	90	260	260	144	144
7616-5	160	160	90	90	260	260	144	144
7616-6	160	160	90	90	260	260	144	144
7616-7	160	160	90	90	255	255	144	144
7616-8	160	160	90	90	260	260	146	146
7616-9i	160	160	90	90	260	260	144	144
7625-1	158	158	95	95	264	264	146	146
7625-10	160	160	95	95	264	264	146	146
7625-11	160	160	100	100	272	272	146	146
7625-2	160	160	95	95	264	264	146	146
7625-4	160	160	95	95	264	264	146	146
7625-5	160	160	95	95	264	264	146	146
7625-7	158	158	95	95	264	264	146	146
7625-8	160	160	95	95	264	264	146	146
7628-1	160	160	110	110	268	268	144	144
7628-10	160	160	110	110	268	268	144	144
7628-11	160	160	110	110				
7628-12	160	160	100	100	264	264	146	146
7628-2	160	160	100	100	262	262	146	146
7628-3	160	160	100	100	264	264	144	144
7628-4	160	160	100	100	264	264	144	144
7628-5	160	160	110	110	268	268	144	144
7628-6	160	160	100	100	268	268	144	144
7628-7	160	160	90	90	260	260	144	144
7628-8	160	160	110	110	268	268	144	144
7628-9	160	160	110	110	268	268	144	144
7640-1	162	162	100	100	255	255	152	152
7640-11	158	158	105	105	264	264	146	146
7640-12	160	160	105	105	264	264	148	148
7640-2	160	160	105	105	260	260	146	146
7640-3	158	158	100	100	252	252	148	148
7640-4	156	156	105	105	255	255	144	144
7640-5	162	162	100	100	260	260	146	146
7640-6	160	160	115	115	264	264	146	146
7640-7	162	162	105	105	264	264	146	146
7640-8	162	162	105	105	264	264	146	146
7640-9	162	162	100	100	260	260	146	146

Individual	GBM1054	GBM1054	GBM1218	GBM1218	GBM1008	GBM1008
1-2	264	264	132	132	168	168
1-3	264	264	132	132	171	171
1-4	264	264	132	132	171	171
1-6	264	264	132	132	162	162
1-7	264	264	132	132	174	174
1-8	264	264	132	132	174	174
1-9	264	264	132	132	174	174
1-10	264	264	138	138	171	171
1-11	270	270	138	138	162	162
1-12	267	267	132	132	171	171
1-13	255	255	132	132	171	174
4-1	255	255	136	136	177	177
4-3	255	255	136	136	177	177
4-4	255	255	136	136	177	177
4-5	255	255	136	136	177	177
4-6	255	255	136	136	177	177
4-7	255	255	136	136	177	177
4-8	255	255	136	136	177	177
4-9	255	255	136	136	177	177
4-10	255	255	136	136	177	177
4-11	255	255	136	136	177	177
4-12	255	255	136	136	177	177
4-13	255	255	136	136	177	177
7-1	255	255	136	136	174	174
7-2	255	255	136	136	174	174
7-3	255	255	136	136	177	177
7-4	255	255	136	136	174	174
7-5	255	255	136	136	168	168
7-6	255	255	136	136		
7-8	255	255	136	136	171	171
7-9	255	255	136	136	177	177
7-10	255	255	136	136	171	171
7-11	255	255	136	136	171	171
7-12	255	255	136	136	171	171
9-1	261	261	136	136	171	171
9-2	261	267	134	134	168	168
9-3	255	255	134	134	168	168
9-4	264	264	134	134	168	168
9-5	264	264	134	134	168	168
9-6	267	267	134	134	168	168
9-7	264	264	134	134	168	168
9-8	255	255	136	136	177	177
9-9	264	264	136	136	168	168
9-10	255	255	134	134	168	168
9-11	264	264	134	134	168	168
9-12	264	264	134	134	168	168
10-1	264	264	134	134	168	168
10-2	264	264	134	134	168	168
10-3	264	264	134	134	171	171
10-4	264	264	134	134	168	168
10-5	261	261	134	134	171	171
10-6	264	264	134	134	171	171
10-7	261	261	134	134	171	171
10-8	261	261	134	134	168	168
10-9	264	264	134	134	168	168
10-10	261	261	134	134	168	168
10-11	264	264	134	134	168	168

10-12	264	264	134	134	165	165
10-13	264	264	138	138	168	168
7561-10b	264	264	134	134	168	168
7561-11b	264	264	134	134	168	168
7561-12b	264	264	134	134	168	168
7561-1a	261	261	134	134	174	174
7561-2a	261	261	134	134	174	174
7561-3a	261	261	134	134	174	174
7561-4a	261	261	134	134	174	174
7561-5a	261	261	134	134	174	174
7561-6a	261	261	134	134	174	174
7561-7b	264	264	134	134	168	168
7561-8b	264	264	134	134	168	168
7561-9b	264	264	134	134	168	168
7566-1	261	261	134	134	171	171
7566-10	264	264	136	136	168	168
7566-2	261	261	134	134	171	171
7566-3	261	261	134	134	171	171
7566-4	264	264	136	136	180	180
7566-5	264	264	136	136	180	180
7566-6	264	264	134	134	168	168
7566-8	264	264	136	136	168	168
7566a-11	264	264	136	136	168	168
7566a-12	261	261	134	134	171	171
7566a-7	264	264	136	136	168	168
7566a-9	264	264	134	134	168	168
7568-10	270	270	134	134	168	168
7568-11	270	270	134	134	168	168
7568-12	264	264	134	134	174	174
7568-13	264	264	134	134	168	168
7568-1i	270	270	134	134	168	168
7568-1ii	267	267	134	134	165	165
7568-2	267	267	134	134	165	165
7568-3	255	261	136	136	177	177
7568-4	270	270	134	134	168	168
7568-5	261	261	134	134	174	174
7568-6	255	255	132	132	171	171
7568-7	264	264	134	134	168	168
7568-8	264	264	134	134	168	168
7568-9	270	270	134	134	168	168
7569-1	264	264	138	138	174	174
7569-10	264	264	138	138	174	174
7569-11	264	264	138	138	174	174
7569-12	264	264	138	138	174	174
7569-13	264	264	138	138	174	174
7569-2	264	264	138	138	174	174
**7569-3**	**261**	**261**	**132**	**132**	**177**	**177**
7569-4	264	264	138	138	174	174
7569-5	264	264	138	138	174	174
7569-6	264	264	138	138	174	174
7569-8	264	264	138	138	174	174
7569-9	264	264	138	138	174	174
7570-1	267	267	134	134	174	174
7570-10						
7570-11	267	267	138	138	159	159
7570-12	267	267	138	138	159	159
7570-13	264	264	134	134	174	174
7570-2	267	267	132	132	171	171
7570-3	267	267	138	138	159	159
7570-4	264	264	134	134	168	168
7570-5	267	267	134	134	168	168
7570-6	267	267	138	138	159	159
7570-7	264	264	134	134	159	159
7570-8	261	261	132	132	168	168

7570-9	267	267	138	138	159	159
7571-10	264	264	134	134	174	174
7571-12	264	264	134	134	174	174
7571-13	264	264	136	136	174	174
7571-1a	264	264	134	134	177	177
7571-2	270	270	134	134	174	174
7571-3	264	264	136	136	174	174
7571-4	264	264	134	134	177	177
7571-5	255	255	134	134	183	183
7571-6						
7571-7	270	270	132	132	177	177
7571-9	264	264	136	136	174	174
7575-1	264	264	134	134	171	171
7575-2	264	264	134	134	171	171
7575-9	264	264	134	134	171	171
7575a-10	264	264	134	134	171	171
7575a-11	264	264	134	134	171	171
7575a-12	264	264	134	134	171	171
7575a-13	264	264	134	134	171	171
7575a-3	264	264	134	134	171	171
7575a-4	264	264	134	134	171	171
7575a-5	264	264	134	134	171	171
7575a-6	264	264	134	134	171	171
7575a-7	264	264	134	134	171	171
7577-1	258	258	134	134	168	168
7577-10	258	258	134	134	168	168
7577-11	267	267	134	134	168	168
7577-2	258	258	134	134	168	168
7577-3	267	267	134	134	168	168
7577-4	267	267	134	134	168	168
7577-5	261	261	132	132	168	168
7577-6	267	267	134	134	168	168
7577-7	258	258	134	134	168	168
7577-8	258	258	134	134	168	168
7577-9	261	261	134	134	168	168
7581-10	261	261	138	138	174	174
7581-11	261	261	138	138	174	174
7581-12	261	261	138	138	174	174
7581-2	267	267	138	138	171	171
7581-3	267	267	134	134	183	183
7581-4			138	138	168	168
7581-5	261	261	138	138	168	168
7581-6	267	267	138	138	159	159
7581-7	264	264	138	138	168	168
7581-8	261	261	138	138	168	168
7581-9	264	264	138	138	159	159
7583-1	267	267	134	134	168	168
7583-10	267	267	136	136	171	171
7583-11	258	258	136	136	171	171
7583-12	264	264	132	132	171	171
7583-2	267	267	134	134	168	168
7583-3	264	264	134	134	180	180
7583-4	258	258	136	136	171	171
7583-5	258	258	136	136	171	171
7583-6	258	258	136	136	171	171
7583-7	264	264	136	136	171	171
7583-8	258	258	136	136	171	171
7583-9	258	258	136	136	171	171
7584-1	270	270	134	134	168	168
7584-10	270	270	138	138	171	171
7584-11	264	264	134	134		
7584-12	264	264	134	134	168	168
7584-2	264	264	134	134	171	171
7584-3	258	258	134	134	165	165

7584-4	258	258	134	134	174	174
7584-5	264	264	138	138	174	174
7584-6	264	264	134	134	168	168
7584-7			134	134	180	180
7584-8	264	264	138	138	177	177
7584-9	258	258	134	134	168	168
7586-1	267	267	134	134	174	174
7586-10	267	267	134	134	180	180
7586-11	267	267	134	134	174	174
7586-12	267	267	134	134	174	174
7586-2	264	264	134	134	177	177
7586-3	258	258	134	134	168	168
7586-4	267	267	134	134	174	174
7586-5	270	270	134	134	171	171
7586-6	270	270	134	134	171	171
7586-7	267	267	138	138	177	177
7586-8	267	267	134	134	174	174
7586-9	267	267	134	134	174	174
7588-10	264	264	134	134	168	168
7588-11	261	261	134	134	177	177
7588-12	261	261	134	134	177	177
7588-14	267	267	134	134	174	174
7588-2	264	264	134	134	168	168
7588-3	261	261	134	134	171	171
7588-4	261	261	134	134	168	168
7588-5	261	261	134	134	171	171
7588-6	261	261	134	134	171	171
7588-7	264	264	134	134	174	174
7588-8	267	267	134	134	171	171
7588-9	255	255	134	134	168	168
7594-1	258	258	138	138	171	171
7594-10	267	267	134	134	159	159
7594-11	264	264	134	134	171	171
7594-12	264	264	138	138	159	159
7594-2	264	264	134	134	168	168
7594-3	264	264	138	138	159	159
7594-4	264	264	138	138	171	171
7594-5	264	264	134	134	159	159
7594-6	264	264	134	134	159	159
7594-7	264	264	134	134	159	159
7594-8	273	273	138	138	159	159
7594-9	267	267	134	134	174	174
7595-1	264	264	132	132	168	168
7595-10	264	264	132	132	168	168
7595-11						
7595-12	270	270	134	134	168	168
7595-13	264	264				
7595-3	264	264	132	132	168	168
7595-4	264	264	132	132	168	168
7595-5	264	264	138	138	168	168
7595-6	264	264	132	132	168	168
7595-7	264	264	134	134	168	168
7595-8	264	264	138	138	177	177
7595-9	270	270	138	138	171	171
7596-1	264	264	138	138	159	159
7596-10	264	264	138	138	171	171
7596-11	264	264	138	138	159	159
7596-12	270	270	138	138	171	171
7596-2	264	264	134	134	171	171
7596-3	264	264	138	138	159	159
7596-4	264	264	134	134	171	171
7596-5	267	267	138	138	159	159
7596-7	264	264	138	138	159	159
7596-8	264	264	138	138	159	159

7596-9	270	270	138	138	159	159
7599-1	264	264	136	136	174	174
7599-10	267	267	132	132	174	174
7599-11	270	270	136	136	171	171
7599-12	270	270	136	136	174	174
7599-13	267	267	136	136	177	177
7599-3	270	270	136	136	174	174
7599-4	267	267	136	136	174	174
7599-5	267	267	136	136	159	159
7599-6	267	267	136	136	174	174
7599-7	270	270	136	136	171	171
7599-8	255	255	136	136	174	174
7599-9	255	255	132	132	174	174
7600-1	264	264	134	134	177	177
7600-10	273	273	134	134	162	162
7600-11	264	264	134	134	174	174
7600-2	264	264	134	134	174	174
7600-3	270	270	134	134	168	168
7600-4	264	264	134	134	168	168
7600-5	270	270	134	134	177	177
7600-6	270	270	134	134	177	177
7600-7	270	270	134	134	177	177
7600-8	267	267	134	134	162	162
7600-9	273	273	134	134	162	162
7602-10	264	264	134	134	174	174
7602-11	264	264	134	134	168	168
7602-12	264	264	134	134	168	168
7602-2	267	267	134	134	174	174
7602-4	264	264	136	136	168	168
7602-5	264	264	134	134	168	168
7602-6	264	264	134	134	174	174
7602-7	264	264	134	134	174	174
7602-9	264	264	134	134	174	174
7604-1	264	264	134	134	168	168
7604-10	267	267	134	134	168	168
7604-11	264	264	134	134	174	174
7604-12	261	261	134	134	171	171
7604-13	261	261	134	134	174	174
7604-2	264	264	134	134	174	174
7604-3	264	264	134	134	174	174
7604-4	261	261	134	134	174	174
7604-6	261	261	134	134	174	174
7604-7	264	264	134	134	174	174
7604-8	267	267	134	134	168	168
7604-9	264	264	134	134	174	174
7606-1	264	264	136	136	174	174
7606-10	264	264	134	134	174	174
7606-11	264	264	132	132	171	171
7606-12	264	264	132	132	171	171
7606-3	270	270	138	138	171	171
7606-4	264	264	134	134	171	171
7606-5	264	264	138	138	171	171
7606-6	264	264	138	138	171	171
7606-7	264	264	134	134	174	174
7606-8	264	264	134	134	174	174
7606-9	264	264	132	132	171	171
7610-1	264	264	132	132	174	174
7610-10	264	264	138	138	177	177
7610-11	270	270	138	138	171	171
7610-12	264	264	138	138	177	177
7610-2	264	264	136	136	171	171
7610-3	264	264	138	138	171	171
7610-4	264	264	138	138	171	171
7610-5	264	264	136	136	171	171

7610-6	264	264	138	138	171	171
7610-7	264	264	138	138	171	171
7610-8	264	264	138	138	171	171
7610-9	261	261	132	132	171	171
7612-1	264	264	134	134	168	168
7612-10	264	264	134	134	168	168
7612-11	264	264	134	134	168	168
7612-12	261	261	134	134	174	174
7612-13	264	264	134	134	168	168
7612-2	264	264	134	134	168	168
7612-3	264	264	134	134	168	168
7612-4	267	267	136	136	168	168
7612-5	264	264	134	134	168	168
7612-6	264	264	134	134	168	168
7612-8	264	264	134	134	171	171
7612-9	264	264	134	134	174	174
7616-1	267	267	134	134	174	174
7616-10	267	267	134	134	174	174
7616-13	267	267	134	134	174	174
7616-14	264	264	132	132	168	174
7616-1ii	264	264	134	134	177	177
7616-2	261	261	134	134	174	174
7616-3	261	261	134	134	168	168
7616-4	267	267	134	134	159	159
7616-5	267	267	134	134	174	174
7616-6	267	267	134	134	174	174
7616-7	264	264	134	134	174	174
7616-8	255	255	134	134	168	168
7616-9i	267	267	134	134	174	174
7625-1	255	255	136	136	174	174
7625-10	255	255	136	136	174	174
7625-11	255	255	136	136	174	174
7625-2	255	255	136	136	174	174
7625-4	264	264	136	136	168	168
7625-5	255	255	136	136	174	174
7625-7	255	255	136	136	174	174
7625-8	255	255	136	136	174	174
7628-1	255	255	136	136	177	177
7628-10	255	255	136	136	177	177
7628-11	255	255	136	136	177	177
7628-12	255	255	136	136	168	168
7628-2	255	255	136	136	174	174
7628-3	255	255	136	136	177	177
7628-4	255	255	136	136	177	177
7628-5	255	255	136	136		
7628-6	255	255	136	136	174	174
7628-7	267	267	132	132	177	177
7628-8	255	255	136	136	177	177
7628-9	255	255	136	136	177	177
7640-1	267	267	134	134	177	177
7640-11	264	264	134	134	171	171
7640-12	264	264	134	134	171	171
7640-2	264	264	134	134	171	171
7640-3	264	264	136	136	174	174
7640-4	264	264	134	134	168	168
7640-5	264	264	134	134	171	171
7640-6	264	264	136	136	171	171
7640-7	267	267	136	136	180	180
7640-8	261	261	136	136	168	168
7640-9	261	261	134	136	168	171
