# Supplementary material for: Geography of Genetic Structure in Barley Wild Relative Hordeum vulgare subsp. spontaneum in Jordan
Source: PLoS One. 2016 Aug 11;11(8):e0160745. doi: 10.1371/journal.pone.0160745 (PMC4981475; doi:10.1371/journal.pone.0160745)
Supplement: S2 Table — (PDF) [file pone.0160745.s003.pdf]

S2 Table. Ecogeographical and genetic data for 32 *Spontaneum* populations collected in Jordan in 2012

Geography of genetic structure in barley wild relative *Hordeum vulgare* subsp. *spontaneum* in Jordan

PloS ONE

Thormann I. et al.

Site number	Collecting number	Latitude	Longitude	Elevation (m)	Slope as recorded during collecting	Slope as extracted from DEM	Aspect as recorded during collecting
1	9	32.70233	35.72325	94	undulating 3-8	6.4072709	E
2	10	32.69611	35.7375278	119	gently rolling 8-16	2.0650151	E
3	7577	32.67656	35.8048333	467	level 0-3	2.094852	F
4	7581	32.59239	35.6669444	87	undulating 3-8	2.408005	E
5	7575	32.58686	35.9981944	475	level 0-3	0.6448038	F
6	7583	32.51183	35.6454444	119	sloping 16-30	5.736351	S
7	7561	32.47747	35.9696944	608	level 0-3	0.8024288	F
8	7584	32.43733	35.6918056	484	gently rolling 8-16	2.7060931	W
9	7586	32.37594	35.7365	785	undulating 3-8	2.862745	N
10	7571	32.33386	35.91375	957	gently rolling 8-16	2.789247	N
11	7566	32.32931	36.0926667	866	level 0-3	2.2942541	F
12	7588	32.32144	35.7501389	770	sloping 16-30	4.5872722	W
13	7640	32.27547	35.8913333	564	undulating 3-8	3.0051081	E
14	7570	32.23847	35.8894722	379	undulating 3-8	2.4141221	E
15	7569	32.14508	35.8566389	561	gently rolling 8-16	2.2644801	N
16	7599	32.13858	35.6468056	141	gently rolling 8-16	6.0931702	W
17	7568	32.11461	35.86625	629	undulating 3-8	4.2316108	E
18	7600	32.06989	35.7151389	1044	gently rolling 8-16	4.86167	W
19	7595	32.06694	35.7205833	1058	level 0-3	1.595687	F
20	7594	32.04581	35.7751667	911	level 0-3	1.337201	F
21	7596	32.01156	35.7337778	589	level 0-3	3.749177	F
22	7602	31.77919	35.7988333	805	level 0-3	0.8993503	F
23	7616	31.70953	35.9606111	709	level 0-3	0.3275474	F
24	7604	31.67036	35.7858889	745	level 0-3	0.3143624	F
25	1	31.56639	35.7914167	646	undulating 3-8	2.3862491	W
26	7606	31.54019	35.7736389	677	undulating 3-8	3.9107339	E
27	7610	31.18831	35.6965833	773	sloping 16-30	5.5509439	N
28	7612	31.04872	35.7088611	1222	level 0-3	0.3617871	F
29	7628	30.68108	35.6222222	1566	level 0-3	6.893683	F
30	4	30.65542	35.6101944	1257	sloping 16-30	8.1156244	S
31	7625	30.42178	35.512	1580	level 0-3	1.376532	F
32	7	30.39875	35.4968611	1680	level 0-3	2.840307	F

Site number	Aspect as extracted from DEM	Geology	Soiltexture	Soil colour	Mean annual temperature	Mean annual rainfall	Habitat	Number of individuals for analysis
1	302.355194	Limestone	clay-loam	brown	19.97	403	Shrubland	12
2	312.273712	Limestone	clay-loam	brown	19.97	403	Reserve	13
3	10.3992395	Limestone	clay-loam	terra rosa	18.58	408	Roadside	11
4	285.995789	Limestone	clay-loam	brown	20.34	380	Roadside	11
5	286.699188	Limestone	clay-loam	brown	17.81	317	Field margin	12
6	302.176514	Limestone	silt	beige	21.09	336	Roadside	12
7	348.906311	Limestone	clay-loam	terra rosa	17.48	322	Roadside	12
8	262.793915	Limestone	clay-loam	light brown	18.65	456	Shrubland	12
9	294.009491	Limestone	clay-loam	terra rosa	17.46	486	Roadside	12
10	338.75769	Limestone	clay-loam	terra rosa	15.94	393	Roadside	11
11	11.8390303	Limestone	clay-loam	light brown	16.21	241	Field margin	12
12	267.786285	Limestone	clay-loam	terra rosa	15.62	460	Roadside	12
13	152.316803	Limestone	clay-loam	terra rosa	17.78	366	Shrubland	11
14	193.680893	Limestone	clay-loam	terra rosa	18.86	274	Roadside	13
15	341.318115	Limestone	clay-loam	terra rosa	17.73	374	Roadside	12
16	299.574402	Limestone	silt	beige	21.47	258	Reserve	12
17	199.688904	Limestone	clay-loam	terra rosa	17.68	373	Field margin	14
18	271.543213	Limestone	clay-loam	terra rosa	15.90	471	Roadside	11
19	192.2883	Limestone	clay-loam	terra rosa	15.90	471	Field margin	12
20	2.97802091	Limestone	clay-loam	terra rosa	16.63	479	Roadside	12
21	193.436203	Limestone	clay-loam	terra rosa	16.90	491	Roadside	11
22	191.888702	Limestone	clay-loam	light brown	16.35	378	Roadside	9
23	225	Limestone	clay-loam	terra rosa	15.76	229	Roadside	13
24	24.6768608	Limestone	clay-loam	terra rosa	16.44	352	Field margin	12
25	193.272293	Limestone	silt	beige	17.80	263	Reserve	11
26	289.577911	Limestone	loam	terra rosa	17.08	274	Roadside	11
27	314.887695	Limestone	silt	beige	16.90	315	Roadside	12
28	50.1944313	Limestone	clay-loam	terra rosa	15.62	378	Roadside	12
29	268.658905	Limestone	silt	grey	14.22	313	Field margin	12
30	231.830597	Sandstone	sandy-loam	beige	14.64	291	Reserve	12
31	42.7275505	Limestone	silt	light brown	12.55	297	Roadside	8
32	252.942993	Limestone	silt	light brown	13.70	258	Reserve	11

Site number	Number of multi-locus genotypes	No of alleles	No of private alleles	Mean allelic richness	Observed heterozygosity (Ho)	Genetic cluster K = 2	Genetic cluster K = 3	Genetic cluster K = 4	Mean plant height (cm)
1	12	105	1	2.69	0.0045	1	1	1	150
2	13	120	2	3.08	0.0042	1	1	1	60
3	11	100	0	2.58	0.0000	1	1	1	60
4	11	117	2	3.05	0.0000	1	1	1	100
5	**11**	52	0	1.38	0.0000	2	2	3	60
6	12	95	0	2.46	0.0000	1	1	4	70
7	12	73	1	1.90	0.0068	2	2	3	80
8	12	126	3	3.24	0.0045	1	1	1	80
9	12	107	4	2.70	0.0000	1	1	1	60
10	11	112	1	2.92	0.0049	1	1	4	80
11	12	106	0	2.74	0.0023	2	2	3	70
12	12	109	1	2.83	0.0023	1	1	4	60
13	11	119	1	3.08	0.0150	1	1	4	85
14	13	111	2	2.82	0.0249	1	1	4	70
15	**11**	71	1	1.79	0.0023	2	2	4	90
16	12	118	4	3.02	0.0000	1	1	4	60
17	14	125	1	3.08	0.0019	1	1	4	90
18	**10**	105	1	2.76	0.0025	1	1	4	100
19	12	109	1	2.81	0.0000	1	1	1	70
20	12	130	0	3.30	0.0023	1	1	4	80
21	11	123	1	3.18	0.0027	1	1	4	60
22	9	92	0	2.46	0.0030	2	2	3	75
23	13	115	2	2.82	0.0042	2	2	3	50
24	12	94	0	2.42	0.0045	2	2	3	80
25	11	107	1	2.77	0.0025	1	1	4	25
26	11	113	0	2.94	0.0049	1	1	4	35
27	12	111	0	2.83	0.0045	1	1	4	80
28	12	97	0	2.52	0.0000	2	2	3	70
29	12	93	3	2.36	0.0023	1	3	2	30
30	12	69	1	1.83	0.0000	1	3	2	20
31	8	67	0	1.81	0.0135	1	3	2	25
32	11	76	0	2.01	0.0049	1	3	2	20
